# What are small, medium and large effect sizes for exercise treatments of tendinopathy? A systematic review and meta-analysis

**DOI:** 10.1136/bmjsem-2022-001389

**Published:** 2023-02-27

**Authors:** Paul A Swinton, Joanna S C Shim, Anastasia Vladimirovna Pavlova, Rachel Moss, Colin Maclean, David Brandie, Laura Mitchell, Leon Greig, Eva Parkinson, Victoria Tzortziou Brown, Dylan Morrissey, Lyndsay Alexander, Kay Cooper

**Affiliations:** 1 School of Health Sciences, Robert Gordon University, Aberdeen, UK; 2 Library Services, Robert Gordon University, Aberdeen, UK; 3 Sportscotland Institute of Sport, Stirling, UK; 4 Physiotherapy Services, NHS Grampian, Aberdeen, UK; 5 Barts and The London School of Medicine and Dentistry Blizard Institute, London, UK

**Keywords:** meta-analysis, rehabilitation, statistics, tendinopathy

## Abstract

**Objective:**

To quantify and describe effect size distributions from exercise therapies across a range of tendinopathies and outcome domains to inform future research and clinical practice through conducting a systematic review with meta-analysis.

**Design:**

Systematic review with meta-analysis exploring moderating effects and context-specific small, medium and large thresholds.

**Eligibility criteria:**

Randomised and quasi-randomised controlled trials involving any persons with a diagnosis of rotator cuff, lateral elbow, patellar, Achilles or gluteal tendinopathy of any severity or duration.

**Methods:**

Common databases, six trial registries and six grey literature databases were searched on 18 January 2021 (PROSPERO: CRD42020168187). Standardised mean difference (SMD_pre_) effect sizes were used with Bayesian hierarchical meta-analysis models to calculate the 0.25 (small), 0.5 (medium) and 0.75 quantiles (large) and compare pooled means across potential moderators. Risk of bias was assessed with Cochrane’s Risk of Bias tool.

**Results:**

Data were obtained from 114 studies comprising 171 treatment arms 4104 participants. SMD_pre_ effect sizes were similar across tendinopathies but varied across outcome domains. Greater threshold values were obtained for self-reported measures of pain (small=0.5, medium=0.9 and large=1.4), disability (small=0.6, medium=1.0 and large=1.5) and function (small=0.6, medium=1.1 and large=1.8) and lower threshold values obtained for quality of life (small=−0.2, medium=0.3 and large=0.7) and objective measures of physical function (small=0.2, medium=0.4 and large=0.7). Potential moderating effects of assessment duration, exercise supervision and symptom duration were also identified, with greater pooled mean effect sizes estimated for longer assessment durations, supervised therapies and studies comprising patients with shorter symptom durations.

**Conclusion:**

The effect size of exercise on tendinopathy is dependent on the type of outcome measure assessed. Threshold values presented here can be used to guide interpretation and assist with further research better establishing minimal important change.

**WHAT IS ALREADY KNOWN ON THIS TOPIC**

- Exercise therapy, in particular resistance exercise, is frequently used in the management of tendinopathy and is known to have general effectiveness across a range of important outcome domains. There is, however, a lack of research comparing effectiveness across different tendinopathies and outcome domains.

**WHAT THIS STUDY ADDS**

- This large and comprehensive meta-analysis shows that exercise therapy results in relatively wide change distributions relative to baseline. Distributions of standardised mean difference effect sizes appear consistent across the most common tendinopathies. In contrast, substantive differences exist in the distributions of standardised mean difference effect sizes across outcome domains.

**HOW THIS STUDY MIGHT AFFECT RESEARCH, PRACTICE OR POLICY**

- The results of this study provide researchers and clinicians with important information regarding how individuals should be expected to respond to exercise therapy for the management of tendinopathies, thereby influencing decisions regarding the effectiveness of any intervention and how to power future research studies.

## Background

Tendinopathy is a common musculoskeletal condition associated with degenerative changes and characterised by a combination of pain impaired movement and reduced function that typically requires extended periods for recovery.^1–5^ Tendinopathy can affect any muscle-tendon unit in the body, however, it is most frequently reported in the Achilles, patellar, lateral elbow, rotator cuff and hip tendons.^6^ Surveys of prevalence of lower extremity tendinopathy in the general population have reported rates of 11.8 and 10.5 per 1000 person-years,^7^ while prevalence for upper limb tendinopathies have been estimated between 1.3% and 21.0%.^8–10^ Costs to the individual, the health service and economy (due to absenteeism and loss of productivity) are substantial such that identifying effective interventions is a priority.

Exercise therapy is the mainstay of conservative management of tendinopathy and has focused largely on resistance training, and in many instances eccentric strengthening techniques.^11^ The rationale of exercise therapy is to improve load tolerance and possibly structural adaptation of the musculotendinous unit to restore function.^12 13^ In the early phase of rehabilitation, flexibility exercises are often initiated and incorporated into strengthening regimes to facilitate improvements in mobility.^11^ Effective exercise therapy may also require targeting a range of contributing factors, which not only include muscle weakness and decreased flexibility, but also corticospinal and neuromuscular adaptations resulting from persistent pain.^14^ As such, proprioceptive exercise interventions have been used to retrain normal patterns of muscle recruitment in the rehabilitation of shoulder-related tendinopathies including impingement.^14–17^ The time course of tendinopathy recovery is usually slow, the degree of recovery may be incomplete and there may be differences between tendinopathies and across outcome domains. For example, quality of life may improve less quickly for people with rotator cuff tendinopathy compared with those with Achilles tendinopathy, whereas recovery may be faster for pain and the relative magnitudes of these improvements may not be equivalent. Quantifying any tendinopathy or domain-specific differences in expected improvements would help guide efforts to develop consensus concerning optimal management by enabling better intervention comparisons.

A recent scoping review identified a lack of effective tools to draw general conclusions across the large tendinopathy and exercise therapy research base (~450 primary studies), which featured a wide range of interventions across different tendinopathies, populations and outcome domains.^18^ At present, one of the main tools to synthesise information and therein draw general conclusions include the use of meta-analyses. Most previous meta-analyses have attempted to quantify the effectiveness of interventions using standardised mean difference (SMD) effect sizes and Cohen’s standard benchmarks (small=0.2, medium=0.5 and large=0.8) irrespective of the tendinopathy location, population or outcome domain.^19–27^ Despite Cohen’s recommendations that these general benchmarks should only be used where more relevant context-specific information is unavailable,^28^ use of these standard benchmarks is ubiquitous through behavioural, social and health sciences. However, recent attempts have been made across a range of disciplines to use empirically derived effect size distributions to generate context-specific benchmarks providing better means of establishing the effectiveness of different interventions and drawing general conclusions.^29–35^ Results have frequently demonstrated substantive differences between Cohen’s benchmarks and those derived empirically, with examples of both underestimation and overestimation, and even differences across subdomains within a discipline.^29^ In addition, SMDs are used frequently as a means of informing the minimal important change (MIC) for patients, especially when preferred anchor-based approaches using external criterions such as global ratings of change are not available.^36^ Given the range of tendinopathies and outcome domains commonly investigated, there is potential that the distribution and subsequent appropriate interpretation of therapy effects will be diverse and could benefit from the generation of context-specific benchmarks. Therefore, the purpose of this meta-analysis was to perform a large synthesis of the available research creating empirically derived thresholds to benchmark the effectiveness of exercise therapies and explore potential differences across tendinopathies and outcome domains. The analysis also investigated the potential for moderating effects of commonly reported features including assessment duration of outcomes, therapy supervision (supervised vs unsupervised) and symptom duration of patients. The results of this analysis will provide clinicians and researchers with tendinopathy-specific and domain-specific indicators of effect sizes with which to better interpret intervention outcomes.

## Methods

This meta-analysis is part of a project funded by the National Institute for Health Research; Health Technology Assessment 129 388 Exercise therapy for the treatment of tendinopathies. The inclusion criteria were influenced by the project aims, the results of our initial scoping review mapping the exercise and tendinopathy literature, as well as stakeholder workshops. The overall structure of systematic reviews and meta-analyses to address effectiveness were registered in the PROSPERO database (CRD42020168187) and individual full protocols made publicly available prior to any analyses.^37^ ^38^ The review was conducted according to the PRISMA 2020 statement with checklist provided in online supplemental file 1.

10.1136/bmjsem-2022-001389.supp1 Supplementary data

### Protocol deviations

Multiple protocol deviations occurred due to pragmatic considerations and reflecting on processes from previous work packages in the larger project. Originally, it was intended to extract data not in duplicate but to quantify reliability based on a random 10% sample. Given the large number of reviewers extracting data it was decided to perform extraction in duplicate with agreement following differences when required. Originally, it was intended to conduct risk of bias using the ROBINS-I tool for quasi-experimental studies.^39^ As outlined in the following sections, due to pragmatic considerations Cochrane’s Risk of Bias tool was used for both randomised and non-randomised designs.^40^

### Stakeholder involvement

People with lived experience of exercise for tendinopathy were involved in all stages of the review, from inception to dissemination activities, and were recruited via National Health Service (NHS) public involvement networks and social media. Two people who had received exercise for Achilles tendinopathy and rotator cuff related shoulder pain (RCRSP) in NHS and private settings contributed to the design stage by influencing the review questions. One of these people (female, RCRSP) went on to contribute to the oversight committee throughout the review and assisted with reviewing dissemination materials. We also held a stakeholder workshop to inform the direction of the review; four females with lived experience of RCRSP or patellar tendinopathy, including one high performance athlete, took part. We had anticipated greater public involvement; timing of activities (during COVID-19 lockdown) and conducting them solely online may be contributing factors. Nonetheless, contributions were helpful in informing the review.

### Inclusion criteria

#### Participants

This meta-analysis included people of any age or gender with a diagnosis of RCRSP, lateral elbow, patellar, Achilles or gluteal tendinopathy of any severity or duration. Studies that included participants with tendinopathy in the absence of full thickness or large tears were included. We accepted trial authors’ diagnoses where a clearly verifiable group of clinical features is reported including: pathognomonic location of pain; a symptom altering response to applied load and/or stretch, with there being a specific test for most tendinopathies; strategies to rule out differential diagnoses; ultrasound or MRI confirmation of structural change. We included studies with mixed groups where there was clear reporting of the tendinopathic group, or those participants comprised >90% of the investigated cohort.

#### Intervention

The intervention being assessed is exercise therapy comprising five different therapy classes: (1) resistance, (2) plyometric, (3) vibration, (4) flexibility and (5) proprioception. Definitions for each therapy class are presented in online supplemental file 2. Interventions combining exercise with other active therapies (eg, laser, shockwave, manual therapy or injection) were not included. We included exercise therapies delivered in a range of settings and delivered by a range of health, exercise professionals or support workers. We also included both supervised and unsupervised exercise therapies. As part of the inclusion criteria, we required studies to report sufficient information regarding the exercise intervention to enable appropriate identification of treatment duration, therapy class and exercise dose. In clinical settings, it has been recommended that exercise dose is determined by duration, frequency and intensity. To be included in the review, we required studies to provide sufficient information to describe at least two of the three exercise dose parameters. Where sufficient information was not presented in the main text of a study, a search was made of the publishers’ website to check for online supplemental files that may include relevant information.

10.1136/bmjsem-2022-001389.supp2 Supplementary data

#### Comparator

No comparators were included, with effect sizes used to quantify the intervention effectiveness of exercise only therapies based on change relative to baseline.

#### Outcomes

Based on the results of our initial scoping review and subsequent stakeholder workshops,^18^ we included outcomes that assessed six domains: (1) disability, (2) physical function capacity (PFC), (3) function, (4) pain (on loading/activity, over a specified time or without further specification), (5) quality of life and (6) range of motion (ROM) (shoulder joint only). Definitions of each domain and example tools are presented in online supplemental file 3.

#### Types of studies

We included randomised controlled trials (RCTs) and non-RCTs where at least one intervention arm comprised an exercise therapy that could be categorised according to the therapy classes outlined.

#### Context

The context included primary care, secondary care or community locations in nations defined as very high or high on the Human Development Index (top 62 countries at the time of protocol development) for the findings to be relevant to the UK context.^41^

### Exclusion criteria

In addition to coding treatment arm interventions according to exercise criteria, we also coded non-active (eg, wait-and-see, placebo and sham) and non-exercise (eg, electrotherapy, biomechanics, manual therapy, injection therapy and surgery) categories according to preset definitions (online supplemental file 4). Where any of the non-active or non-exercise categories could be assigned to a treatment arm intervention, these were excluded from the review.

### Search strategy

The search strategy used for this study was part of a larger search conducted to scope the entire tendinopathy and exercise therapy research base.^18^ The search comprised three steps. First, a limited search of MEDLINE and CINAHL using initial keywords was conducted to develop a full search strategy. Second, the full search strategy was adapted to each database and applied systematically to: MEDLINE, CINAHL, AMED, EMBase, SPORTDiscus, Cochrane library (Controlled trials, Systematic reviews), JBI Evidence Synthesis, PEDRo and Epistemonikos (search terms for each database are presented in online supplemental file 5. In addition, six trial registries, five grey literature data bases and Google Scholar were searched (online supplemental file 5). Finally, the third step involved conducting a search of cited and citing articles using Scopus and handsearching a total of 130 systematic reviews that were identified to include information relevant to exercise therapy and tendinopathy. No limit was placed on language, with research studies reported in languages other than English translated via Google Translate or via international collaborations of the review team members. Searches were initiated from 1998 as (1) the heavy load eccentric calf-training protocol for Achilles tendinosis by Alfredson *et al* was published in 1998 and may be considered seminal work in the field of tendinopathy^42^ and (2) there has been a proliferation of research on exercise interventions for tendinopathies post 1998. The final date of the search was 18 January 2021.

### Study selection

Proquest Refworks was used to manage references and remove duplicates before importing to Covidence (Melbourne, Australia) to facilitate screening and initiate a second deduplication process. Titles/abstracts were reviewed, independently, by two members of the research team. Full-text copies of all studies included at title/abstract screening stage were retrieved and also reviewed, independently, by two members of the research team. Conflicts were resolved by discussion or by input from a third reviewer.

### Data extraction

Data were extracted independently by eight members of the review team (PAS/KC/LA/RM/LG/EP/JSCS/AVP) into preiloted Excel sheets. Data were independently coded as described in the accompanying extraction codebook (online supplemental file 6) by two members from the review team. Differences in entries were detected through automatic checking in MS Excel and then agreed between the same two reviewers. Where pre–post intervention data were not presented in text but in figures, data were extracted using digitsation software (PlotDigitizer V.2.6.8 Windows).

### Risk of bias

We used Cochrane’s Risk of Bias tool^40^ and six outcome domains: (1) selection bias (random sequence generation and allocation concealment), (2) performance bias (blinding of participants), (3) detection bias (blinding of outcome assessors), (4) attrition bias (incomplete outcome data), (5) reporting bias (selective reporting) and (6) other bias, to assess risk of bias for RCTs and domains 2–6 for non-randomised trials. Risk of bias was recorded for each outcome and time point within each study. When obtaining a summary risk of bias for each domain within a study, the mode category across all outcomes and timepoints was selected. The Cochrane’s Risk of Bias tool^40^ was selected as a recent review of popular risk of bias tools in tendinopathy management highlighted none were superior^43^ and Cochrane’s Risk of Bias tool could be semiautomated with RobotReviewer,^44^ a machine learning system software. RobotReviewer was used to make initial assessments on selection bias and performance bias domains, with manual validation made on the relevant free texts extracted to support the final selection of low, high or unclear risk of bias. This semiautomated process was more efficient, and pragmatic given the large number of included studies and provided an additional element of consistency in the review process.

### Statistical analysis overview

In general, meta-analyses attempt to pool data across studies quantifying effectiveness by focusing on a weighted mean value. Due to the use of different outcome domains and different tests within a specific outcome domain, pooling of data requires initial standardisation. The most common metric used to standardise effectiveness is the SMD_pre_ effect size, which divides the group change by the preintervention SD. Meta-analyses seek to describe the underlying population effect size based on a normal distribution with the mean representing the most likely value across studies, and the SD representing the dispersion that can be expected across individual studies. However, a focus on a single central value provides limited description of the overall distribution that can be expected. By providing estimates of the 0.25 quantile (the value which 25% of observed results are expected to be below), the 0.5 quantile and the 0.75 quantile, a more detailed description is obtained. In addition, these estimates can be used to provide benchmarks to interpret the effectiveness of future interventions, with the traditional qualitative labels of ‘small’ (0.25 quantile), ‘medium’ (0.5 quantile) and ‘large’ (0.75 quantile) used to provide a simple scale.

Most meta-analyses are conducted within a frequentist framework where a focus is placed on the mean value and hypothesis testing with CIs to identify whether they overlap a zero effect. In contrast, analyses performed within a Bayesian framework are more flexible enabling, for example, quantile values to be estimated and can be interpreted intuitively through reporting of subjective probabilities.^45^ Given the purpose of the present meta-analysis to describe the overall distribution of effect sizes and explore differences among potentially relevant factors (eg, tendinopathy location and outcome domains), a Bayesian framework was selected. Finally, many meta-analyses only extract a single outcome from each study per-analysis to avoid complexities due to relationships within data. However, more precise estimates of effects may be obtained by including all relevant data but accounting for covariances of multiple study outcomes within the meta-analysis model. A recommended approach to account for hierarchical structures (eg, multiple measurements from the same study, multiple measurements made from difference outcomes within the same study and multiple measurements made from the same outcome in the same study) is to apply multilevel meta-analysis models, which were included for the present study.

### Statistical analysis details

SMD_pre_ effect sizes were calculated by dividing the relevant mean difference by the preintervention SD and including a small sample bias correction.^46^ Where required, SMD_pre_ values were reflected by multiplying by –1 to ensure that positive values represented an improved clinical effect. Where outcomes were assessed at multiple time points following baseline measurement, all possible SMD_pre_ values were calculated and included in the meta-analysis models. Where means and SDs were not presented but included combinations of the median, range or IQR, values were estimated by the calculations presented by Wan *et al*.^47^ Where sufficient information was not available to estimate SDs, these were imputed through simple linear regression of the log transformed SD and means obtained from all other studies.^48^ Separate regressions were performed for preintervention and postintervention data.

All meta-analyses were conducted using a nested four-level model.^49^ The series of nestings included the individual study (level 4), the outcome (level 3), the measurement occasion (level 2) and the sampling variance (level 1). Standard distributional assumptions were used to calculate the sampling variance of SMD_pre_ values.^46^ However, the calculation requires an estimate of the pre–post correlation which is rarely reported in studies. To account for uncertainty in the sampling variance, values within the model were allowed to vary and were estimated by including an informative Gaussian prior approximating correlation values centred on 0.7 and ranging from 0.5 to 0.9.^49^ The relative contributions of variance sources were described by variance partition coefficients (VPCs) which were calculated by dividing each estimated variance level by the total sum. Therefore, the higher the VPC for the outcome and measurement levels the greater the covariances within the data. Analyses were only completed where a minimum of 50 effect sizes were available to appropriately describe distributions. Sensitivity analyses checking the potential influences of study type (RCTs vs quasi-RCTs) and study quality (low risk of bias: >50% ‘low risk’ vs high risk of bias: 50% ‘low risk’) on effect size thresholds were conducted through subset comparisons.

It was determined a priori to assess the influence of tendinopathy location, outcome domain, assessment duration, symptom duration and supervised versus non-supervised exercise on effect sizes. This was achieved by subset comparisons for tendinopathy location and outcome domain, and meta-regressions for assessment duration, symptom duration and supervision. Meta-regressions were presented by selecting one level of the factor as a reference to make comparisons with the median and 95% CrI (β_Reference:Comparison_ = Median (95%CrI: lower bound to upper bound), such that β>0 indicates an increased effect of the comparison relative to the reference).

The importance of removing outliers to obtain accurate estimates of meta-analysis parameters was identified in a previous and similar large meta-analysis of exercise SMD_pre_ values.^49^ Outlier SMD_pre_ values were identified by adjusting the empirical distribution by a Tukey g -and-h distribution and obtaining the 0.0125 and 0.9875 quantiles, with values beyond these points removed prior to further analysis.^50^ Meta-analyses were conducted using the R wrapper package brms interfaced with Stan to perform sampling.^51^ Convergence of parameter estimates were obtained for all models with Gelman-Rubin R-hat values below 1.1.^52^ Data used for the analyses and R code are presented in online supplemental files 7 and 8.

## Results

### Study selection

**Figure 1:**
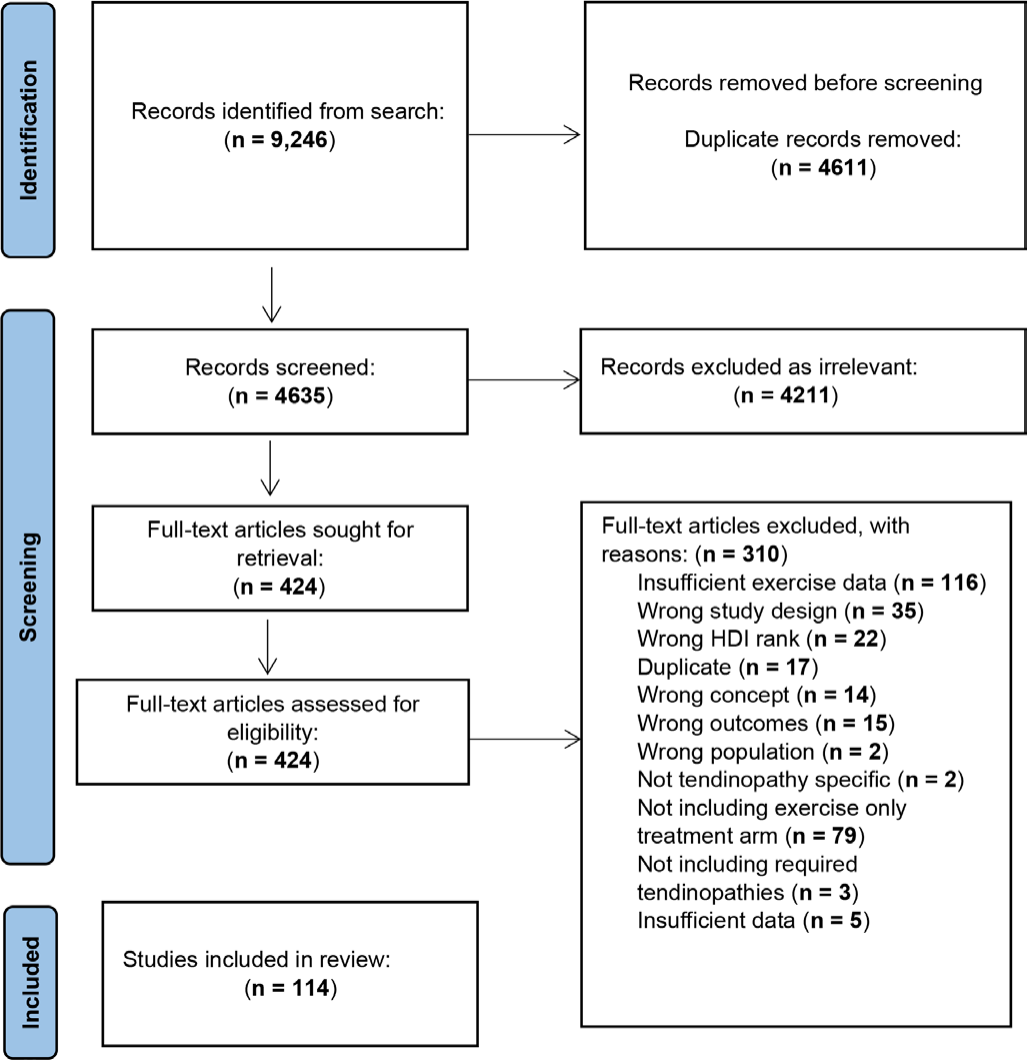
Preferred Reporting Items for Systematic Reviews and Meta-Analyses (PRISMA) flow diagram describing study selection.

The search strategy identified a total of 9246 potential studies, with 4635 remaining following removal of duplicates (figure 1). After title and abstract screening 4210 studies were removed leaving 425 studies obtained for full text screening. Of these studies, a further 311 were excluded based primarily on insufficient description of the exercise stimulus (116 studies) and not including exercise-only treatment arms (75 studies). In total, data from 114 studies (100 RCTs and 14 quasi-experimental) comprising 171 treatment arms and 4104 participants were included in the meta-analyses. A table of includes studies along with reference lists of included and excluded studies are presented in online supplemental files 9 and 10. Risk of bias expressed for each individual study are presented in online supplemental file 11 with summaries presented in table 1. For RCTs, risk of bias was highest for ‘other bias’ (47% high risk of bias), blinding of participants (42% high risk of bias) and selective reporting (67% unclear risk of bias). For quasi-experimental trials, risk of bias was also highest for ‘other bias’ (77% high risk of bias) and reporting quality was also lower with high percentages of unclear risk of bias identified for selective reporting (85% unclear risk of bias) and blinding (participants: 39% unclear risk of bias; outcome assessors: 61% unclear high risk of bias).

**Table 1:** Risk of bias assessment for randomised (top; N=100) and non-randomised trials (bottom; N=14) with percentages of low-risk, unclear-risk and high-risk evaluations expressed relative to the number of treatment arms (upper value) and the total number of data points (lower value)

		Random sequence allocation	Allocation concealment	Blinding of participants	Blinding of outcome assessors	Incomplete outcome bias	Selective reporting	Other bias
Randomised controlled trials	Low risk	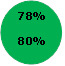	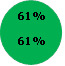	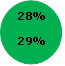	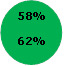	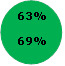	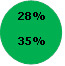	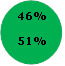
	Unclear	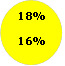	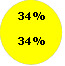	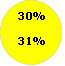	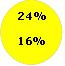	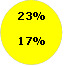	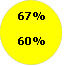	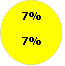
	High risk	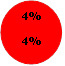	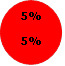	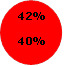	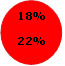	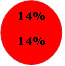	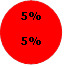	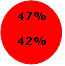
Non-randomised controlled trials	Low risk	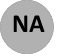	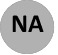	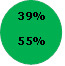	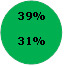	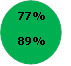	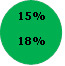	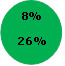
	Unclear	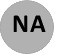	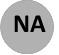	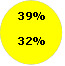	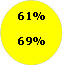	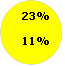	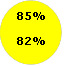	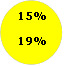
	High risk	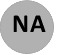	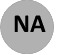	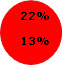	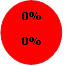	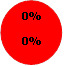	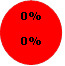	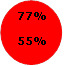

Descriptions of the study characteristics, tendinopathy location and outcome domains are presented in table 2. Outcomes obtained from studies investigating gluteal tendons were not included in the analysis based on the number of effect sizes falling below the a priori threshold set to generate accurate estimates of the population effect size distribution.

**Table 2:** Distribution (percentiles) of study characteristics, tendinopathy locations and outcome domains calculated across treatment arms

Study characteristic	0%	10%	20%	30%	40%	50%	60%	70%	80%	90%	100%
Participants per group	5	10	13	16	19	20	24	28	31	44	70
Mean age	22.5	29.5	39.8	42.6	44.0	46.0	47.9	48.7	49.9	51.9	62.1
Mean symptom duration (months)	0.85	4.4	6.0	7.8	11.6	17.5	19.5	24.1	29.7	37.4	98.5
Publication year	1998	2005	2007	2009	2012	2014	2015	2017	2017	2019	2020
Intervention length (weeks)	2	4	4	4	6	8	12	12	12	12	21
Measurement duration (weeks)	0.7	3	4	4	5	6	6	9	12	13	104
Tendinopathy location	No of TA (%)		No of effects (%)			Outcome domain		No of TA (%)		No of effects (%)	
Rotator cuff	77 (45.0)		817 (56.2)			Disability		142 (83.0)		447 (30.7)	
Achilles	45 (26.3)		321 (22.1)			Pain		122 (71.3)		406 (27.9)	
Lateral elbow	29 (17.0)		227 (15.6)								
Patellar	20 (11.7)		89 (6.1)			PFC		59 (34.5)		320 (22.0)	
Dominant therapy class	No of TA (%)		No of effects (%)			ROM		30 (17.5)		159 (10.9)	
Resistance	124 (72.5)		1014 (69.7)			Function		29 (17.0)		68 (4.7)	
Flexibility	25 (14.6)		215 (14.8)								
Proprioception	21 (12.3)		223 (15.3)			QoL		12 (7.0)		54 (3.7)	
Vibration	1 (0.6)		2 (0.1)								

Pain, pain without further specification; Pain time, pain over a specified time; PFC, physical function capacity; QoL, quality of life; ROM, range of motion; TA, treatment arms.

### Description of effect size distributions

From the initial 1454 outcomes extracted, a total of 38 outliers were removed from the analysis with a lower bound threshold of –0.82 (6 effect sizes below) and an upper bound threshold of 7.0 (32 effect sizes above). Across all outcomes and tendinopathy locations, direct calculation of the 0.25 (small), 0.5 (medium) and 0.75 quantiles (large) from the complete empirical data returned the following SMD_pre_ values: 0.37, 0.77 and 1.31, respectively. Application of the meta-analysis model across the data with borrowing of information across studies resulted in similar but shrunken estimates (0.25 quantile_0.5_ = 0.34 (95% CrI: 0.31 to 0.37); 0.5 quantile_0.5_ = 0.73 (95% CrI: 0.70 to 0.77) and 0.75-quantile_0.5_ = 1.21 (95% CrI: 1.17 to 1.27)). A forest plot of effect sizes illustrating effect sizes across studies is presented in online supplemental file 12. Sensitivity analyses checking the potential influences of study type and study quality are presented in online supplemental file 13. No evidence was obtained of greater effect sizes with quasi-experimental designs or with studies identified as high risk of bias.

**Figure 2:**
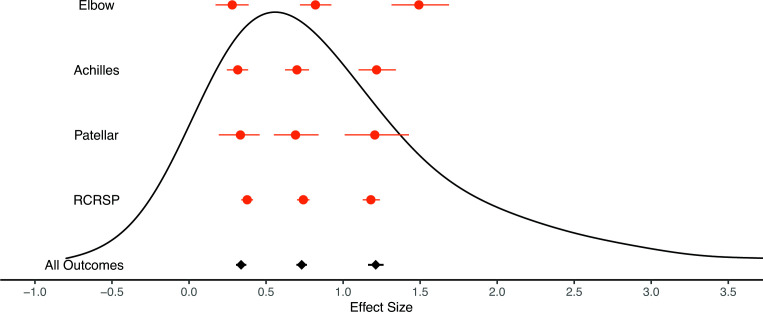
Effect size distributions across tendinopathy locations with identification of small, medium and large thresholds. black curve represents density plot of empirical effect size distribution. Diamonds with intervals represent small, medium and large thresholds with credible intervals (black: all outcomes; red: tendinopathy specific). RCRSP, rotator cuff related shoulder pain.

Analyses of effect size distributions across the different tendinopathy locations are illustrated in figure 2 with numerical values presented in table 3. Analyses were pooled across all outcome domains as a means to compare tendinopathy locations with the largest amount of data possible. Analysis of the modelled small, medium and large thresholds showed considerable overlap in small and medium thresholds across all tendinopathy locations (0.25 quantile_0.5_ ranged from 0.28 to 0.38; 0.5 quantile_0.5_ ranged from 0.70 to 0.82). However, greater divergence was identified for large threshold estimates, with the greatest values estimated for the elbow (0.75 quantile_0.5_ ranged from 1.18 to 1.49).

**Table 3:** Meta-analysis results for all outcomes pooled across different tendinopathy locations

Tendinopathy Location	Small (95%CrI)	Medium (95%CrI)	Large (95%CrI)	Study VPC (75%CrI)	Outcome VPC (75%CrI)	Measurement VPC (75%CrI)
All	0.34 (0.31 to 0.37)	0.73 (0.70 to 0.77)	1.21 (1.17 to 1.27)	0.73 (0.68 to 0.77)	0.27 (0.22 to 0.32)	0.01 (0.00 to 0.02)
RCRSP	0.38 (0.34 to 0.42)	0.74 (0.70 to 0.78)	1.18 (1.13 to 1.24)	0.55 (0.48 to 0.62)	0.45 (0.37 to 0.52)	0.01 (0.00 to 0.02)
Achilles	0.32 (0.25 to 0.38)	0.70 (0.62 to 0.78)	1.22 (1.10 to 1.34)	0.71 (0.59 to 0.81)	0.27 (0.18 to 0.39)	0.01 (0.00 to 0.03)
Elbow	0.28 (0.17 to 0.39)	0.82 (0.72 to 0.92)	1.49 (1.31 to 1.68)	0.90 (0.84 to 0.94)	0.10 (0.06 to 0.15)	0.01 (0.00 to 0.02)
Patellar	0.33 (0.19 to 0.46)	0.69 (0.55 to 0.84)	1.21 (1.00 to 1.43)	0.46 (0.13 to 0.69)	0.52 (0.29 to 0.85)	0.01 (0.00 to 0.05)

Small: 0.25 quantile; medium: 0.5 quantile; large: 0.75 quantile.

CrI, credible interval; RCRSP, rotator cuff related shoulder pain; VPC, variance partition coefficient.

**Figure 3:**
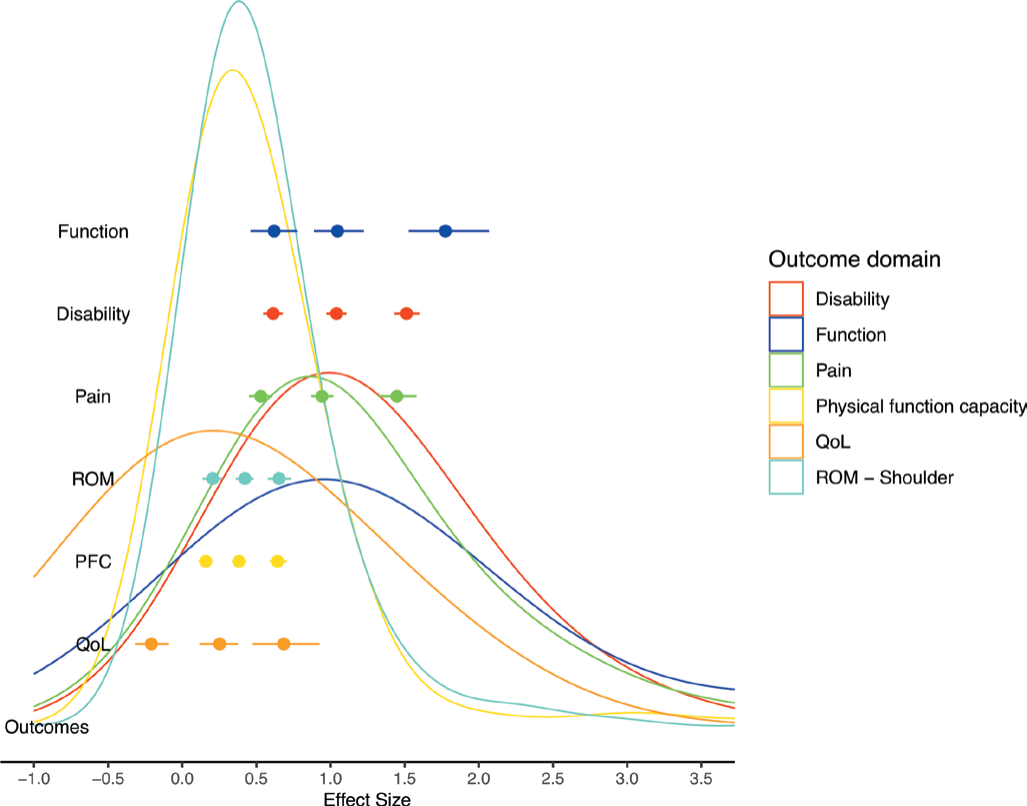
Effect size distributions across outcome domains with identification of small, medium and large thresholds. Each curve represents density plot of empirical effect size distribution for specific outcome domain. Density curves and effect size thresholds are presented in same colours. PFC, physical function capacity; QoL, quality of life; ROM, range of motion.

Analyses of effect size distributions across outcome domains are presented in figure 3 with numerical values presented in table 4. A clear split was identified between the domains of quality of life and the objective measures of PFC and ROM, versus the subjective measures of function, disability and pain. The lowest threshold values were estimated for quality of life, PFC and ROM, with the small threshold for quality of life estimated to be below zero (0.25 quantile_0.5_ = −0.21 (95% CrI: −0.32 to −0.09)). In contrast, the greatest effect sizes values were obtained for outcomes measuring disability, pain and function with the reduced amount of data for function resulting in wider credible intervals. Central estimates indicated that small threshold estimates for domains with the greatest effect sizes were situated between the medium and large threshold estimates for domains with the lowest effect sizes (figure 3).

**Table 4:** Meta-analysis results for all tendinopathy locations pooled across different outcome domains

Outcome domain	Small (95%CrI)	Medium (95%CrI)	Large (95%CrI)	Study VPC (75%CrI)	Outcome VPC (75%CrI)	Measurement VPC (75%CrI)
Function	0.62 (0.45 to 0.77)	1.05 (0.88 to 1.23)	1.78 (1.53 to 2.09)	0.43 (0.03 to 0.81)	0.55 (0.17 to 0.94)	0.01 (0.00 to 0.04)
Disability	0.61 (0.55 to 0.68)	1.04 (0.98 to 1.11)	1.51 (1.43 to 1.60)	0.78 (0.68 to 0.88)	0.15 (0.08 to 0.24)	0.05 (0.00 to 0.13)
Pain	0.53 (0.45 to 0.61)	0.94 (0.87 to 1.02)	1.45 (1.32 to 1.58)	0.26 (0.06 to 0.46)	0.71 (0.51 to 0.90)	0.02 (0.00 to 0.07)
ROM	0.21 (0.14 to 0.27)	0.42 (0.36 to 0.48)	0.65 (0.58 to 0.73)	0.86 (0.76 to 0.93)	0.11 (0.05 to 0.20)	0.01 (0.00 to 0.06)
PFC	0.16 (0.11 to 0.21)	0.38 (0.34 to 0.43)	0.64 (0.59 to 0.71)	0.73 (0.61 to 0.82)	0.27 (0.18 to 0.38)	0.01 (0.00 to 0.02)
QoL	−0.21 (−0.32 to −0.09)	0.25 (0.12 to 0.38)	0.68 (0.48 to 0.93)	0.84 (0.65 to 0.93)	0.14 (0.05 to 0.33)	0.01 (0.00 to 0.04)

Small: 0.25 quantile; medium: 0.5 quantile; large: 0.75 quantile.

CrI, credible interval; PFC, physical function capacity; QoL, quality of life; ROM, range of motion; VPC, variance partition coefficient.

### Moderator analyses

Moderator analyses investigating potential changes in the mean effect size across all outcomes and tendinopathies are presented in table 5. Evidence of a moderator effect was identified for assessment duration (short: ≤12 weeks, medium: 13–52 weeks and long duration: >52 weeks), with results showing a hierarchy and greater mean estimate with increased time from baseline assessment (β_Short:Medium0.5_ = 0.28 (95% CrI: 0.23 to 0.33), p>0.999; β_Short:Long0.5_ = 0.37 (95% CrI: 0.27 to 0.47), p*>*0.999). These estimates remained consistent after meta-regressions were controlled for tendinopathy location and outcome domain (β_Short:Medium0.5_ = 0.28 (95% CrI: 0.23 to 0.33), p>0.999; β_Short:Long0.5_ = 0.38 (95% CrI: 0.27 to 0.48), p>0.999). Consistent evidence of a moderating effect was also obtained for exercise supervision, with a greater mean estimate for supervised exercise therapies (β_Unupervised:Supervised0.5_ = 0.38 (95% CrI: 0.05 to 0.71), p=0.989), which increased in estimate after controlling for tendinopathy location and outcome domain (β_Unupervised:Supervised0.5_ = 0.66 (95% CrI: 0.35 to 0.98), p>0.999). Finally, some evidence was obtained indicating a hierarchy of effects with regards to symptom duration and greater mean estimates with patients reporting shorter symptom durations (β_Medium:Short0.5_ = 0.33 (95% CrI: −0.12 to 0.81), p=0.920; β_Long:Short0.5_ = 0.51 (95% CrI 0.08 to 0.96), p=0.989). However, differences between the symptom duration levels were close to zero after controlling for tendinopathy location and outcome domain (β_Medium:Short0.5_ = −0.05 (95% CrI: -0.44 to 0.36), p=0.411; β_Long:Short0.5_ = 0.06 (95% CrI: −0.34 to 0.47), p=0.619).

**Table 5:** Meta-analysis results for moderator analyses pooling all outcomes across all tendinopathies

Moderator		Estimate of mean (95%CrI)	Probabilities	Study VPC (75%CrI)	Outcome VPC (75%CrI)	Measurement VPC (75%CrI)
Assessment duration	Short (≤12 weeks) 923 outcomes from 144 trials	0.97 (0.85 to 1.1)	*p*(Medium>Short) > 0.999	0.71 (0.66 to 0.75)	0.27 (0.23 to 0.32)	0.02 (0 to 0.04)
	Medium (13–52 weeks) 442 outcomes from 23 trials	1.2 (1.1 to 1.4)	*p*(Long>Medium) = 0.980			
	Long (>52 weeks) 51 outcomes from two trials	1.4 (1.2 to 1.6)	*p*(Long>Short) > 0.999			
Symptom duration	1 year or less 308 outcomes from 44 trials	1.4 (1.1 to 1.7)	*p*(1yr>2yr) = 0.920	0.81 (0.76 to 0.84)	0.17 (0.14 to 0.21)	0.03 (0.00 to 005)
	2 years or less 258 outcomes from 30 trials	1.1 (0.74 to 1.4)	*p*(2yr>+2yr) = 0.772			
	Over 2 years 381 outcomes from 33 trials	0.88 (0.56 to 1.2)	*p*(1yr>+2yr) = 0.989			
Supervision	Supervised 354 outcomes from 35 trials	1.4 (1.1 to 1.7)	*p*(Supervised> Unsupervised) = 0.989	0.77 (0.73 to 0.81)	0.20 (0.17 to 0.24)	0.03 (0.00 to 0.05)
	Unsupervised 914 outcomes from 112 trials	0.99 (0.83 to 1.2)				

Crl, credible interval; p, subjective Bayesian probability; VPC, variance partition coefficient.

## Discussion

The present analysis represents the largest quantitative synthesis of exercise therapy interventions for the management of tendinopathies to date. Data from a total of 114 studies were included, with results demonstrating that substantive improvements in outcomes from baseline are generally obtained. With important clinical relevance, the meta-analyses identified clear differences in the distribution of effects sizes across outcome domains. The greatest values were generally obtained for subjective patient reported outcomes including disability and pain-related outcomes, with considerably lower values obtained for measures of quality of life and objective measures including PFC and ROM. Considerable overlap in effect size distributions were identified across the different tendinopathy location investigated, indicating that similar substantive improvements can be obtained with exercise therapies. Moderator analyses provided consistent evidence of increased improvement in outcomes as time increased from baseline assessment and with supervised compared with non-supervised exercise therapies. Some evidence was obtained for greater improvements with patients reporting symptoms for shorter durations, however, differences did not remain after controlling for outcome domain and tendinopathy location.

Of the 114 studies included in the analysis, 100 (88%) were RCTs and 14 (12%) were quasi-experimental trials. Therapies predominantly focused on resistance exercise, with 70% of outcomes obtained from a treatment arm where this was the dominant class. Tendinopathy has been clinically defined as persistent pain and loss of function related to mechanical loading on a degenerative tendon.^53^ Therefore, resistance exercise that focuses on restoration of loading ability is established as the mainstay of rehabilitation, particularly for lower limb tendinopathies, and is in keeping with current guidance.^54^ Across the included studies flexibility and proprioceptive training were frequently combined with resistance exercise. However, these alternative therapy classes were rarely the dominant exercise class, and where they were, this tended to be restricted to management of RCRSP which accounted for over 50% of outcomes measured in the studies. Given the focus on resistance exercise and the desire to change the mechanical properties of the tendon, the standard duration of exercise therapies featured in the included studies is potentially a major limitation. While clear reporting of therapy duration only occurred in 49% of studies, where it was reported, the median duration was only 8 weeks and over 90% of studies included durations of 12 weeks or shorter. Tendon healing is known to be a complex and lengthy process, with remodelling only beginning 1–2 months postinjury and extending beyond 1 year, suggesting that longer duration exercise interventions are required, which is in keeping with guidelines recommending a minimum 12 weeks duration.^55^ It is also relevant to note that in clinical practice, education and exercise programmes are frequently provided for patients to continue therapy after the initial intervention which may also contribute to the findings observed here including evidence of greater improvements with increased time from baseline assessment.

Review of previous meta-analyses and individual studies investigating the effectiveness of exercise therapy for tendinopathy shows that Cohen’s standard benchmarks are most frequently used to interpret effectiveness.^19–27 56^ The results of the present study show that across most outcomes Cohen’s standard benchmarks of 0.2 (small), 0.5 (medium) and 0.8 (large) would tend to result in an overestimation of effectiveness and for quality of life may result in an underestimation.^28^ Based on the analyses performed in the present meta-analysis, more appropriate thresholds include ~0.4 (small), ~0.8 (medium) and ~1.3 (large), highlighting a systematic shift by one category (eg, what would have been referred to as a medium/large effect is more representative of a small/medium effect). However, the results of the analyses clearly show that effect size distributions are strongly dependent on the outcome domain, with more subjective patient-reported outcomes (eg, pains scales and patient-rated levels of function and disability) producing substantively larger effects compared with more objective assessments (eg, ROM and quantitative measures of PFC). A wide range of outcome measures are used for tendinopathies, with little consensus in the literature to date,^18^ although this should improve in future with work ongoing to identify core outcome sets for specific tendinopathies. International consensus reported that nine outcome domains should be considered core for tendinopathy.^57^ The majority (8/9) of the consensus domains are patient reported, with only PFC being objectively measured in the clinical setting (eg, number of hops/squats, dynamometry). The results of this study highlight that when using patient-centred self-reported outcomes, substantial changes should be expected from most exercise therapies.

The lowest effect sizes were obtained for quality of life outcomes with the small threshold indicating that over 25% of exercise therapies represented by those investigated will result in patients’ reporting a poorer quality of life. However, it is possible that the generic nature of quality of life instruments (eg, EuroQol-5D instrument) insufficiently reflects tendinopathy-specific symptoms or that potential limitations such as ceiling effects limit the usefulness of these instruments. Tendinopathy may be acute but is typically due to chronic overuse and degeneration, exacerbated by overloading; therefore, patients may have developed specific coping strategies over time. Concern was raised when developing core domains that there is no tendon-specific quality of life measure,^57^ but that the overall well-being of patients was important to assess. Further research is required to better understand the factors that influence quality of life assessments when managing tendinopathies and the best measurement tool to use.

Considerable overlap in the effect size thresholds across the five tendinopathies assessed suggests that exercise therapies commonly used to manage the different tendinopathies result in similar profiles of improvement. This contrasts previous perspectives that responses to interventions are variable across tendons and even within tendon sites.^58^ It is likely that the interactions between a single exercise therapy, the tendinopathy and the outcome domain are complex and when better understood can improve patient care. The results of the present analysis, however, indicate that exercise therapies commonly used to manage RCRSP, Achilles, patellar and lateral elbow tendinopathies result in overall similar responses with most causing relatively large changes in relation to baseline SDs. A limitation of the analysis was that it pooled data across all outcome domains to provide sufficient information to compare distributions across the tendinopathies. As the amount of primary data increases, more refined analyses should be conducted to better investigate potential differences across the most common tendinopathies and those which are at present are under researched.

Despite almost all exercise therapies being of short duration (≤12 weeks), a substantial proportion of outcomes were measured beyond the intervention at a medium duration (13–52 weeks), and a relatively small number of instances of longer-term follow-up (>52 weeks). Moderator analysis demonstrated an ordered effect with the smallest mean pooled effect size obtained for short durations (1.0 (95% CrI: 0.87 to 1.1)), and evidence of greater pooled means for medium (1.3 (95% CrI: 1.1 to 1.4); p>0.999) and long durations (1.4 (95% CrI: 1.2 to 1.6) p>0.999). While the absolute magnitude of the differences were relatively small (~0.2 to 0.4 increase) and there were only two studies that included long term observations, the finding are in keeping with previous research,^59^ and provide support for longer duration interventions as well as follow-up periods in studies. Moderator analyses also provided evidence of a greater mean pooled effect with supervised compared with unsupervised exercise therapies (β_Unupervised:Supervised0.5_ = 0.70 (95% CrI: 0.39 to 1.0), p*=*0.797). Previous systematic reviews specifically investigating supervised versus unsupervised exercise therapy for the rotator cuff have suggested that both approaches are likely to lead to similar improvements.^60 61^ While previous reviews employed greater control and focused on more homogeneous comparisons and considerably smaller number of outcomes, the findings in the present analysis and the consistency of results after controlling for tendinopathy location and outcome, demonstrate that in general supervised exercise therapies are likely to provide clinically meaningful improvements beyond unsupervised exercise therapies. Finally, limited evidence was obtained to indicate that greater improvements may be obtained with patients reporting shorter symptom durations. However, differences in pooled mean estimates were close to zero after controlling for tendinopathy location and outcome domains, indicating the need for further research, and highlighting limitations of large modelling studies such as those included here, where systematic differences in a range of potential moderating factors can bias results and generate spurious associations.

Other limitations to consider when interpreting and evaluating the results from the present study include precision of estimates given the reliance on published data and limitations of effect sizes to identify clinical significance. Whist attempts were made to include results from unpublished studies, none met the inclusion criteria and it is known that biases influencing individual studies such as publication bias can result in misleading results in meta-analyses.^62^ Publication bias, however, is most relevant to analyses of pairwise effect sizes, studies employing cohort designs and analyses focusing on null hypothesis testing. When comparing two interventions that are both effective, pairwise comparisons may fail to identify significant differences thereby reducing the chances of dissemination in peer-reviewed journals. In the present study, analyses were conducted on non-controlled effect sizes which are less likely to be influenced by this bias, except in cohort designs that were excluded. Multiple statistical techniques have been developed to correct for potential biases that can skew the results of meta-analyses.^62^ In general, these approaches are best implemented in traditional meta-analyses where the mean effect and confidence intervals are used to make statements regarding a null hypothesis. In contrast, in the present study, inferences focused on describing the majority of the effect size distribution and mitigating biases including potential overestimation of effect sizes through a meta-analysis model that shrunk estimates based on borrowing of strength across studies and accounted for dependencies in the data such that large single values from individual studies had less influence. The use of quantiles to describe distributions and attempts to remove outliers is also likely to have reduced the influence of biases. The importance of removing outliers was highlighted in a previous analysis of SMD effect sizes following exercise interventions based on implausible values due to large underestimations of SD.^49^

In addition to challenges associated with estimates, the present analysis is limited in the ability to address clinical significance. While benchmarking effect sizes using empirical values within a specific context is important,^63^ the labelling of thresholds as small, medium and large remains somewhat arbitrary. In contrast, anchor-based approaches that use an external criterion such as global rating of change are viewed as a superior method to establish important thresholds such as the MIC.^36 64^ Where anchor-based thresholds have not been developed, attempts have been made to use effect sizes such as those presented here (distribution-based approach) as a surrogate. Research, however, has identified a lack of consistency between distribution and anchor-based approaches where it has been argued effect size thresholds should only be used pending development of well-established anchor-based MID values.^36^ Further research is required to establish MIC values for the management of tendinopathy, where the effect sizes reported in the present study may be used to inform values where results from anchor-based approaches diverge.^36^ To assist with clinical interpretations, the threshold values presented can also be transformed into the original scales of measurement.^65^ Using suitable estimates of population means and SDs, effect sizes can be transformed into typical change scores. For example, previous studies of tendinopathy patients have reported baseline Victorian Institute of Sport Assessment-Achilles questionnaire (VISA-A) disability scores of 64±17, and EQ-5D quality of life scores of 0.75±0.15.^66 67^ From table 4, we can compute that a medium effect size for VISA-A scores would reflect an increase from 64 to 64+1.04×17 ≈ 82, and that a small effect size for EQ-5D-5L would reflect a decrease from 0.75 to 0.75-0.21×0.15 ≈ 0.72.

In conclusion, the results from this large meta-analysis show that relative to baseline assessment, a reasonably wide distribution of changes and in general, improvements, should be expected following exercise therapy to manage tendinopathy. The magnitudes of improvement appear somewhat independent of the location of the tendinopathy, but are strongly influenced by the outcome domain, with the greatest improvements measured in subjective patient-reported outcomes (eg, disability, function, pain) and the smallest improvements measured in quality of life and more objective outcomes (eg, PFC and ROM). When interpreting the effectiveness of exercise therapies for the management of tendinopathies, clinicians and researchers should be aware of these factors and can use the context-specific information presented here as a guide. Further research is required to better establish clinical significance using MIC and anchor-based approaches, where the information presented here may assist should divergent results be obtained.

## Data Availability

All data relevant to the study are included in the article or uploaded as online supplemental information.
