# Supplementary material for: What are small, medium and large effect sizes for exercise treatments of tendinopathy? A systematic review and meta-analysis
Source: BMJ Open Sport Exerc Med. 2023 Feb 27;9(1):e001389. doi: 10.1136/bmjsem-2022-001389 (PMC9972446; doi:10.1136/bmjsem-2022-001389)
Supplement: Supplementary data [file bmjsem-2022-001389supp001.pdf]

Supplementary File 1: PRISMA 2020 Checklist and abstract checklist.

Section and Topic	Item #	Checklist item	Location where item is reported
**TITLE**			
Title	1	Identify the report as a systematic review.	1
**ABSTRACT**			
Abstract	2	See the PRISMA 2020 for Abstracts checklist.	SF-1
**INTRODUCTION**			
Rationale	3	Describe the rationale for the review in the context of existing knowledge.	2
Objectives	4	Provide an explicit statement of the objective(s) or question(s) the review addresses.	5
**METHODS**			
Eligibility criteria	5	Specify the inclusion and exclusion criteria for the review and how studies were grouped for the syntheses.	6
Information sources	6	Specify all databases, registers, websites, organisations, reference lists and other sources searched or consulted to identify studies. Specify the date when each source was last searched or consulted.	8
Search strategy	7	Present the full search strategies for all databases, registers and websites, including any filters and limits used.	SF-5
Selection process	8	Specify the methods used to decide whether a study met the inclusion criteria of the review, including how many reviewers screened each record and each report retrieved, whether they worked independently, and if applicable, details of automation tools used in the process.	8
Data collection process	9	Specify the methods used to collect data from reports, including how many reviewers collected data from each report, whether they worked independently, any processes for obtaining or confirming data from study investigators, and if applicable, details of automation tools used in the process.	8
Data items	10a	List and define all outcomes for which data were sought. Specify whether all results that were compatible with each outcome domain in each study were sought (e.g. for all measures, time points, analyses), and if not, the methods used to decide which results to collect.	7
	10b	List and define all other variables for which data were sought (e.g. participant and intervention characteristics, funding sources). Describe any assumptions made about any missing or unclear information.	7
Study risk of bias assessment	11	Specify the methods used to assess risk of bias in the included studies, including details of the tool(s) used, how many reviewers assessed each study and whether they worked independently, and if applicable, details of automation tools used in the process.	9
Effect measures	12	Specify for each outcome the effect measure(s) (e.g. risk ratio, mean difference) used in the synthesis or presentation of results.	12
Synthesis methods	13a	Describe the processes used to decide which studies were eligible for each synthesis (e.g. tabulating the study intervention characteristics and comparing against the planned groups for each synthesis (item #5)).	8
	13b	Describe any methods required to prepare the data for presentation or synthesis, such as handling of missing summary statistics, or data conversions.	9
	13c	Describe any methods used to tabulate or visually display results of individual studies and syntheses.	12
	13d	Describe any methods used to synthesize results and provide a rationale for the choice(s). If meta-analysis was performed, describe the model(s), method(s) to identify the presence and extent of statistical heterogeneity, and software package(s) used.	10
	13e	Describe any methods used to explore possible causes of heterogeneity among study results (e.g. subgroup analysis, meta-regression).	14
	13f	Describe any sensitivity analyses conducted to assess robustness of the synthesized results.	11
Reporting bias assessment	14	Describe any methods used to assess risk of bias due to missing results in a synthesis (arising from reporting biases).	NA
Certainty assessment	15	Describe any methods used to assess certainty (or confidence) in the body of evidence for an outcome.	14
**RESULTS**			

Section and Topic	Item #	Checklist item	Location where item is reported
Study selection	16a	Describe the results of the search and selection process, from the number of records identified in the search to the number of studies included in the review, ideally using a flow diagram.	13
	16b	Cite studies that might appear to meet the inclusion criteria, but which were excluded, and explain why they were excluded.	SF-10
Study characteristics	17	Cite each included study and present its characteristics.	SF-9
Risk of bias in studies	18	Present assessments of risk of bias for each included study.	SF-11
Results of individual studies	19	For all outcomes, present, for each study: (a) summary statistics for each group (where appropriate) and (b) an effect estimate and its precision (e.g. confidence/credible interval), ideally using structured tables or plots.	SF-7
Results of syntheses	20a	For each synthesis, briefly summarise the characteristics and risk of bias among contributing studies.	SF-11
	20b	Present results of all statistical syntheses conducted. If meta-analysis was done, present for each the summary estimate and its precision (e.g. confidence/credible interval) and measures of statistical heterogeneity. If comparing groups, describe the direction of the effect.	12
	20c	Present results of all investigations of possible causes of heterogeneity among study results.	14
	20d	Present results of all sensitivity analyses conducted to assess the robustness of the synthesized results.	SF-13
Reporting biases	21	Present assessments of risk of bias due to missing results (arising from reporting biases) for each synthesis assessed.	NA
Certainty of evidence	22	Present assessments of certainty (or confidence) in the body of evidence for each outcome assessed.	14
**DISCUSSION**			
Discussion	23a	Provide a general interpretation of the results in the context of other evidence.	16
	23b	Discuss any limitations of the evidence included in the review.	16
	23c	Discuss any limitations of the review processes used.	19
	23d	Discuss implications of the results for practice, policy, and future research.	20
**OTHER INFORMATION**			
Registration and protocol	24a	Provide registration information for the review, including register name and registration number, or state that the review was not registered.	6
	24b	Indicate where the review protocol can be accessed, or state that a protocol was not prepared.	6
	24c	Describe and explain any amendments to information provided at registration or in the protocol.	6
Support	25	Describe sources of financial or non-financial support for the review, and the role of the funders or sponsors in the review.	21
Competing interests	26	Declare any competing interests of review authors.	21
Availability of data, code and other materials	27	Report which of the following are publicly available and where they can be found: template data collection forms; data extracted from included studies; data used for all analyses; analytic code; any other materials used in the review.	Supplementary files

From: Page MJ, McKenzie JE, Bossuyt PM, Boutron I, Hoffmann TC, Mulrow CD, et al. The PRISMA 2020 statement: an updated guideline for reporting systematic reviews. BMJ 2021;372:n71. doi: 10.1136/bmj.n71

For more information, visit: http://www.prisma-statement.org/

Section and Topic	Item #	Checklist item	Reported (Yes/No)
**TITLE**			
Title	1	Identify the report as a systematic review.	Yes
**BACKGROUND**			
Objectives	2	Provide an explicit statement of the main objective(s) or question(s) the review addresses.	Yes
**METHODS**			
Eligibility criteria	3	Specify the inclusion and exclusion criteria for the review.	Yes
Information sources	4	Specify the information sources (e.g. databases, registers) used to identify studies and the date when each was last searched.	Yes
Risk of bias	5	Specify the methods used to assess risk of bias in the included studies.	Yes
Synthesis of results	6	Specify the methods used to present and synthesise results.	Yes
**RESULTS**			
Included studies	7	Give the total number of included studies and participants and summarise relevant characteristics of studies.	Yes
Synthesis of results	8	Present results for main outcomes, preferably indicating the number of included studies and participants for each. If meta-analysis was done, report the summary estimate and confidence/credible interval. If comparing groups, indicate the direction of the effect (i.e. which group is favoured).	Yes
**DISCUSSION**			
Limitations of evidence	9	Provide a brief summary of the limitations of the evidence included in the review (e.g. study risk of bias, inconsistency and imprecision).	Yes
Interpretation	10	Provide a general interpretation of the results and important implications.	Yes
**OTHER**			
Funding	11	Specify the primary source of funding for the review.	Yes
Registration	12	Provide the register name and registration number.	Yes

From: Page MJ, McKenzie JE, Bossuyt PM, Boutron I, Hoffmann TC, Mulrow CD, et al. The PRISMA 2020 statement: an updated guideline for reporting systematic reviews. BMJ 2021;372:n71. doi: 10.1136/bmj.n71

For more information, visit: http://www.prisma-statement.org/

Supplementary File 2: Exercise therapy definitions

Therapy Class	Definition	Therapy Treatment	Definition
**Resistance**	Exercise designed primarily to increase strength of muscles by causing them to produce substantive force against an applied resistance which can take several forms including the mass of the body or its segments, isoinertial resistance, elastic resistance, or strength training equipment such as isokinetic devices. In tendinopathy, the stimulus may also be intended to provoke tendon remodelling, reduce pain and improve function.	Concentric Only	Includes movements where force produced overcomes the resistance such that muscle shortening occurs.
		Eccentric Only	Includes movements where force produced is less than the resistance such that controlled muscle lengthening occurs.
		Concentric and eccentric	Includes movements where force produced exceeds the resistance in one phase and is less than the resistance in another such that controlled muscle lengthening and shortening occurs.
		Isokinetic	Uses specialised exercise equipment such that the resistance is adjusted in real-time to ensure joint angular velocity remains constant.
		Isometric	Includes muscular actions against a resistance such that joint angle remains constant.
**Flexibility**	Exercise designed to increase joint range of motion and extensibility of muscles and/or associated	Static	Joint range of motion actions where the movement is held at or near the end range of motion.

Therapy Class	Definition	Therapy Treatment	Definition
	tissues. Also referred to as range-of-motion exercises or stretching.	Dynamic	Joint range of motion actions where the movement is performed continuously into and out of the end range of motion.
		PNF	Proprioceptive neuromuscular facilitation is a technique combining passive stretching and isometric action to achieve maximum range of motion.
		Ballistic	Uses the momentum of a moving body or a limb to increase joint range of motion, bouncing into (or out of) a stretched position.
**Proprioception**	Exercise designed to enhance the sensation of the joint relative to body position and movement, sense of force, and to encourage muscular stabilisation of the joint in the absence of external stabilising devices e.g. ankle brace.	Sense of joint position and force	Exercise aimed at enhancing the ability to perceive joint position and force with minimal external cues.
		Balance	Includes exercise that require the person to keep or return the displacement of centre of gravity over the base of support through various environmental conditions and changes in body position.

Therapy Class	Definition	Therapy Treatment	Definition
		Movement pattern retraining	Exercise aimed at re-education of motor control and movement patterns that may involve specific retraining of under- or over-active muscles and alteration of kinematic rotation +- translation timing between body segments. May also be termed motor control or stabilisation.
**Plyometric**	Exercise where a resistance is overcome by a muscle rapidly stretching then shortening	Plyometric	Exercise where a resistance is overcome by a muscle rapidly stretching then shortening.
**Vibration**	Exercise where body segments are held stationary or actively displaced as per definitions for other treatment classes whilst applying a rapid oscillating resistance	Vibration	Exercise where body segments are held stationary or actively displaced as per definitions for other treatment classes whilst applying a rapid oscillating resistance

Supplementary File 3: Outcome domain definitions and example tools

Domain	ICON Definition	Example Tools
**Disability**	Composite scores of a mix of patient-rated pain & disability due to the pain, usually relating to tendon-specific activities/tasks	VISA scales; DASH; quick DASH; SPADI; Patient-rated tennis-elbow evaluation questionnaire; Constant Murley Score; WORC (Western Ontario Rotator Cuff Index); AOFAS (American Orthopaedic Foot & Ankle Society); Roles and Maudsley score; ASES (American Shoulder & Elbow Surgeons Index; Tegner activity score; Lysholm knee scale; Pain free function questionnaire; Ankle activity score; Subjective elbow Value (SEV); Placzek score; Shoulder disability questionnaire; International Knee Documentation Committee form (IKDC); Penn Shoulder score (university of Pennsylvania shoulder score) (PSS); Brief pain inventory (BPI); UCLA Shoulder Rating Scale; FILLA - functional index of leg and lower limb; Neer Shoulder Score; Nirschl phase rating scale; American Shoulder and Elbow Surgeon's (MASES) questionnaire; Mayo Elbow Performance Score (MEPS); Shoulder rating questionnaire (SRQ)
**Function**:	Participant/patient rated level of function (and not referring to the intensity of their pain; eg, Patient Specific Function Scale on a VAS or NRS).	Patient-specific functional scale
**Pain:** Pain on loading/activity	Patient reported intensity of pain performing a task that loads the tendon	VAS; NRS; Pain experience scale
**Pain:** Pain over a specified time	Patient-reported pain intensity over period of time e.g. morning/night/24-hours/1-week	VAS; NRS Painful days in 3 months
**Pain:** Pain without further specification	Patient asked about pain levels without reference to activity or timeframe	VAS; NRS; Borg CR10 Scale; Pain status
**Physical function capacity**	Quantitative measures of physical tasks (e.g. hops, times walk, single leg squat) includes muscle strength	Counter movement jump; One-leg triple hop; Single-leg decline squat; Muscle strength measured by dynamometry (hand-held, isokinetic); Repetition maximum; Manual muscle testing.
**Quality of Life**	General wellbeing	EQ5D; EQ3D; SF-36 or SF-12; Assessment of Quality of Life (AQoL); Nottingham Health Profile; Gothenburg QoL Instrument
**Range of Motion (Shoulder only)**	Active or passive range of motion in specified plane, measured in degrees.	Hand-held goniometer; inclinometer

Supplementary File 4: Definitions used to define broad and more specific treatment classes for exclusion

**Broad treatment**	**Definition**	**More specific treatment class**	**Definition**
Exercise only	Exercise therapy is defined as a regimen or program of physical activities specifically designed and prescribed to correct impairments, restore musculoskeletal function, and/or maintain a state of wellbeing.	Same as broad treatment class	Same as broad treatment class
Non-active (placebo, sham, wait and see)	Includes any appropriate inactive treatment such as waiting list control, sham shockwave, sham laser, sham taping or true placebo.	Same as broad treatment class	Same as broad treatment class
Non-exercise only	Active treatments used to treat tendinopathy that do not meet the criteria to be considered exercise.	Electrotherapy	Modality that delivers therapeutic levels of physical energy into a biologic system e.g. soft tissue. Includes shockwave, laser and other systems.
		Biomechanics	Treatment using external devices that immobilises (e.g. splinting) or alters the kinematics/kinetics of the limb (e.g. taping, bracing and orthotics).
		Manual-therapy	Manual therapy is the skilled application of “hands-on” techniques to treat soft tissues and joint structures for the purpose of improving pain, increasing range of motion, stimulating tissue repair response, and/or improving function.
		Injection therapy	Injection therapy for tendinopathy typically involves direct

			administration of a pharmacologically active drug, or combination of drugs using a syringe and needle or equivalent. It may or may not be image-guided. Includes Autologous, drug, and volumetric types.
		Surgery	Any relevant surgical intervention for tendinopathy including minimally invasive peritendinous and open intra-tendinous.
Exercise and non-exercise	Treatment comprising multiple components which collectively meet both exercise and non-exercise criteria	Same as broad treatment class	Same as broad treatment class

Supplementary File 5: Search databases and terms

Search last updated 19/01/2021

**Embase (Ovid)**	(exercise OR exercise*.mp OR "isometric exercise" OR kinesiotherapy OR Eccentric.mp OR concentric.mp OR "heavy slow resistance".mp OR "isokinetic exercise" OR plyometrics OR "muscle stretching" OR "muscle training") AND (tendinitis OR Tendinopathy.mp OR "tendon injury" OR "shoulder injury" OR "rotator cuff injury" OR "tennis elbow" OR tendin.mp OR tendon.mp OR bursitis OR "shoulder impingement syndrome" OR 2posterior tibial tendon dysfunction" OR "Greater trochanteric pain syndrome".mp)
**CINAHL (EBSCO-host)**	(MH Exercise OR AB exercise* OR MH "muscle strengthening" OR MH "rehabilitation" OR MH "eccentric contraction" OR TX "heavy slow resistance exercis*" OR AB eccentric OR AB concentric OR AB isokinetic OR MH "therapeutic exercise") AND (MH tendinopathy OR MH "arm injuries" OR "tendon injuries" OR MH tendons OR TX tendin* OR TX tendon* OR AB bursitis OR MH Bursitis OR MH "Posterior tibial tendon dysfunction" OR MH "shoulder impingement syndrome" OR AB "Greater trochanteric pain syndrome")
**Medline (EBSCO-host)**	(MH exercise OR AB exercise* OR MH "isometric contraction" OR MH rehabilitation OR TX eccentric OR TX concentric OR TX "heavy slow resistance" OR TX isokinetic) AND (MH tendinopathy OR MH "shoulder injuries" OR MH tendons OR MH "tendon injuries OR TX tendin* OR tendon* OR MH bursitis OR AB bursitis OR MH "posterior tibial tendon dysfunction" OR MH "shoulder impingement syndrome" OR AB "greater trochanteric pain syndrome")
**SPORTDiscus (EBSCO-host)**	(DE exercise OR DE "exercise therapy" OR AB exercise* OR TX eccentric OR TX concentric OR TX "heavy slow resistance" OR DE "isokinetic exercise" OR DE plyometrics OR DE "strength training" OR DE "stretch (physiology)" OR DE "isometric exercise" OR DE rehabilitation) AND (DE tendinitis OR DE tendinosis OR AB tendinopathy OR DE "tendon injuries" OR "shoulder injuries" OR DE "tennis elbow" OR AB tendin* OR AB tendon* OR DE bursitis OR AB "shoulder impingement syndrome" OR AB "posterior tibial tendon dysfunction" OR AB "greater trochanteric pain syndrome")
**Amed (EBSCO-host)**	(ZU exercise OR ZU "exercise therapy" OR AB exercise OR ZU "muscle stretching exercises" OR ZU "isometric contraction" OR ZU rehabilitation OR TZ eccentric OR TZ concentric OR TX "heavy slow resistance" OR TX isokinetic OR AB plyometric) AND (ZU tendinopathy OR ZU "tendon injuries" OR ZU tendons OR ZU "shoulder injuries" OR ZU "tennis elbow" OR TX tendin* OR TX tendon* OR ZU bursitis OR AB bursitis OR ZU "shoulder impingement syndrome" OR ZU "posterior tibial tendon dysfunction" OR AB "greater trochanteric pain syndrome")
**Open Grey**	Tendinopathy AND exercise<br>Tendin* AND exercise<br>Tendon AND exercise
**Mednar**	Tendinopathy AND exercise<br>Tendin* AND exercise<br>Tendon AND exercise
**New York Academy Grey Literature Report**	Tendinopathy AND exercise<br>Tendin* AND exercise<br>Tendon AND exercise
**EThOS**	Tendinopathy AND exercise<br>Tendin* AND exercise<br>Tendon AND exercise

**Google Scholar**	Tendinopathy AND exercise<br>Tendin* AND exercise<br>Tendon AND exercise
**JBI Evidence Synthesis**	Tendinopathy AND exercise
**Cochrane Library**	Tendinopathy AND exercise<br>Tendin* AND exercise<br>Tendon AND exercise
**PEDro**	Tendinopathy AND exercise<br>Tendin* AND exercise<br>Tendon AND exercise
**Epistemonikos**	(tendinopathy OR tendon* OR tendin*) AND exercise
**CORE**	Tendinopathy AND exercise<br>Tendin* AND exercise<br>Tendon AND exercise
**Clinicialtrials.gov**	Tendinopathy AND exercise<br>Tendin* AND exercise<br>Tendon AND exercise
**ISRCTN**	Tendinopathy AND exercise<br>Tendin* AND exercise<br>Tendon AND exercise
**EU CTR**	Tendinopathy AND exercise<br>Tendin* AND exercise<br>Tendon AND exercise
**ANZCTR**	Tendinopathy AND exercise<br>Tendin* AND exercise<br>Tendon AND exercise

ISRCTN – the Research Registry; EU CTN – European Clinical Trials Registry; ANZCTR – Australia and New Zealand Clinical Trials Registry

Supplementary File 6: Extraction codebook

	Column	Heading	Description
Study Details	A	Initials Reviewer	Identification of individual extracting information
	B	Covidence Identifier	Reference number for Covidence
	C	Author	First author surname *et al.,*
	D	Year	Year of publication
	E	Title	Study title
	F	Country	Country where study was conducted
	G	Journal	Journal name
	H	Aims/Purpose	Study aims/purpose
	I	Tendinopathy type	1=Achilles; 2= Lateral elbow (tennis); 3 = Patellar; 4 = Rotator cuff (SI)
	J	Study Design	RCT = 1; Quasi-experimental = 2
	K	Age Mean	Mean age of study sample as a whole
	L	Age SD	Standard deviation age of study sample as a whole
	M	Baseline Total N	Total sample across all interventions measured at baseline
	N	Training Status Description	Brief description of training status of study sample as a whole
	O	Training Status Code	1 = Performance; 2 = Sporting; 3 = Other
	P	Sex	Percentage female of study sample as a whole
	Q	BMI Mean	Mean BMI of study sample as a whole
	R	BMI SD	Standard deviation of BMI of study sample as a whole
	S	Symptom Severity Mean	Mean severity measure at baseline of study sample as a whole
	T	Symptom Severity SD	Standard deviation of severity measure at baseline of study sample as a whole
	U	Symptom Duration Mean (Months)	Mean symptom duration reported in months
	V	Symptom Duration SD (Months)	Standard deviation symptom duration reported in months
	W	Population Comments	Any additional information relevant to the participants investigated including diagnostic criteria
Outcomes	X	Outcome Category	1 = Disability; 2 = Pain on loading/activity; 3 = Pain over a specified time; 4 = Pain without further specification; 5 = Physical function capacity
	Y	Outcome Tool	Description of outcome tool
	Z	Reflection	1 = Increase in outcome indicates positive treatment; -1 = Decrease in outcome indicates positive treatment
	AA	Measurement Time (Weeks)	Time of measurement in weeks
Interventi	AB	Dominant Treatment Class	**Only one dominant theme to be selected** 1 = Resistance; 2 = Plyometric; 3 = Vibration; 4 = Flexibility; 5 = Movement pattern retraining

	AC	Total Treatment class	**Multiple themes to be selected as required** 1 = Resistance; 2 = Plyometric; 3 = Vibration; 4 = Flexibility; 5 = Movement pattern retraining
	AD	Intervention N	Intervention sample size at specified time
	AE	Intervention Total Duration	Total duration of exercise intervention in weeks
	AF	Intervention Adherence %	Reporting of adherence to exercise (reported as a percentage) if applicable
	AG	Intervention Location	Location exercise was performed 1 = Home; 2 = Clinic; 3 = Fitness facility; 4 = NR; 5 = NA
	AH	Intervention Volume	Numerical value describing volume
	AI	Intervention Volume Category	1 = Duration of session (mins); 2 = sets * repetitions; 3 = number of repetitions; 4 = number of sets
	AJ	Intervention Volume Comments	Any additional information relevant.
	AK	Intervention Intensity	Numerical value describing intensity
	AL	Intervention Intensity Category	1 = Absolute; 2 = Relative
	AM	Intervention Frequency	Number of sessions per week. Where there is progression, average value is to be entered.
	AN	Intervention Frequency Comments	Any additional information relevant.
	AO	Intervention Progression	**Multiple themes to be selected as required** 1 = No progression; 2 = NR; 3 = Progression volume; 4 = Progression intensity; 5 = Progression frequency; 6 = Progression specificity; 7 = Progression capacity; 8 = Other
	AP	Intervention Progression Comments	Any additional information relevant.
**Control**	AQ	Control Comparator	1 = Placebo; 2 = No treatment
	AR	Control Comparator Comments	Any additional information relevant.
**Data**	AS	Intervention Baseline Mean	Baseline mean for exercise therapy
	AT	Intervention Baseline SD	Baseline standard deviation for exercise therapy
	AU	Intervention Measurement Mean	Mean of outcome for exercise therapy at stated time point
	A	Intervention	Standard deviation of outcome for exercise therapy at

V	Measurement SD	stated time point
AW	Control Baseline Mean	Baseline mean for control
AX	Control Baseline SD	Baseline standard deviation for control
AY	Control Measurement Mean	Mean of outcome for control at stated time point
AZ	Control Measurement SD	Standard deviation of outcome for control at stated time point
BA	Measurement Comments	State if a different value has been entered for means (e.g. median), a different value for standard deviations (e.g. standard error, IQR, percentiles, distance from mean to upper bound). Provide the relevant statistic (width of CI's, width of percentiles). Also state if data has been extracted by digitization

*** Outcome Specific**

Supplementary file 8: Analysis R Code

Variables:

1) NT: Number of treatment arms
2) ID: Study ID according to included list
3) Study Type: Binary RCT or quasi-experimental
4) Tendinopathy.Type: Category variable of the tendinopathy locations
5) Outcome.Domain: Category variable of the outcome domain
6) Outcome.Tool: Description of the outcome measure fitting within the outcome domain
7) ExerciseHierarchy: Binary classification of exercise therapy as exercise only or multiple exercise types
8) ClassD: Category variable of dominant exercise class in therapy
9) ClassAll: Category variable of all exercise classes in therapy
10) TreatmentD: Category variable of dominant exercise treatment in therapy
11) TreatmentAll: Category variable of all exercise treatments in therapy
12) N: Number of participants data are collected from
13) AgeMean: Mean age of participants
14) AgeSD: Standard deviation of age of participants
15) BMIMean: Mean BMI of participants
16) BMISD: Standard deviation of BMI of participants
17) Supervision: Category variable of whether exercise therapy was supervised or unsupervised
18) Location: Category variable of location exercise therapy was performed
19) Random.sequence.generation: Risk of bias variable
20) Allocation.concealment: Risk of bias variable
21) Blinding.of.participants.personnel: Risk of bias variable
22) Blinding.of.outcome.assessment: Risk of bias variable
23) Incomplete.outcome.bias: Risk of bias variable
24) Selective.reporting: Risk of bias variable
25) Other.bias: Risk of bias variable
26) Time: Time in weeks of measurement from baseline
27) TimeC: Category variable identifying time in weeks of measurement from baseline
28) OutcomeLevel: Variable for nesting structure at outcome level
29) MesureLevel: Variable for nesting structure at measurement level
30) ES: Standardised mean difference effect size
31) SEES: Standard error of standardised mean difference effect size

NA: refers to missing data

Four level model with t-distribution: (All Outcomes)

```
Mod0ALL.prior = get_prior(ES | se(SEES,sigma=TRUE) ~ 1 +
(1|ID/OutcomeLevel/MeasureLevel), family = student(), data=Data)
Mod0ALL.prior$prior[10] = "student_t(3, 0, 1.5)"
set.seed(123)
mod0ALL = brm(ES | se(SEES,sigma=TRUE) ~ 1 + (1|ID/OutcomeLevel/MeasureLevel), family =
student(),
```

```
        data = Data, prior = Mod0ALL.prior, chains = 4, iter = 20000, warmup = 10000)
mod0ALLPS= posterior_samples(mod0ALL)

# Quantile function
QuantileFunction = function(data,Model,PosteriorDF){
 Preds =  data[c('ES','SEES','ID','OutcomeLevel','MeasureLevel')] %>%
  add_predicted_draws(Model)
 PredsDF = as.data.frame(Preds)

 PredsL = length(data[,1])
 PredsFitL = length(PosteriorDF[,1])

 PredsDF2 = matrix(data = NA, nrow=PredsL,ncol=PredsFitL+1)
 PredsDF2[,1] =
  PredsDF[(seq(1,PredsL*PredsFitL,
          PredsFitL)),6]
 for(i in 1:PredsFitL){
  PredsDF2[,(i+1)] =
   PredsDF[(seq(1,PredsL*PredsFitL,
           PredsFitL)+(i-1)),10]
 }

 q025 = c(NULL)
 for(i in 1:PredsFitL){
  q025[i] = quantile(PredsDF2[,i+1],0.25)[[1]]
 }

 q025q = quantile(q025,c(0.025,0.5,0.975))

 q05 = c(NULL)
 for(i in 1:PredsFitL){
  q05[i] = quantile(PredsDF2[,i+1],0.5)[[1]]
 }

 q05q = quantile(q05,c(0.025,0.5,0.975))

 q075 = c(NULL)
 for(i in 1:PredsFitL){
  q075[i] = quantile(PredsDF2[,i+1],0.75)[[1]]
 }

 q075q = quantile(q075,c(0.025,0.5,0.975))

 return(rbind(q025q,q05q,q075q))
}

mod0ALLQ = QuantileFunction(Data, mod0ALL, mod0ALLPS)

# Repeat for different Outcome
```

```
mod0Outcome = brm(ES | se(SEES,sigma=TRUE) ~ 1 + (1|ID/OutcomeLevel/MeasureLevel),
family = student(),
        data = Data[Data$Outcome.Domain==”Outcome”,], prior = mod0Outcome.prior, chains = 4,
iter = 20000, warmup = 10000)
mod0OutcomePS= posterior_samples(mod0Outcome)

modOutcomeQ = QuantileFunction(Data[Data$Outcome.Domain==”Outcome”,], mod0Outcome,
mod0OutcomePS)

# Plot
ggplot(Data,
      aes(x=ES)) + geom_density(adjust = 2) +
  annotate("text",x=-0.68, y=0, label="All Outcomes", size=3) +
  coord_cartesian(xlim = c(-1, 3.5), ylim =c(0,0.7), expand = TRUE) +
  theme_classic() + theme(axis.line.y = element_blank()) + ylab("") +
  theme(axis.text.y = element_blank()) + theme(axis.ticks.y = element_blank()) +
  scale_x_continuous("Effect Size", breaks = seq(-1,3.5,0.5)) +
  theme(axis.text.x = element_text(size=8)) +  theme(axis.title.x = element_text(size=9)) +
  annotate("pointrange", y=0, x =mod0N4ALLQ[[1,2]],xmin = mod0N4ALLQ[[1,1]], xmax =
mod0N4ALLQ[[1,3]],colour = "black", size = 0.6, shape = 18) +
  annotate("pointrange", y=0, x =mod0N4ALLQ[[2,2]],xmin = mod0N4ALLQ[[2,1]], xmax =
mod0N4ALLQ[[2,3]],colour = "black", size = 0.6, shape = 18) +
  annotate("pointrange", y=0, x =mod0N4ALLQ[[3,2]],xmin = mod0N4ALLQ[[3,1]], xmax =
mod0N4ALLQ[[3,3]],colour = "black", size = 0.6, shape = 18) +
  annotate("text",x=-0.6, y=0.5, label="Disability", size=3) +
  annotate("pointrange", y=0.5, x =mod0N4DISQ[[1,2]],xmin = mod0N4DISQ[[1,1]], xmax =
mod0N4DISQ[[1,3]],colour = "red", size = 0.4) +
  annotate("pointrange", y=0.5, x =mod0N4DISQ[[2,2]],xmin = mod0N4DISQ[[2,1]], xmax =
mod0N4DISQ[[2,3]],colour = "red", size = 0.4) +
  annotate("pointrange", y=0.5, x =mod0N4DISQ[[3,2]],xmin = mod0N4DISQ[[3,1]], xmax =
mod0N4DISQ[[3,3]],colour = "red", size = 0.4) +
  annotate("text",x=-0.6, y=0.6, label="Function", size=3) +
  annotate("pointrange", y=0.6, x =mod0N4FUNQ[[1,2]],xmin = mod0N4FUNQ[[1,1]], xmax =
mod0N4FUNQ[[1,3]],colour = "red", size = 0.4) +
  annotate("pointrange", y=0.6, x =mod0N4FUNQ[[2,2]],xmin = mod0N4FUNQ[[2,1]], xmax =
mod0N4FUNQ[[2,3]],colour = "red", size = 0.4) +
  annotate("pointrange", y=0.6, x =mod0N4FUNQ[[3,2]],xmin = mod0N4FUNQ[[3,1]], xmax =
mod0N4FUNQ[[3,3]],colour = "red", size = 0.4) +
  annotate("text",x=-0.6, y=0.4, label="Pain", size=3) +
  annotate("pointrange", y=0.4, x =mod0N4PAINQ[[1,2]],xmin = mod0N4PAINQ[[1,1]], xmax =
mod0N4PAINQ[[1,3]],colour = "red", size = 0.4) +
  annotate("pointrange", y=0.4, x =mod0N4PAINQ[[2,2]],xmin = mod0N4PAINQ[[2,1]], xmax =
mod0N4PAINQ[[2,3]],colour = "red", size = 0.4) +
  annotate("pointrange", y=0.4, x =mod0N4PAINQ[[3,2]],xmin = mod0N4PAINQ[[3,1]], xmax =
mod0N4PAINQ[[3,3]],colour = "red", size = 0.4) +
  annotate("text",x=-0.6, y=0.2, label="PFC", size=3) +
  annotate("pointrange", y=0.2, x =mod0N4PFCQ[[1,2]],xmin = mod0N4PFCQ[[1,1]], xmax =
mod0N4PFCQ[[1,3]],colour = "red", size = 0.4) +
```

```
  annotate("pointrange", y=0.2, x =mod0N4PFCQ[[2,2]],xmin = mod0N4PFCQ[[2,1]], xmax =
mod0N4PFCQ[[2,3]],colour = "red", size = 0.4) +
  annotate("pointrange", y=0.2, x =mod0N4PFCQ[[3,2]],xmin = mod0N4PFCQ[[3,1]], xmax =
mod0N4PFCQ[[3,3]],colour = "red", size = 0.4) +
  annotate("text",x=-0.6, y=0.1, label="QoL", size=3) +
  annotate("pointrange", y=0.1, x =mod0N4QOLQ[[1,2]],xmin = mod0N4QOLQ[[1,1]], xmax =
mod0N4QOLQ[[1,3]],colour = "red", size = 0.4) +
  annotate("pointrange", y=0.1, x =mod0N4QOLQ[[2,2]],xmin = mod0N4QOLQ[[2,1]], xmax =
mod0N4QOLQ[[2,3]],colour = "red", size = 0.4) +
  annotate("pointrange", y=0.1, x =mod0N4QOLQ[[3,2]],xmin = mod0N4QOLQ[[3,1]], xmax =
mod0N4QOLQ[[3,3]],colour = "red", size = 0.4) +
  annotate("text",x=-0.6, y=0.3, label="ROM", size=3) +
  annotate("pointrange", y=0.3, x =mod0N4ROMQ[[1,2]],xmin = mod0N4ROMQ[[1,1]], xmax =
mod0N4ROMQ[[1,3]],colour = "red", size = 0.4) +
  annotate("pointrange", y=0.3, x =mod0N4ROMQ[[2,2]],xmin = mod0N4ROMQ[[2,1]], xmax =
mod0N4ROMQ[[2,3]],colour = "red", size = 0.4) +
  annotate("pointrange", y=0.3, x =mod0N4ROMQ[[3,2]],xmin = mod0N4ROMQ[[3,1]], xmax =
mod0N4ROMQ[[3,3]],colour = "red", size = 0.4)
```

Supplementary File 9: Table of included studies and reference list

Study (first author, year, country)	Design	Tendinopathy Location	Participants (number (n); sex (%female); mean (sd) age; mean (sd) symptom duration in months)	Exercise Treatment arms	Exercise Treatment classes	Findings
Aceituno-Gómez 2019 Spain[1]	Quasi-experimental	Rotator cuff - subacromial impingement	N=43 % female 60.9 Age 59 (8.9) Symptoms NR Training status Other	1	(Flexibility, Resistance)	High-intensity laser therapy plus exercise did not give greater improvements in pain and functionality in patients with subacromial syndrome than exercise alone.
Akkaya 2016 Turkey[2]	RCT	Rotator cuff - subacromial impingement	N=34 % female 67.6 Age 41.7 (8.9) Symptoms 6.9 (4.1) Training status Other	2	2*(Flexibility)	Weighted and un-weighted solo pendulum exercises achieved significant clinical improvements but showed no differences in ultrasonographic acrohumeral distance measurements between groups.
Alfredson 1998 Sweden[3]	Quasi-experimental	Achilles	N= 30 % female 20.0 Age 44.0 (7.0) Symptoms 25.9 (3-100)** Training status Recreational	1	(Resistance)	Our treatment model with heavy-load eccentric calf muscle training has a very good short-term effect on athletes in their early forties.
Alfredson 1999 Sweden[4]	Quasi-experimental	Achilles	N= 24 % female 14.3 Age 42.6 (9.0) Symptoms 23.7 (3-100)** Training status Recreational	1	(Resistance)	Heavy-loaded, eccentric calf-muscle training seems to be a good treatment mode for chronic Achilles tendinosis.
Arias-Buría 2017 Spain[5]	RCT	Rotator cuff - subacromial impingement	N= 50 % female 26.0 Age 48.5 (5.5) Symptoms 71.9 (21.6) Training status Other	1	(Resistance)	This study found that the inclusion of 2 sessions of TrP-DN into an exercise program was effective for improving shoulder pain-related disability at short-, medium-, and long-term; however, no greater improvement in shoulder pain was observed.
Arias--Buría 2015 Spain[6]	RCT	Rotator cuff - subacromial impingement	N= 36 % female 75.0	1	(Resistance)	Ultrasound-guided percutaneous electrolysis combined with eccentric

			Age 57.5 (6.4) Symptoms 10.9 (2.6) Training status Other			exercises resulted in better short-term outcomes compared to eccentric exercises alone.
Bae 2011 Korea (Republic of)[7]	Quasi-experimental	Rotator cuff - subacromial impingement	N=35 % female 65.7 Age 49.1 (4.9) Symptoms Training status Other	1	(Proprioception, Resistance)	The motor control and strengthening programme improved pain, function, strength and ROM.
Bahr 2006 Norway[8]	RCT	Patellar	N= 40 % female 12.5 Age 30.5 (7.9) Symptoms 34 (28.7) Training status Other	1	(Resistance)	No added benefit was observed for surgical treatment to eccentric strength training. Eccentric training should be offered for 12 weeks before tenotomy is considered for the treatment of patellar tendinopathy.
Balius 2016 Spain[9]	RCT	Achilles	N=37 % female 20.4 Age 41.4 (11.7) Symptoms NR Training status Other	6	4*(Resistance);2*(Flexibility)	Findings confirmed the therapeutic potential of eccentric exercise at reactive and degenerative stages of tendinopathy. MCVC supplementation decreased pain more than eccentric exercise alone (reactive tendinopathy) Personalized stretching regime supplemented with MCVC may be appropriate for some patients
Bang 2000 United States[10]	RCT	Rotator cuff - subacromial impingement	N=50 % female 42.3 Age 43 (9.1) Symptoms 5 (3.3) Training status Other	1	(Flexibility, Resistance)	Manual physical therapy applied by experienced physical therapists combined with supervised exercise in a brief clinical trial is better than exercise alone for increasing strength, decreasing pain, and improving function in patients with shoulder impingement syndrome
Başkurt 2011 Turkey[11]	Quasi-experimental	Rotator cuff - subacromial impingement	N= 40 % female 67.5 Age 51.4 (10.0) Symptoms NR Training status Other	2	1*(Flexibility, Resistance);1*(Flexibility, Proprioception)	Scapular stabilisation combine with stretching and strengthening exercises can be more effective in the short-term for SIS.
Beyer 2015 Denmark[12]	RCT	Achilles	N= 58 % female 31.9 Age 48.0 (2.0) Symptoms 18.1 (4.3) Training	2	2*(Resistance)	Both traditional eccentric exercise and HSR yield positive, equally good and lasting clinical results in patients with Achilles tendinopathy. HSR is associated with greater patient satisfaction after 12

			status Other			weeks but not after 52 weeks.
Blume 2015 United States[13]	RCT	Rotator cuff - subacromial impingement	N= 34 % female 58.0 Age 49.4 (15.6) Symptoms 22.7 (24.3) Training status Other	2	2*(Flexibility, Resistance)	Both eccentric and concentric PRE programs resulted in improved function, AROM, and strength in patients with SAIS. However, no difference was found between the two exercise modes, suggesting that therapists may use exercises that utilize either exercise mode in their treatment of SAIS.
Boudreau 2019 Canada[14]	RCT	Rotator cuff - subacromial impingement	N= 42 % female 52.4 Age 42.9 (12.0) Symptoms 43.0 (46.6) Training status Other	2	2*(Resistance)	No additional benefit was found to adding coactivation to regular rotator cuff strengthening exercises at 6-weeks.
Breda 2020 Netherlands[15]	RCT	Patellar	N= 76 % female 23.7 Age 24 (3.9) Symptoms 98.5 (NR) Training status Performance	2	1*(Plyometric, Resistance);1*(Flexibility, Resistance)	In patients with patellar tendinopathy, progressive tendon-loading exercises resulted in a significantly better clinical outcome after 24 weeks than eccentric exercise therapy. Progressive tendon-loading exercises are superior to eccentric exercise therapy and are therefore recommended as initial conservative treatment for patellar tendinopathy.
Brox 1999 Norway[16]	RCT	Rotator cuff - subacromial impingement	N= 125 % female 44.0 Age 47.6 (23-66)** Symptoms NR Training status Other	1	(Proprioception, Resistance)	At 2.5 years follow-up, both arthroscopic surgery and supervised exercises are better treatments than placebo with no significant difference between the 2 active treatments.
Calis 2011 Turkey[17]	RCT	Rotator cuff - subacromial impingement	N= 52 % female 67.3 Age 49.2 (12.6) Symptoms 3.0 (1-24)** Training status Other	1	(Flexibility)	Ultrasound and laser treatments were not superior to each other in the treatment of SIS
Chaconas 2017 United States[18]	RCT	Rotator cuff - subacromial impingement	N=46 % female 41.7 Age 45.9 (17.4) Symptoms 49.1 (80) Training status Other	2	2*(Flexibility, Resistance)	An eccentric program targeting the external rotators was superior to a general exercise program for strength, pain, and function after six months. The findings suggest eccentric training may be efficacious to improve self-

						report function and strength for those with SAPS.
Cheng 2007 Hong Kong, China (SAR)[19]	RCT	Rotator cuff - subacromial impingement	N=94 % female Age 32.4 (10.2) Symptoms 23.4 Training status Other	2	1*(Flexibility, Proprioception);1*(Resistance, Flexibility)	An eccentric program targeting the external rotators was superior to a general exercise program for strength, pain, and function after six months. The findings suggest eccentric training may be efficacious to improve self-report function and strength for those with subacromial pain syndrome.
Cho 2017 Korea (Republic of)[20]	Quasi-experimental	Patellar	N= 30 % female 46.7 Age 33.1 (29.1) Symptoms 15.1 (16.1) Training status Other	1	(Flexibility, Proprioception, Resistance)	A rehabilitation exercise programme was more effective at improving pain, strength and function in patellar tendinopathy that injection therapy alone.
de Jonge 2008 Netherlands[21]	RCT	Achilles	N= 70 % female NR Age 44.6 (26-59) ** Symptoms 30.7 (2-204) ** Training status Other	1	(Resistance)	Eccentric exercises with or without a night splint improved functional outcome at one year follow-up. At follow-up there was no significant difference in clinical outcome when a night splint was used in addition to an eccentric exercise.
de Oliveira 2020 Canada[22]	RCT	Rotator cuff - subacromial impingement	N= 52 % female 42.3 Age 30.2 (8.3) Symptoms 22.6 (26.7) Training status Other	1	(Flexibility, Proprioception, Resistance)	Whereas symptoms, functional limitations, ROM, and AHD improved in both groups, the addition of KT did not lead to superior outcomes compared with exercise-based treatment alone, in the mid and long term, for individuals with RCRSP.
de Vos 2007 Netherlands[23]	RCT	Achilles	N= 63 % female 41.3 Age 44.6 (8) Symptoms 30.6 (50.6) Training status Recreational	1	(Resistance)	A night splint has no added benefit to eccentric exercises in the treatment of chronic midportion Achilles tendinopathy. There was no significant difference between the two groups in VISA-A score and patient satisfaction.
Dejaco 2017 Netherlands[24]	RCT	Rotator cuff - subacromial impingement	N=36 % female 47.3 Age 49.5 (11.3) Symptoms 19.7 (20.1) Training status Other	2	2*(Flexibility, Resistance)	12-week-isolated eccentric training programme of the RC is beneficial for shoulder function and pain after 26 weeks in patients with RC tendinopathy. However, it is no more beneficial than a conventional exercise programme for the RC and scapular muscles.
Devereaux 2016 Canada[25]	RCT	Rotator cuff - subacromial impingement	N= 100 % female 37.9	2	1*(Flexibility, Proprioception);1*(Resistance, Flexibility)	The improvements in pain and function observed with an NSAID or precut

			Age 48.0 (11.9) Symptoms NR Training status Other			kinesiology tape as adjuvant treatments were no greater than with rehabilitation exercise alone.
Dimitrios 2013 Cyprus[26]	Quasi-experimental	Patellar	N= 60 % female 36.7 Age 47.57 (5.9) Symptoms 4.5 (NR) Training status Other	2	2*(Flexibility, Resistance)	A specific supervised exercise programme is superior to a specific home exercise programme in reducing pain and improving function in patients with LET at the end of the treatment and at the 3 month follow-up.
Dimitrios 2012 Greece[27]	Quasi-experimental	Patellar	N= 60 % female 36.7 Age 47.6 (5.9) Symptoms 4.5 (NR) Training status Other	2	1*(Flexibility, Resistance);1*(Resistance)	Eccentric training and static stretching exercises is superior to eccentric training alone to reduce pain and improve function in patients with patellar tendinopathy at the end of the treatment and at follow-up.
Dupuis 2018 Canada[28]	RCT	Rotator cuff - subacromial impingement	N=43 % female 55.8 Age 33.3 (11.7) Symptoms 0.9 (0.3) Training status Other	2	1*(Flexibility);1*(Flexibility, Resistance)	Both groups showed statistically significant improvements on symptoms and function at 2 weeks and 6 weeks but there was no difference between the short-term effect of cryotherapy and a gradual reloading exercise programme.
Engebretsen 2009 Norway[29]	RCT	Rotator cuff - subacromial impingement	N= 104 % female 50.0 Age 48.0 (10.6) Symptoms 12.5 (NR) Training status Other	1	(Plyometric, Proprioception, Resistance)	Supervised exercises are superior to ESWT in terms of shoulder pain, disability and some work-related outcomes.
Engebretsen 2011 Norway[30]	RCT	Rotator cuff - subacromial impingement	N= 104 % female 50.0 Age 48.0 (10.6) Symptoms 12.5 (NR) Training status Other	1	(Proprioception)	Both radial ESWT and the supervised exercise regime devised by Bohmer (1998) provided similar benefits in pain and function-related outcomes. However, exercise may be superior for work-related outcomes.
Gatz 2020 Germany[31]	RCT	Achilles	N= 42 % female 35.7 Age 50.0 (12.0) Symptoms 27.5 (23.8) Training status Other	2	2*(Resistance)	No additional clinical benefits of adding ISOs to a basic EE program could be found in this preliminary randomized controlled trial study over a period of 3 months. SWE was able to differentiate between insertional and midportion tendon tissue and localize reported symptoms to sublocations but this did not correlate with better clinical scores (VISA-A) over a 3-

						month follow-up period.
Giray 2019 Turkey[32]	RCT	Lateral elbow/tennis elbow	N= 30 % female 86.7 Age 44.46 (9.92) Symptoms 1.69 (NR) Training status Other	1	(Flexibility, Resistance)	Kinesiotaping in addition to exercises is more effective than sham taping and exercises alone in improving pain in daily activities and arm disability due to lateral epicondylitis.
Granviken 2015 Norway[33]	RCT	Rotator cuff - subacromial impingement	N=44 % female 48 Age 47.9 (9.9) Symptoms 14.5 Training status Other	2	1*(Flexibility, Proprioception);1*(Resistance, Flexibility)	No significant differences in pain and disability were found between home exercises and supervised exercises of more than the first session of a 6-week exercise regime for people with subacromial impingement.
Hallgren 2014 Sweden[34]	RCT	Rotator cuff - subacromial impingement	N= 50 % female 37.0 Age 52 (30-65)** Symptoms 18 (6-186)* Training status Other	2	1*(Resistance);1*(Flexibility)	Specific exercises produced positive short-term improvements at 1-year follow-up and reduces the need for surgery. Full-thickness tear and a low CMS score appear to be predictors of poor outcome.
Hallgren 2017 Sweden[35]	RCT	Rotator cuff - subacromial impingement	N= 108 % female 34.1 Age 58 (NR) Symptoms NR Training status Other	2	1*(Flexibility, Resistance);1*(Flexibility)	More patients in the specific exercise group managed to avoid surgery compared to the unspecific exercise group at 5-year follow-up supporting it's prescription as an initial treatment for patients with subacromial pain.
Heron 2017 United Kingdom[36]	RCT	Rotator cuff - subacromial impingement	N= 120 % female 41.0 Age 49.9 (NR) Symptoms NR Training status Other	3	2*(Flexibility, Resistance);1*(Flexibility)	Open chain, closed chain, and range of movement exercises all seem to be effective in bringing about short-term changes in pain and disability in patients with rotator cuff tendinopathy.
Hotta 2020 Brazil[37]	RCT	Rotator cuff - subacromial impingement	N=60 % female 70 Age 49 (9) Symptoms 28.5 (24) Training status Other	2	1*(Resistance, Proprioception);1*(Resistance)	The inclusion of the isolated scapular stabilization exercises, emphasizing retraction and depression of the scapula, to a progressive general periscapular strengthening protocol did not add benefits to self-reported shoulder pain and disability, muscle strength, and ROM in patients with subacromial pain syndrome.
Johansson 2005 Sweden[38]	RCT	Rotator cuff - subacromial impingement	N=85 % female 69.4 Age 49 (7.5) Symptoms NR Training	1	(Flexibility, Resistance)	Acupuncture was more effective than ultrasound when applied in addition to home exercises.

			status Other			
Johnson 2005 Sweden[39]	RCT	Patellar	N= 15 % female 13.3 Age 24.9 (8.2) Symptoms 17.5 (13.2) Training status Performance	2	2*(Resistance)	Eccentric, but not concentric, quadriceps training on a decline board, seems to reduce pain in jumper's knee.
Jonsson 2009 Sweden[40]	Quasi-experimental	Achilles	N= 15 % female 13.3 Age 25.0 (NR) Symptoms 17.5 (13.2) Training status Other	2	2*(Resistance)	Treatment with painful eccentric calf-muscle training showed good clinical results based on VAS scores, patient satisfaction, and return to pre-injury activity levels in patients with chronic painful mid-portion Achilles tendinosis, but not in patients with chronic insertional Achilles tendon pain.
Juul-Kristensen 2019 Denmark[41]	RCT	Rotator cuff - subacromial impingement	N= 58 % female 51 Age 42.9 (12.4) Symptoms NR Training status Other	2	2*(Proprioception, Flexibility)	Electromyography-biofeedback neuromuscular shoulder exercises and neuromuscular shoulder exercises were both effective in reducing pain to a clinically relevant level, while electromyography biofeedback did not make a difference. The current neuromuscular shoulder exercise protocol is recommended
Ketola 2009 Finland[42]	RCT	Rotator cuff - subacromial impingement	N=134 % female 62.9 Age 47.1(23.3-60.0)** Symptoms 2.6 (NR) Training status Other	1	(Resistance, Proprioception)	Arthroscopic acromioplasty provides no clinically important effects over a structured and supervised exercise programme alone in terms of subjective outcome or cost-effectiveness when measured at 24 months.
Ketola 2013 Finland[43]	RCT	Rotator cuff - subacromial impingement	N=140 % female 62.9 Age 41.7 Symptoms Training status Other	1	(Flexibility, Resistance)	Differences in the patient-centred primary and secondary parameters between the two treatment groups were not statistically significant, suggesting that acromioplasty is not cost-effective.
Kim 2017 Korea (Republic of)[44]	RCT	Rotator cuff - subacromial impingement	N= 40 % female 72.5 Age 51.1 (10.6) Symptoms NR Training status Other	2	2*(Proprioception))	The use of visual feedback and 3D motion images can improve pain and function in SIS.
Kim	RCT	Rotator cuff -	N= 40	1	(Proprioception, Vibration)	Both the Neurac modality

2020 Korea (Republic of)[45]		subacromial impingement	% female 100 Age 46.2 (4.6) Symptoms NR Training status Other			and manual therapy induced pain relief, improved function, and increased ROM. The Neurac intervention also resulted in a significant enhancement of shoulder muscle strength indicating its superiority as an effective therapeutic modality for this particular patient group.
Knobloch 2008 Italy[46]	RCT	Achilles	N= 92 % female 35.0 Age 47.5 (11.0) Symptoms NR Training status Recreational	1	(Resistance)	Patients with tendinopathy of the main body of the AT experienced improved clinical outcome with both management options. Although tendon microcirculation was optimized in the combined group of eccentric training and AirHeel Brace, these micro-vascular advantages do not translate into superior clinical performance when compared with eccentric training alone.
Knobloch 2007 Germany[47]	RCT	Achilles	N= 20 % female 45.0 Age 32.5 (11.0) Symptoms NR Training status	1	(Resistance)	An eccentric-training program performed daily over 12 weeks reduced the increased paratendinous capillary blood flow in Achilles tendinopathy by as much as 45% and decreased pain level based on a visual analog scale. Local paratendon oxygenation was preserved while paratendinous postcapillary venous filling pressures were reduced after 12 weeks of eccentric training, which appears to be beneficial from the perspective of microcirculation.
Knobloch 2007 Germany[48]	RCT	Achilles	N= 118 % female 40 Age 48.5 (12) Symptoms NR Training status Other	1	(Resistance)	Achilles tendon oxygen saturation is increased, and capillary venous clearance facilitated using an Achilles wrap in addition to daily 12-week eccentric training
Kongsgaard 2009 Denmark[49]	RCT	Patellar	N= 37 % female 0 Age 32.4 (8.8) Symptoms 18.7 (12.3) Training status Recreational	2	2*(Resistance)	Corticosteroid injection has good short-term but poor long-term clinical effects, in patellar tendinopathy. Heavy-slow resistance exercise has good short- and long-term clinical effects accompanied by pathology improvement and increased collagen turnover.
Kromer 2014	RCT	Rotator cuff - subacromial	N= 90 % female	1	(Flexibility, Proprioception, Resistance)	The use of MT including Physiotherapy provides no

Germany[50]		impingement	51.1<br>Age 51.8 (11.2)<br>Symptoms 24.1 (35.1)<br>Training status Other			additional benefits and is more expensive in comparison to exercise only interventions.
Kromer<br>2013<br>Germany[51]	RCT	Rotator cuff - subacromial impingement	N= 90<br>% female 51.1<br>Age 51.8 (11.2)<br>Symptoms 7.8 (9.8)<br>Training status Other	1	(Flexibility, Proprioception, Resistance)	Individually adapted exercises were effective in the treatment of patients with shoulder impingement syndrome. Individualized manual Physiotherapy contributed only a minor amount to the improvement in pain intensity.
Littlewood<br>2016<br>United Kingdom[52]	RCT	Rotator cuff - subacromial impingement	N= 60<br>% female 50.3<br>Age 54.7 (NR)<br>Symptoms 14.6 (NR)<br>Training status Other	1	(Resistance)	Self-management programme based on a single exercise were comparable to usual Physiotherapy in the short-, mid- and long-term.
Ludewig<br>2003<br>United States[53]	RCT	Rotator cuff - subacromial impingement	N= 85<br>% female 0.0<br>Age 48.8 (2.1)<br>Symptoms NR<br>Training status Other	1	(Flexibility, Resistance)	Home exercise programme are more effective in reducing symptoms and improving function (Shoulder Rating Questionnaire, shoulder satisfaction score) than the control group in construction workers with shoulder pain.
Luginbuhl<br>2008<br>Switzerland[54]	RCT	Lateral elbow/tennis elbow	N= 30<br>% female 72.7<br>Age 47 (9)<br>Symptoms 10 (11)<br>Training status Other	1	(Resistance)	No beneficial effect of neither the forearm support band nor the strengthening exercises could be found.
Maenhout<br>2013<br>Belgium[55]	RCT	Rotator cuff - subacromial impingement	N= 61<br>% female 59.0<br>Age 39.8 (13.0)<br>Symptoms NR<br>Training status Other	2	2*(Resistance)	Adding heavy load eccentric training resulted in a higher gain in isometric strength at 90 degree of scapular abduction but was not superior for decreasing pain and improving shoulder function. The addition of a limited amount of Physiotherapy sessions combined with a daily home exercise programme is highly effective in patients with impingement.
Mafi<br>2001<br>Sweden[56]	RCT	Achilles	N= 44<br>% female 45.5<br>Age 48.3 (8.8)<br>Symptoms 20.5 (3-	2	2*(Resistance)	Eccentric calf muscle training showed superior results to concentric training in the treatment of chronic Achilles tendinosis based on patient satisfaction and return to activity level.

			120)** Training status Other			
Manias 2006 United Kingdom[57]	RCT	Lateral elbow/tennis elbow	N= 40 % female 67.5 Age 42.86 (6.23) Symptoms NR Training status Other	2	2*(Resistance)	An exercise programme consisting of eccentric and static stretching exercises had reduced the pain in patients with lateral epicondyle tendinopathy at the end of the treatment and at the follow up whether or not ice was included.
Martinez-Silvestrini 2005 United States[58]	Quasi-experimental	Lateral elbow/tennis elbow	N= 81 % female 46.8 Age 45.5 (7.7) Symptoms NR Training status Other	3	2*(Flexibility, Resistance);1*(Flexibility)	Eccentric strengthening for the wrist extensors in subjects with lateral epicondylitis demonstrated improvement at six weeks but was not statistically different from that achieved with a conservative program with stretching or a concentric strengthening program.
Marzetti 2014 Italy[59]	RCT	Rotator cuff - subacromial impingement	N= 48 % female 61.4 Age 62.1 (12.5) Symptoms NR Training status Other	2	1*(Flexibility, Resistance);1*(Proprioception)	Neurocognitive rehabilitation is effective in reducing pain and improving function in patients with shoulder impingement syndrome, with benefits maintained for at least 24 weeks.
McCormack 2016 United States[60]	RCT	Achilles	N= 15 % female 68.8 Age 53.6 (38-69)** Symptoms 9.9 (NR) Training status Other	1	(Resistance)	Soft tissue treatment (Astym) plus eccentric exercise was more effective than eccentric exercise alone at improving function during both short- (26 weeks) and long-term (52 weeks) follow-up periods.
Melegati 2000 Italy[61]	RCT	Rotator cuff - subacromial impingement	N= 90 % female 65.5 Age 54.4 (3.0) Symptoms NR Training status Other	1	(Flexibility, Resistance)	Groups A (kinesitherapy) and B (ESWT + kinesitherapy) achieved a significant constant score improvement, whereas the increase in group C (control) was not significant.
Mulligan 2016 United States[62]	RCT	Rotator cuff - subacromial impingement	N=50 % female 65 Age 50.1 (10.7) Symptoms 7.9 (7.4) Training status Other	1	(Proprioception, Resistance)	Patients with SAIS demonstrate improvement in pain and function with a standardized program of physical therapy regardless of group exercise sequencing.
Nishizuka 2017 Japan[63]	RCT	Lateral elbow/tennis elbow	N=110 % female 39.1 Age 53.6 (11.8) Symptoms 2.04 (1.77)	1	(Flexibility)	A forearm band may have no more than a placebo effect and is not recommended based on its effectiveness.

			Training status Other			
Nørregaard 2007 Denmark[64]	RCT	Achilles	N= 35 % female 49.0 Age 42.0 (2.0)*** Symptoms 28.4 (8.8)*** Training status Other	2	1*(Resistance);1*(Flexibility)	Symptoms gradually improved during the 1-year follow-up period and were significantly better assessed by pain and symptoms after 3 weeks and all later visits. However, no significant differences could be observed between the two groups.
Nowotny 2018 Germany[65]	RCT	Lateral elbow/tennis elbow	N= 31 % female 57 Age 46 (NR) Symptoms NR Training status Other	1	(Resistance)	The use of an elbow orthosis appears to reduce pain and improve other subjective outcome measures. However, the long-term results do not appear to be any greater than those received through Physiotherapy alone.
Østerås 2010 Norway[66]	RCT	Rotator cuff - subacromial impingement	N=61 % female 20.5 Age 43.9 (13) Symptoms 40.2 (56.3) Training status Other	2	2*(Flexibility, Resistance)	In long-term subacromial pain syndrome, high dosage medical exercise therapy is superior to a conventional low dosage exercise programme
Paavola 2018 Finland[67]	RCT	Rotator cuff - subacromial impingement	N= 186 % female 69.8 Age 50.6 (5.0) Symptoms 19.5 (18.9) Training status NR	1	(Flexibility, Proprioception, Resistance)	Arthroscopic subacromial decompression provided no benefit over diagnostic arthroscopy in patients with shoulder impingement syndrome.
Park 2010 Korea (Republic of)[68]	RCT	Lateral elbow/tennis elbow	N=31 % female 61.3 Age 50.2 (34-63)** Symptoms 6.3 (2-17)** Training status NR	1	(Resistance)	Isometric strengthening exercises done early in the course of LE (within 4 weeks) provides a clinically significant improvement.
Pearson 2012 New Zealand[69]	RCT	Patellar	N= 40 % female 62.5 Age 50.0 (8.2) Symptoms 11.0 (10.0) Training status Other	1	(Resistance)	There is some evidence for small short-term symptomatic improvements with the addition of autologous blood injection to standard treatment for Achilles tendinopathy.
Pearson 2018 Australia[70]	RCT	Achilles	N= 16 % female 0 Age 28 (4.25) Symptoms 34.17 (1.95) Training	2	2*(Resistance)	Pain was significantly reduced after isometric loading on both SLDS and hop tests. Pain and quadriceps function improved over the 4 weeks. Short-duration isometric

			status Performance			contractions are found to be as effective as longer duration contractions for relieving patellar tendon pain when total time under tension is equalized.
Pekyavas 2016 Turkey[71]	RCT	Rotator cuff - subacromial impingement	N=70 % female NR Age 47.1 (13.8) Symptoms NR Training status Other	1	(Flexibility, Resistance)	HILT and MT were found to be more effective in reducing pain and disability and improving ROM in patient with SAIS.
Petersen 2007 Germany[72]	RCT	Achilles	N= 86 % female 40.0 Age 42.5 (11.1) Symptoms 7.4 (2.3) Training status Recreational	1	(Resistance)	The AirHeel brace is as effective as eccentric training in the treatment of chronic Achilles tendinopathy. There is no added benefit to combining both treatments.
Peterson 2011 Sweden[73]	RCT	Lateral elbow/tennis elbow	N= 81 % female 42 Age 48.25 (8.35) Symptoms 23.3 (35.9) Training status Other	2	2*(Resistance)	Exercise appears to be superior to the control group in reducing pain in chronic lateral epicondylosis.
Peterson 2014 Sweden[74]	RCT	Lateral elbow/tennis elbow	N= 120 % female 47.5 Age 47.9 (8.1) Symptoms NR Training status Other	1	(Resistance)	Eccentric graded exercise reduced pain and increased muscle strength in chronic tennis elbow more effectively than concentric graded exercise at follow-up. However, there were no significant differences in function or quality of life measures between the two groups.
Polimeni 2003 Italy[75]	RCT	Rotator cuff - subacromial impingement	N= 50 % female 72.0 Age 56 (16) Symptoms NR Training status Other	1	(Flexibility)	All patients experienced improvement with treatment, but the association of physical therapy and functional rehabilitation did not seem to lead to added benefit for the patient.
Praet 2019 Australia[76]	RCT	Achilles	N= 20 % female 35.0 Age 43.7 (7.9) Symptoms 54 (90) Training status Recreational	1	(Resistance)	Oral supplementation of specific collagen peptides may accelerate the clinical benefits of a well-structured calf-strengthening and return-to-running programme in patients with chronic Achilles tendinopathy.
Rabusin 2020 Australia[77]	RCT	Achilles	N= 100 % female 52.0	1	(Resistance)	In adults with mid-portion Achilles tendinopathy, heel lifts were more effective

			Age 45.85 (9.4) Symptoms 20.25 (NR) Training status Other			than calf muscle eccentric exercise in reducing pain and improving function at 12 weeks.
Reyhan 2020 Turkey[78]	RCT	Lateral elbow/tennis elbow	N= 40 % female 82.5 Age 42.4 (9.9) Symptoms 4 (0.78) Training status Other	1	(Flexibility, Resistance)	MWM plus exercise and cold therapy is safe and effective at improving elbow pain, functional capacity, and grip strength.
Rio 2017 Australia[79]	RCT	Patellar	N= 20 % female 10.0 Age 22.5 (4.7) Symptoms NR Training status Performance	2	2*(Resistance)	Both isometric and isotonic contraction protocols appear efficacious for in-season athletes to reduce pain, however, isometric contractions demonstrated significantly greater immediate analgesia throughout the 4-week trial.
Romero-Morales 2020 Spain[80]	RCT	Achilles	N= 61 % female 26 Age 41.6 (8.7) Symptoms 4.25 (3.5) Training status Other	2	1*(Resistance, Vibration);1*(Resistance)	Authors encourage the use of vibration with respect to cryotherapy added to eccentric exercise programs in order to enhance multifidus cross-sectional area in addition to lower limb functionality in individuals who suffer from chronic non-insertional AT.
Rompe 2007 Germany[81]	RCT	Achilles	N= 75 % female 61.3 Age 48.5 (10.6) Symptoms 10.8 (8.5) Training status Other	1	(Flexibility, Resistance)	At 4-month follow-up, eccentric loading and low-energy shock-wave therapy showed comparable results. The wait-and-see strategy was ineffective for the management of chronic recalcitrant Achilles tendinopathy.
Rompe 2009 Germany[82]	RCT	Achilles	N= 68 % female 55.9 Age 49.7 (9.9) Symptoms 14.5 (6.0) Training status Other	1	(Resistance)	The likelihood of recovery after 4 months was higher after a combined approach of both eccentric loading and shock-wave therapy compared to eccentric loading alone.
Rompe 2008 Germany[83]	RCT	Achilles	N= 50 % female 60.0 Age 39.8 (11) Symptoms 25.55 (9.45) Training status Other	1	(Resistance)	Eccentric loading as applied in the present study showed inferior results to low-energy shock wave therapy as applied in patients with chronic recalcitrant tendinopathy of the insertion of the Achilles tendon at four months follow-up.

Roos 2004 Sweden[84]	RCT	Achilles	N= 44 % female 52.3 Age 45 (26-60)** Symptoms 5.5 (1-180)* Training status Recreational	1	(Resistance)	Eccentric exercises reduce pain and improve function in patients with Achilles tendinopathy.
Şenbursa 2011 Turkey[85]	RCT	Rotator cuff - subacromial impingement	N= 47 % female NR Age 49.0 (9.3) Symptoms NR Training status Other	2	2*(Flexibility, Resistance)	Supervised exercise, supervised and MT, and home-based exercise are all effective and promising treatments for patients with subacromial impingement syndrome. The addition of an initial MT may improve outcomes with exercise.
Seven 2017 Turkey[86]	RCT	Rotator cuff - subacromial impingement	N= 101 % female 45.5 Age 48.5 (11.6) Symptoms 19.5 (12.4) Training status Other	1	(Proprioception)	Prolotherapy is an easily applicable treatment which may be superior in enhancing pain and function outcomes in comparison to exercise alone.
Sevier 2015 United States[87]	RCT	Lateral elbow/tennis elbow	N= 90 % female 57.9 Age 46.95 (6.55) Symptoms NR Training status Other	1	(Flexibility, Resistance)	Astym therapy is an effective treatment option for patients with LE tendinopathy, as an initial treatment, and after an eccentric exercise program has failed.
Silbernagel 2007 Sweden[88]	RCT	Achilles	N= 38 % female 47.4 Age 46.0 (8.0) Symptoms 36.2 (66.5) Training status Other	2	1*(Flexibility, Plyometric, Resistance);1*(Flexibility, Plyometric)	Our treatment protocol which gradually increases the load on the Achilles tendon and calf muscle, demonstrated significant improvements. Continuing tendon loading activity such as running and jumping with the use of a pain-monitoring model did not have any adverse effect.
Silbernagel 2001 Sweden[89]	RCT	Achilles	N= 47 % female 22.5 Age 44.0 (12.5) Symptoms 30.5 (40.7) Training status Recreational	2	1*(Flexibility, Proprioception, Resistance);1*(Flexibility)	The eccentric overload protocol used in the present study can be recommended for patients with chronic pain from the Achilles tendon. More patients achieved full recovery, improved pain and ROM in the Exp group compared to the control group.
Şimşek, 2013 Turkey[90]	RCT	Rotator cuff - subacromial impingement	N= 38 % female 65.8 Age 51.0 (18-69)** Symptoms NR	1	(Proprioception, Resistance)	Findings were inconclusive and require further research.

			Training status Other			
Stasinopoulos 2017 Cyprus[91]	RCT	Lateral elbow/tennis elbow	N= 34 % female 55.8 Age 43.7 (4.6) Symptoms 6 (NR) Training status Recreational	3	2*(Flexibility, Resistance);1*(Resistance)	Eccentric training, eccentric-concentric training, and eccentric-concentric training combined with isometric contraction reduced pain and improved function at the end of the treatment and follow-up. The eccentric-concentric training combined with isometric contraction produced the largest effect at the end of the treatment and follow-up.
Stasinopoulos 2006 Greece[92]	Quasi-experimental	Lateral elbow/tennis elbow	N= 75 % female 38.6% Age 40.3 (5.8) Symptoms 5 (NR) Training status Other	1	(Flexibility, Resistance)	Cyriax Physiotherapy, a supervised exercise programme, and polarized polychromatic non-coherent light reduced pain and improved function at the end of the treatment and at any of the follow-up time points. The supervised exercise programme produced the largest effect in the short, intermediate and long term.
Stasinopoulos 2010 Greece[93]	Quasi-experimental	Lateral elbow/tennis elbow	N= 70 % female 52.9 Age 45.1 (5.8) Symptoms 5 (NR) Training status NR	2	2*(Flexibility, Resistance)	Supervised exercise programme is superior to home exercise programme to reduce pain and improve function in patients with LET at the end of the treatment and at the follow-up.
Stefansson 2019 Iceland[94]	RCT	Achilles	N= 58 % female 20.0 Age NR Symptoms NR Training status Other	1	(Resistance)	Similar results for pressure massage and eccentric exercise. Combining pressure massage and eccentric exercise did not improve outcomes
Steunebrink 2013 Netherlands[95]	RCT	Patellar	N= 33 % female 24.2 Age 32.9 (10) Symptoms 11 (8) Training status Recreational	1	(Resistance)	Continuous topical GTN treatment in addition to an eccentric exercise programme does not improve clinical outcome compared to placebo patches and an eccentric exercise programme in patients with chronic patellar tendinopathy.
Stevens 2014 United Kingdom[96]	RCT	Achilles	N= 28 % female 60.7 Age 48.7 (10.8) Symptoms 7.4 (4.0) Training status Other	2	2*(Resistance)	Performing a 6-week do-as-tolerated program of eccentric heel-drop exercises compared to the recommended 180 repetitions per day, did not lead to lesser improvement for individuals with midportion Achilles

						tendinopathy, based on VISA-A and VAS scores.
Svernlov 2001 Sweden[97]	Quasi-experimental	Lateral elbow/tennis elbow	N= 57 % female 61.3 Age 50.15 (NR) Symptoms 6.3 (NR) Training status Other	1	(Flexibility, Resistance)	Significant improvements observed for VAS and grip strength warrants clinical use of this regime.
Tahran 2020 Turkey[98]	RCT	Rotator cuff - subacromial impingement	N= 67 % female 30.5 Age 52.9 (11.0) Symptoms NR Training status Other	2	2*(Flexibility)	All treatments improved pain, shoulder mobility, function, and disability in patients with SIS. However, modified posterior shoulder stretching exercises in addition to a treatment program was superior to the treatment program alone in improving pain with activity, internal rotation ROM, and dysfunction. Moreover, stretching provided clinically significant improvements.
Tonks 2007 United Kingdom[99]	RCT	Lateral elbow/tennis elbow	N= 34 % female NR Age 44.3 (7.1) Symptoms NR Training status Other	1	(Flexibility, Resistance)	Patients who received steroid injection were statistically significantly better for all outcome measures at follow up. No statistically significant effect of Physiotherapy nor interaction between Physiotherapy and injection was found.
Turgut 2017 Turkey[100]	RCT	Rotator cuff - subacromial impingement	N= 30 % female 46.7 Age 36.45 (17.5) Symptoms 6.28 (5.4) Training status Other	2	1*(Flexibility, Proprioception, Resistance);1*(Flexibility)	Progressive exercise training independent from specific scapular stabilization exercises provides decreased disability and pain severity in impingement syndrome. All groups showed improvement, however, there were no significant differences between the groups.
Vallés-Carrascosa 2018 Spain[101]	RCT	Rotator cuff - subacromial impingement	N= 22 % female 54 Age 59.0 (58.5-70.0)* Symptoms Training status Other	2	2*(Flexibility, Resistance)	Both rotator cuff eccentric exercise protocols with scapular stabilising and stretching of upper trapezius were equally effective in improving pain, function, and active ROM in the short-term in patients with subacromial syndrome.
vanArk 2016 Australia[102]	RCT	Patellar	N= 19 % female 6.9 Age 23 (4.7) Symptoms 35.8 (33.8) Training status Recreational	2	2*(Resistance)	This study found favourable results for athletes with patellar tendinopathy without modification of the training. Both isometric and isotonic exercise programs reduced pain and improve function in athletes with patellar tendinopathy during a season.

Vinuesa-Montoya 2017 Spain[103]	RCT	Rotator cuff - subacromial impingement	N= 40 % female 26.8 Age 47.0 (9.0) Symptoms 6.2 (3.8) Training status Other	1	(Flexibility, Resistance)	Cervicothoracic manipulative treatment with mobilisation plus exercise therapy may improve intensity of pain and ROM compared with home exercise alone.
Visnes 2005 Norway[104]	RCT	Patellar	N= 29 % female 38.5 Age 26.58 (NR) Symptoms 73.6 (62.3) Training status Performance	1	(Resistance)	There was no effect on knee function (VISA) from a 12-week program with eccentric training among a group of volleyball players with patellar tendinopathy who continued to train and compete during the treatment period. Whether the training would be effective if the patients did not participate in sports activity is not known.
Vuvan 2019 Australia[105]	RCT	Lateral elbow/tennis elbow	N= 39 % female 28 Age 48.5 (9) Symptoms 4 (NR) Training status Other	2	2*(Flexibility, Resistance)	Unsupervised isometric exercise was effective in improving pain and disability, but not perceived rating of change and pain-free grip strength when compared with wait-and-see at 8 wk. With only one of the three primary outcomes being significantly improved, it is doubtful if isometric exercises can be an efficacious standalone treatment.
Walther 2004 Germany[106]	RCT	Rotator cuff - subacromial impingement	N= 60 % female 43.3 Age 50.7 (NR) Symptoms 27.3 (NR) Training status Other	2	1*(Flexibility, Resistance);1*(Flexibility, Proprioception)	There were no statistically significant differences among the groups. Guided self-training can lead to results similar to those of conventional Physiotherapy.
Wegener 2016 Australia[107]	RCT	Lateral elbow/tennis elbow	N= 40 % female 70 Age 49.52 (8.09) Symptoms NR Training status NR	1	(Flexibility, Resistance)	Whilst all groups improved on key outcomes, it is possible that exercise alone and/or natural recovery were responsible for improvements.
Wen 2011 United States[108]	RCT	Lateral elbow/tennis elbow	N= 28 % female 46.4 Age 46 (7.3) Symptoms 3.3 (2.2) Training status Other	1	(Resistance)	The authors were unable to show any statistical advantage to eccentric exercises for lateral epicondylosis compared with local modalities and stretching exercises.
Werner 2002 Germany[109]	RCT	Rotator cuff - subacromial impingement	N=20 % female 50 Age 51.75	2	1*(Flexibility, Resistance);1*(Proprioception, Resistance)	Strengthening of the centering muscles around the humeral head lead to

			(NR) Symptoms 27.5 Training status Other			positive outcomes for subacromial impingement. Self-training after instruction showed no difference to physiotherapist-supervised exercises.
Wiedmann 2017 Germany[110]	RCT	Achilles	N= 20 % female 65.0 Age 43.0 (6.0) Symptoms NR Training status Other	1	(Resistance)	Eccentric training improved the VISA-A and VAS scores after 12 weeks more than Physiotherapy treatment.
Yelland 2011 Australia[111]	RCT	Achilles	N= 43 % female NR Age 46.7 (NR) Symptoms 17 (NR) Training status Other	1	(Resistance)	Prolotherapy and particularly eccentric loading exercises combined with prolotherapy gave more rapid improvements in Achilles tendinosis symptoms than eccentric loading exercises alone. Long term VISA-A scores were similar.
Yerlikaya 2018 Turkey[112]	Quasi-experimental	Lateral elbow/tennis elbow	N= 90 % female 71.1 Age 48.6 (8.8) Symptoms NR Training status Other	1	(Flexibility, Resistance)	Lateral epicondylitis does not seem to be affected by either leukocyte-rich-PRP or leukocyte-poor-PRP on pain and function in the short term.
Young 2005 Australia[113]	RCT	Patellar	N= 17 % female 23.5 Age 27.3 (1.8) Symptoms NR Training status Performance	2	2*(Resistance)	Both exercise protocols improved pain and sporting function in volleyball players over 12 months. The decline squat protocol offers greater clinical gains during a rehabilitation programme for patellar tendinopathy in athletes who continue to train and play with pain.
Yu 2013 Korea (Republic of)[114]	Quasi-experimental	Achilles	N= 32 % female 0.0 Age 30.3 (1.6) Symptoms 11.7 (2.1) Training status Other	2	1*(Resistance, Flexibility)1*(Resistance)	Eccentric strengthening was more effective than concentric strengthening in reducing pain and improving function in patients with Achilles tendinopathy.

Reference list of included studies

1 Aceituno-Gómez J, Avendaño-Coy J, Gómez-Soriano J, et al. Efficacy of high-intensity laser therapy in subacromial impingement syndrome: a three-month follow-up controlled clinical trial. *Clin Rehabil* 2019;33:894-903.

2 Akkaya N, Akkaya S, Gungor HR, et al. Effects of weighted and un-weighted pendulum exercises on ultrasonographic acromiohumeral distance in patients with subacromial impingement syndrome. *J Back Musculoskelet Rehabil* 2017;30:221-228.

3 Alfredson H, Pietilä T, Jonsson P, et al. Heavy-load eccentric calf muscle training for the treatment of chronic Achilles tendinosis. *Am J Sports Med* 1998;26:360-366.

4 Alfredson H, Nordström P, Pietilä T, et al. Bone mass in the calcaneus after heavy loaded eccentric calf-muscle training in recreational athletes with chronic achilles tendinosis. *Calcif Tissue Int* 1999;64:450-455.

5 Arias-Buría JL, Fernández-de-Las-Peñas C, Palacios-Ceña M, et al. Exercises and dry needling for subacromial pain syndrome: A randomized parallel-group trial. *J Pain* 2017;18:11-18.

6 Arias-Buría JL, Truyols-Domínguez S, Valero-Alcaide R, et al. Ultrasound-guided percutaneous electrolysis and eccentric exercises for subacromial pain syndrome: a randomized clinical trial. *Evidence-Based Complementary and Alternative Medicine* 2015;315219-9.

7 Bae YH, Lee GC, Shin WS, et al. Effect of motor control and strengthening exercises on pain, function, strength and the range of motion of patients with shoulder impingement, syndrome. *Journal of Physical Therapy Science* 2011;23:687-92.

8 Bahr R, Fossan B, Løken S, et al. Surgical treatment compared with eccentric training for patellar tendinopathy (jumper's knee): a randomized, controlled trial. *JBJS* 2006;88:1689-1698.

9 Balius R, Álvarez G, Baró F, et al. A 3-arm randomized trial for Achilles tendinopathy: eccentric training, eccentric training plus a dietary supplement containing mucopolysaccharides, or passive stretching plus a dietary supplement containing mucopolysaccharides. *Current Therapeutic Research* 2016;78:1-7.

10 Bang MD, Deyle GD. Comparison of supervised exercise with and without manual physical therapy for patients with shoulder impingement syndrome. *J Orthop Sports Phys Ther* 2000;30:126-137.

11 Başkurt Z, Başkurt F, Gelecek N, et al. The effectiveness of scapular stabilization exercise in the patients with subacromial impingement syndrome. *J Back Musculoskelet Rehabil* 2011;24:173-179.

12 Beyer R, Kongsgaard M, Hougs Kjær B, et al. Heavy slow resistance versus eccentric training as treatment for Achilles tendinopathy: a randomized controlled trial. *Am J Sports Med* 2015;43:1704-1711.

13 Blume C, Wang-Price S, Trudelle-Jackson E, et al. Comparison of eccentric and concentric exercise interventions in adults with subacromial impingement syndrome. *International journal of sports physical therapy* 2015;10:441.

14 Boudreau N, Gaudreault N, Roy J, et al. The Addition of Glenohumeral Adductor Coactivation to a Rotator Cuff Exercise Program for Rotator Cuff Tendinopathy: A Single-Blind Randomized Controlled Trial. *J Orthop Sports Phys Ther* 2019;49:126-135.

15 Breda SJ, Oei EHG, Zwerver J, et al. Effectiveness of progressive tendon-loading exercise therapy in patients with patellar tendinopathy: a randomised clinical trial. *Br J Sports Med* 2020;55:501-9.

16 Brox JI, Gjengedal E, Uppheim G, et al. Arthroscopic surgery versus supervised exercises in patients with rotator cuff disease (stage II impingement syndrome): a prospective, randomized, controlled study in 125 patients with a 2 1/2-year follow-up. *J Shoulder Elbow Surg* 1999;8:102-111.

17 Calis HT, Berberoglu N, Calis M. Are ultrasound, laser and exercise superior to each other in the treatment of subacromial impingement syndrome? A randomized clinical trial. *Eur J Phys Rehabil Med* 2011;47:375-380.

18 Chaconas EJ, Kolber MJ, Hanney WJ, et al. Shoulder external rotator eccentric training versus general shoulder exercise for subacromial pain syndrome: a randomized controlled trial. *International journal of sports physical therapy* 2017;12:1121-1133.

19 Cheng AS, Hung L. Randomized controlled trial of workplace-based rehabilitation for work-related rotator cuff disorder. *J Occup Rehabil* 2007;17:487-503.

20 Cho S, Shin Y. Effect of rehabilitation and prolotherapy on pain and functional performance in patients with chronic patellar tendinopathy. *Gazzetta Medica Italiana Archivio per le Scienze Mediche* 2017;176:330-337.

21 de Jonge, S. de Vos R, van Schie H,T.M. Verhaar, J, et al. One-year follow-up of a randomised controlled trial on added splinting to eccentric exercises in chronic midportion Achilles tendinopathy. *Br J Sports Med* 2008;44:673-677.

22 de Oliveira, FC L, Pairot de Fontenay B, Bouyer LJ, et al. Kinesiotaping for the Rehabilitation of Rotator Cuff-Related Shoulder Pain: A Randomized Clinical Trial. *Sports health* 2021;13:161-72.

23 De Vos RJ, Weir A, Visser R, et al. The additional value of a night splint to eccentric exercises in chronic midportion Achilles tendinopathy: a randomised controlled trial. *Br J Sports Med* 2007;41:e5

24 Dejaco B, Habets B, van Loon C, et al. Eccentric versus conventional exercise therapy in patients with rotator cuff tendinopathy: a randomized, single blinded, clinical trial. *Knee Surg Sports Traumatol Arthrosc* 2017;25:2051-2059.

25 Devereaux M, Velanoski KQ, Pennings A, et al. Short-Term Effectiveness of Precut Kinesiology Tape Versus an NSAID as Adjuvant Treatment to Exercise for Subacromial Impingement: A Randomized Controlled Trial. *Clin J Sport Med* 2016;26:24-32.

26 Dimitrios S, Pantelis M. Comparing Two Exercise Programmes for the Management of Lateral Elbow Tendinopathy (Tennis Elbow/Lateral Epicondylitis)—A Controlled Clinical Trial. The *Open Access Journal of Science and Technology* 2013;1:1-8.

27 Dimitrios S, Pantelis M, Kalliopi S. Comparing the effects of eccentric training with eccentric training and static stretching exercises in the treatment of patellar tendinopathy. A controlled clinical trial. *Clin Rehabil* 2012;26:423-430.

28 Dupuis F, Barrett E, Dubé M, et al. Cryotherapy or gradual reloading exercises in acute presentations of rotator cuff tendinopathy: a randomised controlled trial. *BMJ open sport & exercise medicine* 2018;4: e000477.

29 Engebretsen K, Grotle M, Bautz-Holter E, et al. Radial extracorporeal shockwave treatment compared with supervised exercises in patients with subacromial pain syndrome: single blind randomised study. *BMJ* 2009;339.

30 Engebretsen K, Grotle M, Bautz-Holter E, et al. Supervised exercises compared with radial extracorporeal shock-wave therapy for subacromial shoulder pain: 1-year results of a single-blind randomized controlled trial. *Phys Ther* 2011;91:37-47.

31 Gatz M, Betsch M, Dirrichs T, et al. Eccentric and Isometric Exercises in Achilles Tendinopathy Evaluated by the VISA-A Score and Shear Wave Elastography. *Sports health* 2020;12:373-81.

32 Giray E, Karali-Bingul D, Akyuz G. The Effectiveness of Kinesiotaping, Sham Taping or Exercises Only in Lateral Epicondylitis Treatment: A Randomized Controlled Study. *PM R* 2019;11:681-693.

33 Granviken F, Vasseljen O. Home exercises and supervised exercises are similarly effective for people with subacromial impingement: a randomised trial. *Journal of Physiotherapy* 2015;61:135-141.

34 Hallgren HC, Holmgren T, Oberg B, et al. A specific exercise strategy reduced the need for surgery in subacromial pain patients. *Br J Sports Med* 2014;48:1431-1436.

35 Hallgren HC, Adolfsson LE, Johansson K, et al. Specific exercises for subacromial pain: Good results maintained for 5 years. *Acta Orthopaedica* 2017;88:600-605.

36 Heron SR, Woby SR, Thompson DP. Comparison of three types of exercise in the treatment of rotator cuff tendinopathy/shoulder impingement syndrome: a randomised control trial assessing. *Physiotherapy* 2017;103:167-173.

37 Hotta GH, Gomes de Assis Couto A, Cools AM, et al. Effects of adding scapular stabilization exercises to a periscapular strengthening exercise program in patients with subacromial pain syndrome: A randomized controlled trial. *Musculoskelet Sci Pract* 2020;49:102171.

38 Johansson KM, Adolfsson LE, Foldevi MOM. Effects of acupuncture versus ultrasound in patients with impingement syndrome: randomized clinical trial. *Phys Ther* 2005;85:490-501.

39 Jonsson P, Alfredson H. Superior results with eccentric compared to concentric quadriceps training in patients with jumper's knee: a prospective randomised study. *Br J Sports Med* 2005;39:847-50

40 Jonsson P. Eccentric training in the treatment of tendinopathy (Doctoral dissertation, Department of Surgical and Perioperative Sciences, Sports Medicine). 2009.

41 Juul-Kristensen B, Larsen CM, Eshoj H, et al. Positive effects of neuromuscular shoulder exercises with or without EMG-biofeedback, on pain and function in participants with subacromial pain syndrome - A randomised controlled trial. *J Electromyogr Kinesiol* 2019;48:161-168.

42 Ketola S, Lehtinen J, Arnala I, et al. Does arthroscopic acromioplasty provide any additional value in the treatment of shoulder impingement syndrome?: a two-year randomised controlled trial. *TheJ Bone Joint Surg. British volume* 2009;91:1326-1334.

43 Ketola S, Lehtinen J, Rousi T, et al. No evidence of long-term benefits of arthroscopic acromioplasty in the treatment of shoulder impingement syndrome. *Bone & Joint Research* 2013;2:132-139.

44 Kim J, Shin D, Song C. Visual feedback to improve the effects of scapular stabilization exercises on pain intensity, range of motion, strength, and disability in patients with shoulder impingement syndrome. *Medical Science Technology* 2017;58:42-48.

45 Kim SY, Dvir Z, Oh JS. The application of the Neurac technique vs. manual therapy in patients during the acute phase of subacromial impingement syndrome: A randomized single-blinded controlled trial. *J Back Musculoskelet Rehabil*2019.

46 Knobloch K, Schreibmueller L, Longo UG, et al. Eccentric exercises for the management of tendinopathy of the main body of the Achilles tendon with or without an AirHeel Brace. A randomized controlled trial. B: effects of compliance. *Disabil Rehabil* 2008;30.

47 Knobloch K, Schreibmueller L, Kraemer R, et al. Eccentric training and an Achilles wrap reduce Achilles tendon capillary blood flow and capillary venous filling pressures and increase tendon oxygen saturation in insertional and midportion tendinopathy. *Am J Sports Med* 2007;35:673.

48 Knobloch K, Kraemer R, Jagodzinski M, et al. Eccentric training decreases paratendon capillary blood flow and preserves paratendon oxygen saturation in chronic achilles tendinopathy. J Orthop Sports *Phys Ther* 2007;37:269-276.

49 Kongsgaard M, Kovanen V, Aagaard P, et al. Corticosteroid injections, eccentric decline squat training and heavy slow resistance training in patellar tendinopathy. *Scand J Med Sci Sports* 2009;19:790-802.

50 Kromer TO, de Bie RA, Bastiaenen CHG. Effectiveness of physiotherapy and costs in patients with clinical signs of shoulder impingement syndrome: One-year follow-up of a randomized controlled trial. *J Rehabil Med* 2014;46:1029-1036.

51 Kromer TO, de Bie R,A., Bastiaenen CHG. Physiotherapy in patients with clinical signs of shoulder impingement syndrome: a randomized controlled trial. *J Rehabil Med* 2013;45:488-497.

52 Littlewood C, Bateman M, Brown K, et al. A self-managed single exercise programme versus usual physiotherapy treatment for rotator cuff tendinopathy: A randomised controlled trial (the SELF study). *Clin Rehabil* 2016;30:686-696.

53 Ludewig PM, Borstad JD. Effects of a home exercise programme on shoulder pain and functional status in construction workers. *Occup Environ Med* 2003;60:841-849.

54 Luginbuhl R, Brunner F, Schneeberger AG. No effect of forearm band and extensor strengthening exercises for the treatment of tennis elbow: a prospective randomised study. *Chir Organi Mov* 2008;91:35-40.

55 Maenhout AG, Mahieu NN, De Muynck M, et al. Does adding heavy load eccentric training to rehabilitation of patients with unilateral subacromial impingement result in better outcome? A randomized, clinical trial. *Knee Surg Sports Traumatol Arthrosc* 2013;21:1158-1167.

56 Mafi N, Lorentzon R, Alfredson H. Superior short-term results with eccentric calf muscle training compared to concentric training in a randomized prospective multicenter study on patients with chronic Achilles tendinosis. *Knee Surg Sports Traumatol Arthrosc* 2001;9:42-47.

57 Manias P, Stasinopoulos D. A controlled clinical pilot trial to study the effectiveness of ice as a supplement to the exercise programme for the management of lateral elbow tendinopathy [with consumer summary]. *Br J Sports Med* 2006;40:81-85.

58 Martinez-Silvestrini J, Newcomer KL, Gay RE, et al. Chronic lateral epicondylitis: comparative effectiveness of a home exercise program including stretching alone versus stretching supplemented with eccentric or concentric strengthening. *J Hand Ther* 2005;18:411-420.

59 Marzetti E, Rabini A, Piccinini G, et al. Neurocognitive therapeutic exercise improves pain and function in patients with shoulder impingement syndrome: a single-blind randomized controlled clinical trial. *Eur J Phys Rehabil Med* 2014;50:255-264.

60 McCormack JR, Underwood FB, Slaven EJ, et al. Eccentric Exercise Versus Eccentric Exercise and Soft Tissue Treatment (Astym) in the Management of Insertional Achilles Tendinopathy. *Sports health* 2016;8:230-7.

61 Melegati G, Tornese D, Bandi M. Effectiveness of extracorporeal shock wave therapy associated with kinesitherapy in the treatment of subacromial impingement: A randomised, controlled study. *Journal of Sports Traumatology and Related Research* 2000;22:58-64.

62 Mulligan EP, Huang M, Dickson T, et al. The Effect of Axioscapular and Rotator Cuff Exercise Training Sequence in Patients with Subacromial Impingement Syndrome: a Randomized Crossover Trial. *International journal of sports physical therapy* 2016;11:94-107.

63 Nishizuka T, Iwatsuki K, Kurimoto S, et al. Efficacy of a forearm band in addition to exercises compared with exercises alone for lateral epicondylitis: A multicenter, randomized, controlled trial. *J Orthop Sci* 2017;22:289-294.

64 Nørregaard J, Larsen CC, Bieler T, et al. Eccentric exercise in treatment of Achilles tendinopathy. *Scand J Med Sci Sports* 2007;17:133-138.

65 Nowotny J, El-Zayat B, Goronzy J, et al. Prospective randomized controlled trial in the treatment of lateral epicondylitis with a new dynamic wrist orthosis. *Eur J Med Res* 2018;23:1-7.

66 Østerås H, Torstensen TA, Østerås B. High-dosage medical exercise therapy in patients with long-term subacromial shoulder pain: a randomized controlled trial. *Physiother Res Int* 2010;15:232-242.

67 Paavola M, Malmivaara A, Taimela S, et al. Subacromial decompression versus diagnostic arthroscopy for shoulder impingement: randomised, placebo surgery controlled clinical trial. *BMJ* 2018;19:362.

68 Park JY, Park HK, Choi JH, et al. Prospective evaluation of the effectiveness of a home-based program of isometric strengthening exercises: 12-month follow-up. *Clin Orthop Surg* 2010;2:173-178.

69 Pearson J, Rowlands D, Highet R. Autologous blood injection to treat achilles tendinopathy? A randomized controlled trial. *J Sport Rehab* 2012;21:218-24.

70 Pearson SJ, Stadler S, Menz H, et al. Immediate and Short-Term Effects of Short-and Long-Duration Isometric Contractions in Patellar Tendinopathy. *Clin J Sport Med* 2018.

71 Pekyavas NO, Baltaci G. Short-term effects of high-intensity laser therapy, manual therapy, and Kinesio taping in patients with subacromial impingement syndrome. *Lasers in medical science* 2016;31:1133-1141.

72 Petersen W, Welp R, Rosenbaum D. Chronic Achilles tendinopathy: a prospective randomized study comparing the therapeutic effect of eccentric training, the AirHeel brace, and a combination of both. *Am J Sports Med* 2007;35:1659-1667.

73 Peterson M, Butler S, Eriksson M, et al. A randomized controlled trial of exercise versus wait-list in chronic tennis elbow (lateral epicondylosis). *Ups J Med Sci* 2011;116:269-279.

74 Peterson M, Butler S, Eriksson M, et al. A randomized controlled trial of eccentric versus concentric graded exercise in chronic tennis elbow (lateral elbow tendinopathy) [with consumer summary]. *Clin Rehabil* 2014;28:862-872 2014.

75 Polimeni V, Panuccio A, Furfari P, et al. Preliminary study on the efficacy of various rehabilitation therapies for shoulder pain. *Europa Medicophysica* 2003;39:59-63.

76 Praet S, Alzyadat T, Purdam C, et al. Oral supplementation of specific collagen peptides accelerates improvement in Achilles tendon pain and function in combination with a tailored exercise program. *J Bodywork Movement Ther* 2018;22:862-3.

77 Rabusin CL, Menz HB, McClelland JA, et al. Efficacy of heel lifts versus calf muscle eccentric exercise for mid-portion Achilles tendinopathy (HEALTHY): a randomised trial. *Br J Sports Med* 2020:55:486-92.

78 Reyhan AC, Sindel D, Dereli EE. The effects of Mulligan's mobilization with movement technique in patients with lateral epicondylitis. *J Back Musculoskelet Rehabil*2020;33:99-107.

79 Rio E, Purdam C, Girdwood M, et al. Isometric Exercise to Reduce Pain in Patellar Tendinopathy In-Season; Is It Effective "on the Road?". *Clin J Sport Med* 2017;29:188-92

80 Romero-Morales C, Martin-Llantino P, Calvo-Lobo C, et al. Vibration increases multifidus cross-sectional area versus cryotherapy added to chronic non-insertional Achilles tendinopathy eccentric exercise [with consumer summary]. *Phys Ther Sport* 2020;42:61-67.

81 Rompe JD, Nafe B, Furia JP, Maffuli N. Eccentric loading, shock-wave treatment, or a wait-and-see policy for tendinopathy of the main body of tendo Achillis: A randomized controlled trial. *Am J Sports Med* 2007;35:374-383.

82 Rompe JD, Furia J, Maffulli N. Eccentric loading compared with shock wave treatment for chronic insertional achilles tendinopathy: A randomized, controlled trial.*J Bone Joint Surg* 2008;90:52-61.

83 Rompe JD, Furia J, Maffulli N. Eccentric loading versus eccentric loading plus shock-wave treatment for midportion achilles tendinopathy: A randomized controlled trial. *Am J Sports Med* 2009;37:463-470.

84 Roos EM, Engstrom M, Lagerquist A, et al. Clinical improvement after 6 weeks of eccentric exercise in patients with mid-portion Achilles tendinopathy: A randomized trial with 1-year follow-up. *Scand J Med Sci Sports* 2004;14:286-295.

85 Şenbursa G, Baltaci G, Atay ÖA. The effectiveness of manual therapy in supraspinatus tendinopathy. Acta *Orthop Traumatol Turc* 2011;45:162-167.

86 Seven MM, Ersen O, Akpancar S, et al. Effectiveness of prolotherapy in the treatment of chronic rotator cuff lesions. *Orthop Traumatol Surg Res* 2017;103:427-433.

87 Sevier TL, Stegink-Jansen C. Astymtreatment vs. eccentric exercise for lateral elbow tendinopathy: a randomized controlled clinical trial. Peer J 2015;3:e967.

88 Silbernagel KG, Thomeé R, Eriksson BI, et al. Continued sports activity, using a pain-monitoring model, during rehabilitation in patients with Achilles tendinopathy: a randomized controlled study. *Am J Sports Med* 2007;35:897-906.

89 Silbernagel KG, Thomeé R, Thomeé P, et al. Eccentric overload training for patients with chronic Achilles tendon pain--a randomised controlled study with reliability testing of the evaluation methods. *Scand J Med Sci Sports* 2001;11:197-206.

90 Şimşek HH, Balki S, Keklik SS, et al. Does Kinesio taping in addition to exercise therapy improve the outcomes in subacromial impingement syndrome? A randomized, double-blind, controlled clinical trial. Acta *Orthop Traumatol Turc* 2013;47:104-110.

91 Stasinopoulos D, Stasinopoulos I. Comparison of effects of Cyriax physiotherapy, a supervised exercise programme and polarized polychromatic non-coherent light (Bioptron light) for the treatment of lateral epicondylitis. *Clin Rehabil* 2006;20:12-23.

92 Stasinopoulos D, Stasinopoulos I, Pantelis M, et al. Comparison of effects of a home exercise programme and a supervised exercise programme for the management of lateral elbow tendinopathy. *Br J Sports Med* 2010;44:579-583.

93 Stasinopoulos D, Stasinopoulos I. Comparison of effects of eccentric training, eccentric-concentric training, and eccentric-concentric training combined with isometric contraction in the treatment of lateral elbow tendinopathy. *J Hand Ther* 2017;30:13-19.

94 Stefansson SH, Brandsson S, Langberg H, et al. Using Pressure Massage for Achilles Tendinopathy: A Single-Blind, Randomized Controlled Trial Comparing a Novel Treatment Versus an Eccentric Exercise Protocol. *Orthopaedic journal of sports medicine* 2019;7:2325967119834284.

95 Steunebrink M, Zwerver J, Brandsema R, et al. Topical glyceryl trinitrate treatment of chronic patellar tendinopathy: a randomised, double-blind, placebo-controlled clinical trial. *Br J Sports Med* 2013;47:34-39.

96 Stevens M, Tan C. Effectiveness of the Alfredson protocol compared with a lower repetition-volume protocol for midportion Achilles tendinopathy: a randomized controlled trial. *J Orthop Sports Phys Ther* 2014;44:59-67.

97 Svernlov B, Adolfsson L. Non-operative treatment regime including eccentric training for lateral humeral epicondylalgia. *Scand J Med Sci Sports* 2001;11:328-334.

98 Tahran Ö, Yeşilyaprak SS. Effects of Modified Posterior Shoulder Stretching Exercises on Shoulder Mobility, Pain, and Dysfunction in Patients With Subacromial Impingement Syndrome. *Sports health* 2020;12:139-148.

99 Tonks JH, Pai SK, Murali SR. Steroid injection therapy is the best conservative treatment for lateral epicondylitis: A prospective randomised controlled trial. *Int J Clin Pract* 2007;61:240-246.

100 Turgut E, Duzgun I, Baltaci G. Effects of Scapular Stabilization Exercise Training on Scapular Kinematics, Disability, and Pain in Subacromial Impingement: A Randomized Controlled Trial. *Arch Phys Med Rehabil* 2017;98:1915.

101 Vallés-Carrascosa E, Gallego-Izquierdo T, Jiménez-Rejano JJ, et al. Pain, motion and function comparison of two exercise protocols for the rotator cuff and scapular stabilizers in patients with subacromial syndrome. *J Hand Ther* 2018;31:227-37.

102 Van Ark M, Cook JL, Docking SI, et al. Do isometric and isotonic exercise programs reduce pain in athletes with patellar tendinopathy in-season? A randomised clinical trial. J Sci Med Sport 2016;19:702-706.

103 Vinuesa-Montoya S, Aguilar-Ferrándiz ME, Matarán-Peñarrocha GA, et al. A Preliminary Randomized Clinical Trial on the Effect of Cervicothoracic Manipulation Plus Supervised Exercises Vs a Home Exercise Program for the Treatment of Shoulder Impingement. *Journal of chiropractic medicine.* 2017;16:85-93.

104 Visnes H, Hoksrud A, Cook J, et al. No effect of eccentric training on jumper's knee in volleyball players during the competitive season: a randomized clinical trial. *Scand J Med Sci Sports* 2005;15:215.

105 Vuvan, V, Vicenzino B, Mellor R, et al. Unsupervised Isometric Exercise versus Wait-and-See for Lateral Elbow Tendinopathy. *Med Sci Sports Exerc* 2020;52:287-295.

106 Walther M, Werner A, Stahlschmidt T, et al. The subacromial impingement syndrome of the shoulder treated by conventional Physiotherapy, self-training, and a shoulder brace: Results of a prospective, randomized study. *J Shoulder Elbow Surg*2004;13:417-423.

107 Wegener RL, Brown T, O'Brien L. A randomized controlled trial of comparative effectiveness of elastic therapeutic tape, sham tape or eccentric exercises alone for lateral elbow tendinosis. *Hand Therapy* 2016;21:131-139.

108 Wen DY, Schultz BJ, Schaal B, et al. Eccentric strengthening for chronic lateral epicondylosis: a prospective randomized study. *Sports health* 2011;3:500-503.

109 Werner A, Walther M, Ilg A, et al. Self-training versus conventional Physiotherapy in subacromial impingement syndrome. *Z Orthop Ihre* 2002;140:375-380.

110 Wiedmann M, Mauch F, Huth J, et al. Treatment of mid-portion Achilles tendinopathy with eccentric training and its effect on neovascularization. *Sports Orthopaedics and Traumatolog*y 2017;33:278-285.

111 Yelland MJ, Sweeting KR, Lyftogt JA, et al. Prolotherapy injections and eccentric loading exercises for painful Achilles tendinosis: a randomised trial. *Br J Sports Med* 2011;45: 421-8.

112 Yerlikaya M, Talay Çaliş H, Tomruk Sütbeyaz S, et al. Comparison of Effects of Leukocyte-Rich and Leukocyte-Poor Platelet-Rich Plasma on Pain and Functionality in Patients With Lateral Epicondylitis. *Archives of rheumatology* 2017;33:73-79.

113 Young MA, Cook JL, Purdam CR, et al. Eccentric decline squat protocol offers superior results at 12 months compared with traditional eccentric protocol for patellar tendinopathy in volleyball players [with consumer summary]. *Br J Sports Med* 2005;39:102-105.

114 Yu J, Park D, Lee G. Effect of eccentric strengthening on pain, muscle strength, endurance, and functional fitness factors in male patients with achilles tendinopathy. *Am J Phys Med Rehabil* 2013;92:68-76.

Supplementary file 10: List of excluded studies with reasons

**Citation**	**Exclusion reason**
Walther M, Werner A, Stahlschmidt T, *et al.* The subacromial impingement syndrome of the shoulder treated by conventional physiotherapy, self-training, and a shoulder brace: results of a prospective, randomized study. *J Shoulder Elbow Surg* 2004;1:417-23.	Duplicate
van Ark M. Patellar tendinopathy: Physical therapy and injection treatments (Doctoral dissertation, University of Groningen).2015:1-136.	Duplicate
Jensen B, Bliddal H, Danneskiold-Samsøe B. Comparison of two different treatments of lateral humeral epicondylitis--" tennis elbow". A randomized controlled trial. *Ugeskr Laeg* 2001;1:1427-31.	Duplicate
Frohm A, Saartok T, Halvorsen K, *et al.* Eccentric treatment for patellar tendinopathy: a prospective randomised short-term pilot study of two rehabilitation protocols. *Br J Sports Med* 2007;41:e7.	Duplicate
Cannell LJ, Taunton JE, Clement DB, *et al.* A randomised clinical trial of the efficacy of drop squats or leg extension/leg curl exercises to treat clinically diagnosed jumper's knee in athletes: pilot study. *Br J Sports Med* 2001;35:60-64.	Duplicate
Stasinopoulos D, Manias P. Comparing two eccentric exercise programmes for the management of Achilles tendinopathy. A pilot trial. *J Bodyw Mov Ther* 2013;17:309-115.	Duplicate
Manias P, Stasinopoulos D. A controlled clinical pilot trial to study the effectiveness of ice as a supplement to the exercise programme for the management of lateral elbow tendinopathy. *Br J Sports Med* 2006;40:81-85.	Duplicate
Stergioulas A, Stergioula M, Aarskog R, *et al.* Effects of low-level laser therapy and eccentric exercises in the treatment of recreational athletes with chronic achilles tendinopathy. *Am J Sports Med* 2008;36:881–7.	Duplicate
Jonsson P, Alfredson H. Superior results with eccentric compared to concentric quadriceps training in patients with jumper's knee: a prospective randomised study. *Br J Sports Med* 2005;39:847–50	Duplicate
Heron SR, Woby SR, Thompson DP. Comparison of three types of exercise in the treatment of rotator cuff tendinopathy/shoulder impingement syndrome: A randomized controlled trial. *Physiotherapy* 2017;103:167–73.	Duplicate
Ganderton C, Semciw A, Cook J, *et al.* Gluteal loading versus sham exercises to improve pain and dysfunction in postmenopausal women with greater trochanteric pain syndrome: a randomized controlled trial. *J Women's Heal* 2018;27:815–29.	Duplicate
de Vos RJ, Weir A, van Schie HT, *et al.* Platelet-rich plasma injection for chronic Achilles tendinopathy. *J - Am Med Assoc* 2010;303:144–9.	Duplicate
Balius R, Álvarez G, Baró F, *et al.* A 3-Arm Randomized Trial for Achilles Tendinopathy: Eccentric Training, Eccentric Training Plus a Dietary Supplement Containing Mucopolysaccharides, or Passive Stretching Plus a Dietary Supplement Containing Mucopolysaccharides. *Curr Ther Res* 2016;78:1–7.	Duplicate
Tumilty S, Mani R, Baxter GD. Photobiomodulation and eccentric exercise for Achilles tendinopathy: a randomized controlled trial. *Lasers Med Sci* 2016;31:127–135.	Duplicate

van Ark M, Rio E, Cook J, et al. Clinical improvements are not explained by changes in tendon structure on UTC following an exercise program for patellar tendinopathy. *Am J Phys Med* 2018;97:708-714.	Duplicate
Blume CL. Comparison of an eccentric exercise intervention to a concentric exercise intervention in adults with subacromial impingement syndrome (Doctoral dissertation, Texas Woman's University). 2014:1-218.	Duplicate
Coombes BK, Bisset L, Brooks P, Khan A, Vicenzino B. Effect of corticosteroid injection, physiotherapy, or both on clinical outcomes in patients with unilateral lateral epicondylalgia: a randomized controlled trial. *JAMA*. 2013;309:461-469.	Duplicate
Berg OK, Paulsberg F, Brabant C, *et al.* High-Intensity Shoulder Abduction Exercise in Subacromial Pain Syndrome. *Med Sci Sports Exerc* 2021 ;53:1-9.	Insufficient exercise data
Jensen B, Bliddal H, Danneskiold-Samsøe B. Comparison of two different treatments of lateral humeral epicondylitis--" tennis elbow". A randomized controlled trial. *Ugeskr Laeg* 2001;163:1427-1431.	Insufficient exercise data
Chapman-Jones D, Hill D. Novel microcurrent treatment is more effective than conventional therapy for chronic Achilles tendinopathy: randomised comparative trial. *Physiotherapy* 2002;1:471-80.	Insufficient exercise data
Kumar N, Nehru A, Rajalakshmi D. Effect of taping as a component of conservative treatment for subacromial impingement syndrome. *Health* 2012;26:237-241.	Insufficient exercise data
Schmitt J, Haake M, Tosch A, *et al.* Low-energy extracorporeal shock-wave treatment (ESWT) for tendinitis of the supraspinatus: a prospective, randomised study. *J Bone Surg Joint Am* 2001;83:873-876.	Insufficient exercise data
Kolk A, Auw Yang KG, Tamminga R, *et al.* Radial extracorporeal shock-wave therapy in patients with chronic rotator cuff tendinitis: a prospective randomised double-blind placebo-controlled multicentre trial. *Bone Joint J* 2013;95:1521-6.	Insufficient exercise data
Tetschke E, Rudolf M, Lohmann CH, *et al.* Autologous proliferative therapies in recalcitrant lateral epicondylitis. *Am J Phys Med Rehabil* 2015;1:696-706.	Insufficient exercise data
Coff L, Massy-Westropp N, Caragianis S. Randomized controlled trial of a new electrical modality (InterX) and soft tissue massage, stretching, ultrasound and exercise for treating lateral epicondylitis. *Hand Ther* 2009;14:46-52.	Insufficient exercise data
Furia JP. High-energy extracorporeal shock wave therapy as a treatment for insertional Achilles tendinopathy. *Am J Sports Med* 2006;34:733-740.	Insufficient exercise data
Cloke DJ, Watson H, Purdy S, *et al.* A pilot randomized, controlled trial of treatment for painful arc of the shoulder. *J Shoulder Elbow Surg* 2008;17:S17-21.	Insufficient exercise data
Krogh TP, Ellingsen T, Christensen R, *et al.* Ultrasound-guided injection therapy of Achilles tendinopathy with platelet-rich plasma or saline: a randomized, blinded, placebo-controlled trial. *Am J Sports Med* 2016;44:1990-1997.	Insufficient exercise data
Rasmussen S, Christensen M, Mathiesen I, *et al.* Shockwave therapy for chronic Achilles tendinopathy: a double-blind, randomized clinical trial of efficacy. *Acta Orthop* 2008;79:249-256.	Insufficient exercise data
Branson R, Naidu K, du Toit C, *et al.* Comparison of corticosteroid, autologous blood or sclerosant injections for chronic tennis elbow. *J Sci Med Sport* 2017;20:528-533.	Insufficient exercise data
Yuksel E, Yesilyaprak SS. The Effectiveness of Scapular Stabilization Exercises in Patients with Subacromial Impingement Syndrome and Scapular Dyskinesis. *Ann Rheum Dis* 2015;74:1316.	Insufficient exercise data

Dragoo JL, Braun HJ, Wasterlain AS. Platelet-Rich Plasma as a Treatment for Patellar Tendinopathy: A Double-Blind Randomized Controlled Trial. *Am J Sports Med* 2014;42:610-618.	Insufficient exercise data
Selvanetti A, Barrucci A, Antonaci A, *et al.* L'esercizio eccentrico nella rieducazione funzionale dell' epicondilite: studio randomizzato controllato (Role of the eccentric exercise in the functional reeducation of lateral epicondylitis: a randomised controlled clinical trial) [Italian]. *Med Dello Sport* 2003;56:103–13.	Insufficient exercise data
van der Vlist AC, Veldhoven PLJ, Oosterom RF, *et al.* Isometric exercises do not provide immediate pain relief in Achilles tendinopathy: A quasi-randomized clinical trial. *Scand J Med Sci Sports* 2020;30:1712–21.	Insufficient exercise data
Kim S, Kwon O, Weon J, *et al*. The effect of the neurac training on shoulder isokinetic performance in patients with acute-phase subacromial impingement syndrome [Absract]. *Man Ther* 2016;25:e59 https://www.infona.pl/resource/bwmeta1.element.elsevier-7217cec5-508b-3ebf-bb33-8aef41a575e1 (accessed 01 Jul 2021)	Insufficient exercise data
Eraslan L, Baltaci G, Yuce D, *et al.* Effects of Physiotherapy Approaches on Pain and Strength in Lateral Epicondylitis: A Randomized Clinical Trial [abstract]. *Med Sci Sport Exerc* 2015;47:614.	Insufficient exercise data
Prat PI, Cibrowski D, Zuliani A, *et al.* Efficacy of fascial manipulation and eccentric exercise for lateral elbow pain. *J Bodyw Mov Ther* 2018;22:855.	Insufficient exercise data
Apostolos S. The influence of low level laser and pyrometric exercises in the treatment of patients with tennis elbow. a pilot study. 2004. http://cev.org.br/biblioteca/the-influence-of-low-level-laser-and-plyometric-exercises-in-the-treatment-of-patients-with-tennis-elbow-pilot-study/ (accessed 21 Jun 2021)	Insufficient exercise data
Subaşi V, Toktaş H, Demirdal ÜS, *et al.* Water-Based versus Land-Based Exercise Program for the Management of Shoulder Impingement Syndrome. / Omuz Subakromiyal Sıkışma Sendromunun Tedavisinde Su İçi Egzersizler ile Kara Egzersizlerinin Karşılaştırılması. *Turkish J Phys Med Rehabil* 2012;58:79–84.	Insufficient exercise data
Chung B, Wiley JP, Rose MS. Long-term effectiveness of extracorporeal shockwave therapy in the treatment of previously untreated lateral epicondylitis. *Clin J Sport Med* 2005;15:305–12.	Insufficient exercise data
Croisier JL, Forthomme B, Foidart-Dessalle M, *et al.* Isokinetic eccentric exercises in treating chronic tendinitis [Abstract]. *Isokinet Exerc Sci* 2002;10:25–6.	Insufficient exercise data
Baumer TG, Peltz CD, Drake A, *et al.* Effects of Rotator Cuff Pathology and Physical Therapy on In Vivo Shoulder Motion and Clinical Outcomes in Patients With a Symptomatic Full-Thickness Rotator Cuff Tear. *Orthop J Sports Med* 2016;4: 2325967116666506.	Insufficient exercise data
Entrellardat Tortillol E. Effectiveness of percutaneus needle electrolysis and eccentric exercise in chronic patellar tendinopathy. *Rev Fisioter Invasiva / J Invasive Tech Phys Ther* 2019;02:75.	Insufficient exercise data
Crawshaw DP, Helliwell PS, Hensor EMA, *et al.* Exercise therapy after corticosteroid injection for moderate to severe shoulder pain: large pragmatic randomised trial. *BMJ* 2010;340:e3037–e3037.	Insufficient exercise data
Başkurt F, Özcan A, Algun C. Comparison of effects of phonophoresis and iontophoresis of naproxen in the treatment of lateral epicondylitis. *Clin Rehabil*	Insufficient exercise data

2003;17:96–100.	
Thompson G, Pearson JF. No attributable effects of PRP on greater trochanteric pain syndrome. *N Z Med J* 2019;132:22–32.	Insufficient exercise data
Yildirim MA, Ones K, Celik EC. Comparison of ultrasound therapy of various durations in the treatment of subacromial impingement syndrome. *J Phys Ther Sci* 2013;25:1151–4.	Insufficient exercise data
Barra López ME, López de Celis C, Fernández Jentsch G, *et al.* Effectiveness of Diacutaneous Fibrolysis for the treatment of subacromial impingement syndrome: a randomised controlled trial. *Man Ther* 2013;18:418–24.	Insufficient exercise data
Bostrøm K, Mæhlum S, Småstuen MC, *et al.* Clinical comparative effectiveness of acupuncture versus manual therapy treatment of lateral epicondylitis: feasibility randomized clinical trial. *Pilot feasibility Stud* 2019;5:110.	Insufficient exercise data
Thanasas C, Papadimitriou G, Charalambidis C, *et al.* Platelet-rich plasma versus autologous whole blood for the treatment of chronic lateral elbow epicondylitis: a randomized controlled clinical trial. *Am J Sports Med* 2011;39:2130–4.	Insufficient exercise data
Riley SP, Cote MP, Leger RR, *et al.* Short-term effects of thoracic spinal manipulations and message conveyed by clinicians to patients with musculoskeletal shoulder symptoms: a randomized clinical trial. *J Man Manip Ther* 2015;23:3–11.	Insufficient exercise data
Bisset LM, Coppieters MW, Vicenzino B. Sensorimotor deficits remain despite resolution of symptoms using conservative treatment in patients with tennis elbow: A randomized controlled trial. *Arch Phys Med Rehabil* 2009;90:1–8.	Insufficient exercise data
Littlewood C, Malliaras P, Mawson S, *et al.* Self-managed loaded exercise versus usual physiotherapy treatment for rotator cuff tendinopathy: a pilot randomised controlled trial. *Physiotherapy* 2014;100:54–60.	Insufficient exercise data
Brown R, Orchard J, Kinchington M, *et al.* Aprotinin in the management of Achilles tendinopathy: a randomised controlled trial. *Br J Sports Med* 2006;40:275-279.	Insufficient exercise data
Aytar A, Baltaci G, Uhl TL, *et al.* The effects of scapular mobilization in patients with subacromial impingement syndrome: a randomized, double-blind, placebo-controlled clinical trial. *J Sport Rehabil* 2015;24:116–29.	Insufficient exercise data
O'Neill S, Watson P, Barry S. Eccentric rehabilitation for runners with Achilles tendinopathy improves endurance capacity of the plantarflexors [Abstract]. *Physiotherapy* 2015;101:e1143–4.	Insufficient exercise data
Jerosch J, Wustner P. The effect of a sensorimotor exercise program in patients with subacromial pain syndrome. *Unfallchirurg* 2002;105:36–43.	Insufficient exercise data
Rio E, Kidgell D, Purdam C, *et al.* Isometric exercise induces analgesia and reduces inhibition in patellar tendinopathy. *Br J Sports Med* 2015;49:1277–1283.	Insufficient exercise data
Özgen M, Fırat S, Sarsan A, *et al.* Short- and long-term results of clinical effectiveness of sodium hyaluronate injection in supraspinatus tendinitis. *Rheumatol Int* 2012;32:137–44.	Insufficient exercise data
Young M, Cook J, Purdam C, *et al.* Conservative treatment of patellar tendinopathy: A randomised trial comparing two treatment regimes [Abstract]. *J Sci Med Sport* 2002;5:120.	Insufficient exercise data

Turgut E, Duzgun I. AB1428-HPR Two-year follow-up of the therapeutic exercise program for patients with rotator cuff tendinopathy: a single group study to investigate the effects on pain and disability. In: *Saturday, 16 JUNE 2018*. BMJ Publishing Group Ltd and European League Against Rheumatism 2018. 1847.3-1848.	Insufficient exercise data
Wang CJ, Ko JY, Chan YS, *et al.* Extracorporeal shockwave for chronic patellar tendinopathy. *Am J Sports Med* 2007;35.	Insufficient exercise data
López-de-Celis C, Barra-López ME, González-Rueda V, *et al.* Effectiveness of diacutaneous fibrolysis for the treatment of chronic lateral epicondylalgia: a randomized clinical trial. *Clin Rehabil* 2018;32:644–53.	Insufficient exercise data
Ram R, Meeuwisse W, Patel C, *et al.* The Limited Effectiveness of a Home-Based Eccentric Training for Treatment of Achilles Tendinopathy. *Clin Investig Med* 2013;36:197.	Insufficient exercise data
Razavi M, Jansen GB. Effects of acupuncture and placebo TENS in addition to exercise in treatment of rotator cuff tendinitis. *Clin Rehabil* 2004;18:872–8.	Insufficient exercise data
Canbulat N, Seyahi A, Eren SM, *et al.* 24. The effect of core stabilization exercises in the rehabilitation of patients with subacromial impingement syndrome [Abstract]. *Türkiye Fiz Tıp ve Rehabil Derg* 2013;59:431.	Insufficient exercise data
Paoloni JA, Appleyard RC, Nelson J, *et al.* Topical Glyceryl Trinitrate Treatment of Chronic Noninsertional Achilles Tendinopathy. *J Bone Jt Surg* 2004;86:916–22.	Insufficient exercise data
Leduc BE, Caya J, Tremblay S, *et al.* Treatment of calcifying tendinitis of the shoulder by acetic acid iontophoresis: a double-blind randomized controlled trial. *Arch Phys Med Rehabil* 2003;84:1523–7.	Insufficient exercise data
Saggini R, Di Stefano A, Galati V, *et al.* Long-term effectiveness of combined mechanotransduction treatment in jumper's knee. *Eur J Inflamm* 2012;10:515–24.	Insufficient exercise data
Dragoo JL, Wasterlain AS, Braun HJ, *et al.* Platelet-Rich Plasma as a Treatment for Patellar Tendinopathy. *Am J Sports Med* 2014;42:610–8.	Insufficient exercise data
Solomons L, Lee JJY, Bruce M, *et al.* Intramuscular stimulation vs sham needling for the treatment of chronic midportion Achilles tendinopathy: A randomized controlled clinical trial. PloS One 2020;15: e0238579	Insufficient exercise data
Kvalvaag E, Roe C, Engebretsen KB, *et al.* One year results of a randomized controlled trial on radial Extracorporeal Shock Wave Treatment, with predictors of pain, disability and return to work in patients with subacromial pain syndrome. *Eur J Phys Rehabil Med* 2018;54:341–50.	Insufficient exercise data
González PP, Brahim MB. Treatment of Shoulder Impingement Syndrome in Adolescent Tennis Players. / Tractament de la síndrome subacromial en tennistes adolescents. *Apunt Educ Física i Esports* 2018;132:32–47.	Insufficient exercise data
Cook C, Learman K, Houghton S, *et al.* The addition of cervical unilateral posterior–anterior mobilisation in the treatment of patients with shoulder impingement syndrome: A randomised clinical trial. *Man Ther* 2014;19:18–24 .	Insufficient exercise data
Oken O, Kahraman Y, Ayhan F, *et al.* The Short-term Efficacy of Laser, Brace, and Ultrasound Treatment in Lateral Epicondylitis: A Prospective, Randomized, Controlled Trial. *J Hand Ther* 2008;21:63–8.	Insufficient exercise data
Di Lorenzo L, Pappagallo M, Gimigliano R, *et al.* Pain relief in early rehabilitation of rotator cuff tendinitis: any role for indirect suprascapular nerve	Insufficient exercise data

block? *Eura Medicophys* 2006;42:195–204.	
Kvalvaag E, Brox JI, Engebretsen KB, *et al.* Effectiveness of radial extracorporeal shock wave therapy (rESWT) when combined with supervised exercises in patients with subacromial shoulder pain. *Am J Sports Med* 2017;45:2547–54.	Insufficient exercise data
Kesikburun S, Tan AK, Yilmaz B, *et al.* Platelet-rich plasma injections in the treatment of chronic rotator cuff tendinopathy: a randomized controlled trial with 1-year follow-up. *Am J Sports Med* 2013;41:2609–16.	Insufficient exercise data
Munteanu SE, Scott LA, Bonanno DR, *et al.* Effectiveness of customised foot orthoses for Achilles tendinopathy: a randomised controlled trial. *Br J Sports Med* 2015;49:989–94.	Insufficient exercise data
Kim SJ, Yeo SM, Noh SJ, *et al.* Effect of platelet-rich plasma on the degenerative rotator cuff tendinopathy according to the compositions. *J Orthop Surg Res* 2019;14:408.	Insufficient exercise data
Al Dajah SB. Soft tissue mobilization and PNF improve range of motion and minimize pain level in shoulder impingement. *J Phys Ther Sci* 2014;26:1803–5.	Insufficient exercise data
Johansson K, Bergström A, Schröder K, *et al.* Subacromial corticosteroid injection or acupuncture with home exercises when treating patients with subacromial impingement in primary care--a randomized clinical trial. *Fam Pract* 2011;28:355–65.	Insufficient exercise data
Weir A, Jansen J, Van de Port IGL, *et al.* Manual or exercise therapy for long-standing adductor-related groin pain: a randomised controlled clinical trial. *Man Ther* 2011;16:148–54.	Insufficient exercise data
Hernández Herrero D, Berjillos Donamayor A, de la Corte Rodríguez H, *et al.* Elbow tendinosis treated by several electrotherapy techniques: a prospective randomized study. 2006;4:131–138	Insufficient exercise data
Bal A, Eksioglu E, Gurcay E, *et al.* Low-level laser therapy in subacromial impingement syndrome. *Photomed Laser Surg* 2009;27:31–6.	Insufficient exercise data
Güler H, Turhanoğlu AD, Inanoğllu K, *et al.* Comparison of ketoprofen phonophoresis with ketoprofen and lidocaine- prilocaine phonophoresis in patients with subacromial impingement syndrome. *Turkish J Rheumatol* 2009;24:88–93.	Insufficient exercise data
Elsodany AM, Alayat MSM, Ali MME, *et al.* Long-Term Effect of Pulsed Nd:YAG Laser in the Treatment of Patients with Rotator Cuff Tendinopathy: A Randomized Controlled Trial. *Photomed Laser Surg* 2018;36:506–13.	Insufficient exercise data
Citaker S, Taskiran H, Akdur H, *et al.* Comparison of the mobilization and proprioceptive neuromuscular facilitation methods in the treatment of shoulder impingement syndrome. *Pain Clin* 2005;17:197–202.	Insufficient exercise data
Pienimaki T, Karinen P, Kemila T, *et al.* Long-term follow-up of conservatively treated chronic tennis elbow patients. A prospective and retrospective analysis. *Scand J Rehabil Med* 1998;30:159–66.	Insufficient exercise data
Akgün K, Birtane M, Akarirmak U. Is local subacromial corticosteroid injection beneficial in subacromial impingement syndrome? *Clin Rheumatol* 2004;23:496–500.	Insufficient exercise data
Taskaynatan MA, Ozgul A, Ozdemir A, *et al.* Effects of Steroid Iontophoresis and Electrotherapy on Bicipital Tendonitis. *J Musculoskelet Pain* 2007;15:47–54.	Insufficient exercise data

Mellor R, Bennell K, Grimaldi A, *et al.* Education plus exercise versus corticosteroid injection use versus a wait and see approach on global outcome and pain from gluteal tendinopathy: prospective, single blinded, randomised clinical trial. *Br J Sports Med* 2018;52:1464–72.	Insufficient exercise data
Ohberg L, Lorentzon R, Alfredson H. Eccentric training in patients with chronic Achilles tendinosis: normalised tendon structure and decreased thickness at follow up. *Br J Sports Med* 2004;38:8–11.	Insufficient exercise data
van der Worp H, Zwerver J, Hamstra M, *et al.* No difference in effectiveness between focused and radial shockwave therapy for treating patellar tendinopathy: a randomized controlled trial. *Knee Surgery, Sport Traumatol Arthrosc* 2014;22:2026–32.	Insufficient exercise data
Szczurko O, Cooley K, Mills EJ, *et al.* Naturopathic treatment of rotator cuff tendinitis among Canadian postal workers: A randomized controlled trial. *Arthritis Rheum* 2009;61:1037–45.	Insufficient exercise data
Newcomer KL, Laskowski ER, Idank DM, *et al.* Corticosteroid injection in early treatment of lateral epicondylitis. *Clin J Sport Med* 2001;11:214–22.	Insufficient exercise data
Gunay Ucurum S, Kaya DO, Kayali Y, *et al.* Comparison of different electrotherapy methods and exercise therapy in shoulder impingement syndrome: A prospective randomized controlled trial. *Acta Orthop Traumatol Turc* 2018;52:249–55.	Insufficient exercise data
Pasin T, Ataoglu S, Pasin O, *et al.* Comparison of the effectiveness of platelet-rich plasma, corticosteroid, and physical therapy in subacromial impingement syndrome. *Arch Rheumatol* 2019;34:308–16.	Insufficient exercise data
Farfaras S, Sernert N, Hallström E, *et al.* Comparison of open acromioplasty, arthroscopic acromioplasty and physiotherapy in patients with subacromial impingement syndrome: a prospective randomised study. *Knee Surgery, Sport Traumatol Arthrosc* 2016;24:2181–91.	Insufficient exercise data
Subaşı V, Çakır T, Arıca Z, *et al.* Comparison of efficacy of kinesiological taping and subacromial injection therapy in subacromial impingement syndrome. *Clin Rheumatol* 2016;35:741–6.	Insufficient exercise data
Worsley P, Mottram S, Warner M, *et al.* Clinical outcomes following motor control rehabilitation for shoulder impingement. *Rheumatology* 2012;51:95.	Insufficient exercise data
Yelland M, Rabago D, Ryan M, *et al.* Prolotherapy injections and physiotherapy used singly and in combination for lateral epicondylalgia: a single-blinded randomised clinical trial. *BMC Musculoskelet Disord* 2019;20:509.	Insufficient exercise data
Wilson JK, Sevier TL, Helfst R, *et al.* Comparison of rehabilitation methods in the treatment of patellar tendinitis. *J Sport Rehabil* 2000;9:304–14.	Insufficient exercise data
Akkurt HE, Kocabas H, Yilmaz H, *et al.* Comparison of an epicondylitis bandage with a wrist orthosis in patients with lateral epicondylitis. *Prosthet Orthot Int* 2018;42:599–605.	Insufficient exercise data
Miccinilli S, Bravi M, Morrone M, *et al.* A Triple Application of Kinesio Taping Supports Rehabilitation Program for Rotator Cuff Tendinopathy: a Randomized Controlled Trial. *Ortop Traumatol Rehabil* 2018;20:499–505.	Insufficient exercise data
Struyf F, Nijs J, Mollekens S, *et al.* Scapular-focused treatment in patients with shoulder impingement syndrome: a randomized clinical trial. *Clin Rheumatol* 2013;32:73–85.	Insufficient exercise data

Haahr JP, Andersen JH. Prognostic factors in lateral epicondylitis: A randomized trial with one-year follow-up in 266 new cases treated with minimal occupational intervention of the usual approach in general practice. *Rheumatology* 2003;42:1216–25.	Insufficient exercise data
Dickens VA, Williams JL, Bhamra MS. Role of physiotherapy in the treatment of subacromial impingement syndrome: a prospective study. *Physiotherapy* 2005;91:159–64.	Insufficient exercise data
Wiener M, Mayer F. Effects of physiotherapy on peak torque and pain in patients with tendinitis of the supraspinatus muscle. *Dtsch Z Sportmed* 2005;56:383–7.	Insufficient exercise data
Brown R, Orchard J, Kinchington M, *et al*. Aprotinin in the management of Achilles tendinopathy: a randomised controlled trial. *Br J Sports Med* 2006;40:275–9.	Insufficient exercise data
Giombini A, Di Cesare A, Safran MR, *et al.* Short-term Effectiveness of Hyperthermia for Supraspinatus Tendinopathy in Athletes. *Am J Sports Med* 2006;34:1247–53.	Insufficient exercise data
Kaya E, Zinnuroglu M, Tugcu I. Kinesio taping compared to physical therapy modalities for the treatment of shoulder impingement syndrome. *Clin Rheumatol* 2011;30:201–7.	Insufficient exercise data
Dilek B, Gulbahar S, Gundogdu M, *et al.* Efficacy of Proprioceptive Exercises in Patients with Subacromial Impingement Syndrome: A Single-Blinded Randomized Controlled Study. *Am J Phys Med Rehabil* 2016;95:169–82.	Insufficient exercise data
Bisset L, Beller E, Jull G, *et al.* Mobilisation with movement and exercise, corticosteroid injection, or wait and see for tennis elbow: Randomised trial. *Br Med J* 2006;333:939–41.	Insufficient exercise data
van der Plas A, de Jonge S, de Vos RJ, *et al.* A 5-year follow-up study of Alfredson's heel-drop exercise programme in chronic midportion Achilles tendinopathy. *Br J Sport Med* 2012;46:214–8.	Insufficient exercise data
De Jonge S, de Vos RJ, Van Schie HTM, *et al.* One-year follow-up of a randamised controlled trial on added splinting to eccentric exercises in chronic midportion Achilles tendinopathy. *Br J Sports Med* 2010;44:673-677.	Insufficient exercise data
Ketola S, Lehtinen J, Rousi T, *et al.* Which patients do not recover from shoulder impingement syndrome, either with operative treatment or with nonoperative treatment? *Acta Orthop* 2015;86:641–6.	Insufficient exercise data
de Jonge S, de Vos J. R, Weir A, *et al.* One-year follow-up of platelet-rich plasma treatment in chronic Achilles tendinopathy: a double-blind randomized placebo-controlled trial. *Am J Sports Med* 2011;39:1623–9.	Insufficient exercise data
Ketola S, Lehtinen J, Elo P, *et al.* No difference in long-term development of rotator cuff rupture and muscle volumes in impingement patients with or without decompression: A randomized MRI study of 140 patients. *Acta Orthop* 2016;87:351–5.	Insufficient exercise data
Jowett S, Crawshaw DP, Helliwell PS, *et al.* Cost-effectiveness of exercise therapy after corticosteroid injection for moderate to severe shoulder pain due to subacromial impingement syndrome: a trial-based analysis. *Rheumatology* 2013;52:1485–91.	Insufficient exercise data
Struijs PAA, Korthals-de Bos IBC, van Tulder MW, *et al*. Cost effectiveness of brace, physiotherapy, or both for treatment of tennis elbow. *Br J Sports Med* 2006;40:637–43.	Insufficient exercise data

Haahr JP, Andersen JH. Exercises may be as efficient as subacromial decompression in patients with subacromial stage II impingement: 4-8-years' follow-up in a prospective, randomized study. *Scand J Rheumatol* 2006;35:224–8.	Insufficient exercise data
Ketola S, Lehtinen JT, Arnala I. Arthroscopic decompression not recommended in the treatment of rotator cuff tendinopathy. *Bone Joint J* 2017;99-B:799–805.	Insufficient exercise data
Coombes BK, Connelly L, Bisset L, *et al.* Economic evaluation favours physiotherapy but not corticosteroid injection as a first-line intervention for chronic lateral epicondylalgia: evidence from a randomised clinical trial. *Br J Sports Med* 2016;50:1400–5.	Insufficient exercise data
Abat F, Gelber PE, Polidori F, *et al.* 1 Clinical Results After EPI ® and Eccentric Exercise in Patellar Tendinopathy at 10 Years Follow-Up. *Br J Sports Med* 2014;48:A1. https://bjsm.bmj.com/lookup/doi/10.1136/bjsports-2014-094114.1 (accessed 12 Jun 2021).	Insufficient exercise data
Sosa C, Lorenzo A, Jimenez SL, *et al.* Eccentric exercise in treatment of patellar tendinopathy in high level basketball players. A randomised clinical trial [Abstract]. *J Strength Cond Res* 2014;28:1	Insufficient exercise data
Taunton JE, Ryan MB, Wong T. ECCENTRIC-ONLY HEEL DROP TRAINING: EXAMINING A DOSE RESPONSE IN PATIENTS WITH ACHILLES TENDINOSIS. *Clin J Sport Med* 2004;14:382-383.	Insufficient exercise data
Sandford FM, Sanders TA, Wilson H, *et al.* A randomised controlled trial of long-chain omega-3 polyunsaturated fatty acids in the management of rotator cuff related shoulder pain. *BMJ Open Sport Exerc Med* 2018;4:e000414.	Insufficient exercise data
Bisset L, Yelland M, Ryan M, *et al.* Testing the effectiveness of emerging injection therapies compared to physiotherapy for tennis elbow: a randomised control trial. *Physiotherapy* 2015;101:e155.	Insufficient exercise data
Stefanou A, Marshall N, Holdan W, *et al.* A randomized study comparing corticosteroid injection to corticosteroid iontophoresis for lateral epicondylitis. *J Hand Surg Am* 2012; 37:104-109.	Insufficient exercise data
Cherry E, Agostinucci J, McLinden J. The effect of cryotherapy and exercise on lateral epicondylitis: a controlled randomised study. *Int J Ther Rehabil* 2012;19:641-650.	Insufficient exercise data
Roddy E, Ogollah RO, Oppong R, Zwierska I, Datta P, Hall A, Hay E, Jackson S, Jowett S, Lewis M, Shufflebotham J. Optimising outcomes of exercise and corticosteroid injection in patients with subacromial pain (impingement) syndrome: a factorial randomised trial. *Br. J. Sports Med.* 2021;55:262-271.	Insufficient exercise data
Pearson SJ, Stadler S, Menz H, Morrissey D, Scott I, Munteanu S, Malliaras P. Immediate and short-term effects of short-and long-duration isometric contractions in patellar tendinopathy. *Clin J Sport Med.* 2020;30:335-340.	Insufficient exercise data
Bialoszewski D, Zaborowski G. Usefulness of Manual Therapy in the Rehabilitation of Patients with Chronic Rotator Cuff Injuries. Preliminary Report. *Ortop Traumatol Rehabil* 2011;1:9-20.	Not tendinopathy specific
Winters JC, Sobel JS, Groenier KH, *et al.* Comparison of physiotherapy, manipulation, and corticosteroid injection for treating shoulder complaints in general practice: randomised, single blind study. *BMJ* 1997;3:1320-1325.	Not tendinopathy specific
Brinks A, van Rijn RM, Willemsen SP, Bohnen AM, Verhaar JA, Koes BW, Bierma-Zeinstra SM. Corticosteroid injections for greater trochanteric pain syndrome: a randomized controlled trial in primary care. *Ann Fam Med*	Wrong concept

2011;1:226-34.	
Ebbesen BH, Mølgaard CM, Olesen JL, *et al.* No beneficial effect of polidocanol treatment in achilles tendinopathy: a randomised controlled trial. *Knee Surg Sports Traumatol Arthrosc* 2018;26:2038-44.	Wrong concept
Schmitt J, Haake M, Tosch A, *et al.* Low-energy extracorporeal shock-wave treatment (ESWT) for tendinitis of the supraspinatus: a prospective, randomised study. *J Bone Joint Surg Am* 2001;83:873-6.	Wrong concept
Speed CA, Richards C, Nichols D, *et al.* Extracorporeal shock-wave therapy for tendonitis of the rotator cuff: a double-blind, randomised, controlled trial. *J Bone Joint Surg Am* 2002;84:509-12.	Wrong concept
Alfredson H, Öhberg L. Sclerosing injections to areas of neo-vascularisation reduce pain in chronic Achilles tendinopathy: a double-blind randomised controlled trial. *Knee Surg Sports Traumatol Arthros*c 2005; 13:338-44.	Wrong concept
Furia JP. High-energy extracorporeal shock wave therapy as a treatment for chronic noninsertional Achilles tendinopathy. *Am J Sports Med* 2008;36:502-508.	Wrong concept
Gündüz R, Malas FÜ, Borman P, *et al.* Physical therapy, corticosteroid injection, and extracorporeal shock wave treatment in lateral epicondylitis. *Clin Rheumatol* 2012; 1:807-12.	Wrong concept
Schmitt J, Tosch A, Hünerkopf M, *et al.* Extracorporeal shockwave therapy (ESWT) as therapeutic option in supraspinatus tendon syndrome? One year results of a placebo controlled study. *Orthopade* 2002;1:652-7.	Wrong concept
Furia JP. Extracorporeal shockwave therapy in the treatment of chronic insertional Achilles tendinopathy: A congress report. Orthopade 2005;34:571-578.	Wrong concept
Skorupska E, Lisinski P, Samborski W. The effectiveness of the conservative versus myofascial pain physiotherapy in tennis elbow patients: double-blind randomized trial of 80 patients. *J Musculoskelet Pain* 2012; 1:41-50.	Wrong concept
Speed CA, Richards C, Nichols D, *et al.* Extracorporeal shock-wave therapy for tendonitis of the rotator cuff: a double-blind, randomised, controlled trial. *J Bone Surg Joint Am* 2002;84:509-512.	Wrong concept
Hoksrud A, Öhberg L, Alfredson H, *et al.* Ultrasound-guided sclerosis of neovessels in painful chronic patellar tendinopathy: a randomized controlled trial. *Am J Sports Med* 2006;34:1738-1746.	Wrong concept
Zwerver J, Hartgens F, Verhagen E, *et al.* No effect of extracorporeal shockwave therapy on patellar tendinopathy in jumping athletes during the competitive season: a randomized clinical trial. *Am J Sports Med* 2011;39:1191-1199.	Wrong concept
Saunders L. Laser versus ultrasound in the treatment of supraspinatus tendinosis: randomised controlled trial. *Physiotherapy* 2003;1:365-373.	Wrong concept
Eslamian F, Shakouri SK, Ghojazadeh M, *et al.* Effects of low-level laser therapy in combination with physiotherapy in the management of rotator cuff tendinitis. *Lasers Med Sci* 2012; 27:951-8.	Wrong HDI rank
Kumar N, Nehru A, Rajalakshmi D. Effect of taping as a component of conservative treatment for subacromial impingement syndrome. *Health* 2012; 26:237-41.	Wrong HDI rank

Martins LV, Marziale MH. Assessment of proprioceptive exercises in the treatment of rotator cuff disorders in nursing professionals: a randomized controlled clinical trial. *Braz J Phys Ther* 2012;16:502-9.	Wrong HDI rank
Moezy A, Sepehrifar S, Dodaran MS. The effects of scapular stabilization based exercise therapy on pain, posture, flexibility and shoulder mobility in patients with shoulder impingement syndrome: a controlled randomized clinical trial. *Med J Islam Repub Iran* 2014;28:87.	Wrong HDI rank
Letafatkar A, Rabiei P, Kazempour S, *et al.* Comparing the effects of no intervention with therapeutic exercise, and exercise with additional Kinesio tape in patients with shoulder impingement syndrome. A three-arm randomized controlled trial. *Clin Rehabil* 2021;35:558-567.	Wrong HDI rank
Shakeri H, Keshavarz R, Arab AM, *et al.* A randomized clinical trial of Kinesio-taping on DASH in patients with subacromial impingement syndrome. *J Nov Physiother* 2013;3:169.	Wrong HDI rank
Behera P, Dhillon M, Aggarwal S, *et al.* Leukocyte-poor platelet-rich plasma versus bupivacaine for recalcitrant lateral epicondylar tendinopathy. *J Orthop Surg* 2015;23:6-10.	Wrong HDI rank
Jiang W, Zhuang J, Zhang Y, *et al.* The effect of platelet-rich plasma in the treatment of external humeral epicondylitis and an analysis of the influencing factors. *Int J Clin Exp Med* 2020;13:3866-3874.	Wrong HDI rank
Martins LV, Marziale MHP. Assessment of proprioceptive exercises in the treatment of rotator cuff disorders in nursing professionals: a randomized controlled clinical trial. *Rev Bras Fisioter* 2012;16:502-509.	Wrong HDI rank
Moslehi M, Letafatkar A, Miri H. Feedback improves the scapular-focused treatment effects in patients with shoulder impingement syndrome. *Knee Surg Sports Traumatol Arthrosc* 2021;29:2281-2288.	Wrong HDI rank
Akhtar M, Karimi H, Gilani SA, *et al.* Effects of routine physiotherapy with and without neuromobilization in the management of internal shoulder impingement syndrome: A randomized controlled trial. *Pak J Med Sci* 2020;36:596-602	Wrong HDI rank
Babaei-Ghazani A, Shahrami B, Fallah E, *et al.* Continuous shortwave diathermy with exercise reduces pain and improves function in Lateral Epicondylitis more than sham diathermy: A randomized controlled trial. J *Bodyw Mov Ther* 2020; 1:69-76.	Wrong HDI rank
Martins da Silva L, Maciel Bello G, Chuaste Flores B, *et al.* Kinesio Tape In Shoulder Rotator Cuff Tendinopathy: A Randomized, Blind Clinical Trial. *Muscles Ligaments Tendons J* 2020; 10:364-375.	Wrong HDI rank
Kanniappan V, Sathosh AM. To Compare the Effect of Eccentric Exercises and Isometric Exercises for Achilles Tendinitis in Skaters. *Journal Lifestyle Med* 2020;10:49-54.	Wrong HDI rank
Kumar PG, Balamurugan N, Rajavel R, *et al.* Comparison between the effectiveness of decline squat exercise and forward lunges in athletes with patellar tendinopathy. *Drug Invent Today*. 2020;14:997-1000.	Wrong HDI rank
Ibrahim DH, El-Gazzar NM, El-Saadany HM, *et al.* Ultrasound-guided injection of platelet rich plasma versus corticosteroid for treatment of rotator cuff tendinopathy: effect on shoulder pain, disability, range of motion and ultrasonographic findings. *Egypt Rheumatol* 2019; 41:157-161.	Wrong HDI rank
Deshak S, Yeole U, Moralwar S. Effect of Functional Task Exercises on Hand Function and Grip Strength in Patients with Lateral epicondylitis. *Indian J Public*	Wrong HDI rank

Health Res Dev 2020;11:927-932.	
Bhardwaj P, Dhawan A. The relative efficacy of mobilization with movement versus Cyriax physiotherapy in the treatment of lateral epicondylitis. *Indian J Physiother Occup Ther* 2011;5:142-146.	Wrong HDI rank
Atya AM. Efficacy of microcurrent electrical stimulation on pain, proprioception accuracy and functional disability in subacromial impingement: RCT. *Indian J Physiother Occup Ther* 2012;6:15-18.	Wrong HDI rank
Mostafaee N, Divandari A, Negahban H, *et al.* Shoulder and scapula muscle training plus conventional physiotherapy versus conventional physiotherapy only: a randomized controlled trial of patients with lateral elbow tendinopathy. *Physiother Theory Pract* 2020;26:1-2.	Wrong HDI rank
Ramteke S, Samal S. To Study the Effect of Rotator Cuff Exercises on Tennis Elbow. *Indian J Public Health Res Dev* 2020;11:610-613.	Wrong HDI rank
Kuhkamar MMZ, Hadadnezhad M, Tazji MK. The effect of eight weeks' scapular focused training on pain, proprioception, scapular kinematics and upper extremity performance in male volleyball players with shoulder impingement syndrome: a randomized clinical trial study. *Med J Tabriz Uni Med* 2020;42:466-475.	Wrong HDI rank
Hölmich P, Uhrskou P, Ulnits L, *et al.* Effectiveness of active physical training as treatment for long-standing adductor-related groin pain in athletes: randomised trial. *Lancet* 1999; 6:439-43.	Wrong outcomes
De Reu S. The Immediate Effects of an External Rotation Exercise Program Compared with a General Exercise Program in Patients with Rotator Cuff Tendinopathy and Healthy Controls: a Randomised Controlled Trial (Doctoral dissertation, Ghent University).2018.1-41.	Wrong outcomes
Gatz M, Betsch M, Tingart M, *et al.* Effect of a 12-week Eccentric and Isometric Training in Achilles Tendinopathy on the Gastrocnemius Muscle: an Ultrasound Shear Wave Elastography Study. *Musles Ligaments Tendons J* 2020; 10:92-99.	Wrong outcomes
Romero-Morales C, Javier Martín-Llantino P, Calvo-Lobo C, *et al.* Ultrasonography effectiveness of the vibration vs cryotherapy added to an eccentric exercise protocol in patients with chronic mid-portion Achilles tendinopathy: A randomised clinical trial. *Int Wound J* 2019;16:542-549.	Wrong outcomes
Öhberg L, Alfredson H. Effects on neovascularisation behind the good results with eccentric training in chronic mid-portion Achilles tendinosis? *Knee Surg Sports Traumatol Arthrosc* 2004;12:465-470.	Wrong outcomes
Kim J, Lee SC, Chun Y, *et al.* Effects of a 4-Week Short-Foot Exercise Program on Gait Characteristics in Patients With Stage II Posterior Tibial Tendon Dysfunction. *J Sport Rehabil* 2020;30:120-128.	Wrong outcomes
Romero-Morales C, Martín-Llantino PJ, Calvo-Lobo C, *et al.* Effectiveness of eccentric exercise and a vibration or cryotherapy program in enhancing rectus abdominis muscle thickness and inter-rectus distance in patients with chronic mid-portion achilles tendinopathy: A randomized clinical trial. *Int J Med Sci* 2018;15:1764-1770.	Wrong outcomes
Gärdin A, Movin T, Svensson L, *et al.* The long-term clinical and MRI results following eccentric calf muscle training in chronic Achilles tendinosis. *Skeletal*	Wrong outcomes

Radiol 2010; 39:435-442.	
Stasinopoulos D, Stasinopoulos I. Comparison of effects of exercise programme, pulsed ultrasound and transverse friction in the treatment of chronic patellar tendinopathy. *Clin Rehabil* 2004;18:347-352.	Wrong outcomes
Knobloch K, Schreibmueller L, Longo UG, Vogt PM. Eccentric exercises for the management of tendinopathy of the main body of the Achilles tendon with or without the AirHeel™ Brace. A randomized controlled trial. A: effects on pain and microcirculation. *Disabil. Rehabil.* 2008;30:1685-91.	Wrong outcomes
Haahr JP, Østergaard S, Dalsgaard J, Norup K, Frost P, Lausen S, Holm EA, Andersen JH. Exercises versus arthroscopic decompression in patients with subacromial impingement: a randomised, controlled study in 90 cases with a one year follow up. *Ann Rheum Dis*. 2005;64:760-764.	Wrong outcomes
Senbursa G, Baltacı G, Atay A. Comparison of conservative treatment with and without manual physical therapy for patients with shoulder impingement syndrome: a prospective, randomized clinical trial. *Knee Surg Sports Traumatol Arthrosc*. 2007;15:915-921.	Wrong outcomes
Grymel-Kulesza E, Polak A, Kubacki J, Skrzypek-Poloczek B, Król P. The effect of a multi-modality therapy including active exercises, classic massage, cryotherapy and a combination of ultrasound and electrical stimulation on rotator cuff injuries. *Fizjoterapia Pol.* 2007;7:107-23.	Wrong outcomes
Yiasemides R, Halaki M, Cathers I, *et al.* Does passive mobilization of shoulder region joints provide additional benefit over advice and exercise alone for people who have shoulder pain and minimal movement restriction? A randomized controlled trial. *Phys Ther* 2011; 1:178-189.	Wrong population
Brumitt J, Hutchison MK, Kang D, *et al.* Blood flow restriction training for the rotator cuff: a randomized controlled trial. *Int J Sports Physiol Perform* 2020;19:1175-1180.	Wrong population
Tyler TF, Nicholas SJ, Schmitt BM, *et al.* Clinical outcomes of the addition of eccentrics for rehabilitation of previously failed treatments of golfers elbow. *Int J Sports Phys Ther* 2014;9:365-370	Wrong study design
Lyftogt J. Prolotherapy and Achilles tendinopathy: a prospective pilot study of an old treatment. *Australas Musculoskelet Med* 2005;10:17-19	Wrong study design
Holden S, Lyng K, Graven-Nielsen T, *et al.* Isometric exercise and pain in Patellar tendinopathy: a randomized crossover trial. *J Sci Med* 2020;1:208-14.	Wrong study design
Malliaras P, Cridland K, Hopmans R, *et al.* Internet and telerehabilitation-delivered management of rotator cuff–Related shoulder pain (INTEL trial): Randomized controlled pilot and feasibility trial. *JMIR mHealth uHealth* 2020;8:e24311.	Wrong study design
Miller P, Osmotherly P. Does scapula taping facilitate recovery for shoulder impingement symptoms? A pilot randomized controlled trial. *J Man Manip Ther* 2009; 1:6E-13E.	Wrong study design
Keene DJ, Soutakbar H, Hopewell S, *et al.* Development and implementation of the physiotherapy-led exercise interventions for the treatment of rotator cuff disorders for the 'Getting it Right: Addressing Shoulder Pain'(GRASP) trial. *Physiotherapy* 2020;1:252-266.	Wrong study design
Littlewood C, Malliaras P, Mawson S, *et al.* Development of a self-managed loaded exercise programme for rotator cuff tendinopathy. *Physiotherapy* 2013;	Wrong study design

1;99:358-362.	
van Rensburg KJ, Atkins E. Does thoracic manipulation increase shoulder range of movement in patients with subacromial impingement syndrome? A pilot study. *Int Musculoskelet Med* 2012; 1:101-107.	Wrong study design
Baeske R, Hall T, Silva MF. The inclusion of mobilisation with movement to a standard exercise programme for patients with rotator cuff related pain: a randomised, placebo-controlled protocol trial. *BMC Musculoskelet Disord* 2020;21:1-10.	Wrong study design
Davidson JH, Vandervoort A, Lessard L, *et al.* The effect of acupuncture versus ultrasound on pain level, grip strength and disability in individuals with lateral epicondylitis: a pilot study. *Physiother Can* 2001;53:195-202.	Wrong study design
Macías-Hernández SI, García-Morales JR, Hernández-Díaz C, *et al.* Tolerance and effectiveness of eccentric vs. concentric muscle strengthening in rotator cuff partial tears and moderate to severe shoulder pain. A randomized pilot study. *J Clin Orthop Trauma* 2021; 1:106-112.	Wrong study design
Wetke E, Johannsen F, Langberg H. A hilles tendinopathy: A prospective study on the effect of active rehabilitation and steroid injections in a clinical setting. *Scan J Med Sci Sports* 2015;25:e392-399.	Wrong study design
Payne C. Clinical applications of shear wave elastography to achilles tendon imaging and the monitoring of a rehabilitation protocol for achilles tendinopathy (Doctoral dissertation, University of Brighton). 2018.109-187.	Wrong study design
Lee DR, Kim LJ. Internal-and External-Rotation Peak Torque in Little League Baseball Players with Subacromial Impingement Syndrome: Improved by Closed Kinetic Chain Shoulder Training. *J Sport Rehabil* 2016;25:263-265.	Wrong study design
Bernhardsson S, Klintberg IH, Wendt GK. Evaluation of an exercise concept focusing on eccentric strength training of the rotator cuff for patients with subacromial impingement syndrome. *Clin Rehabil* 2011;25:69-78.	Wrong study design
Alfredson H, Lorentzon R. Intratendinous glutamate levels and eccentric training in chronic Achilles tendinosis: a prospective study using microdialysis technique. *Knee Surg Sports Traumatol Arthrosc* 2003;11(3):196-199.	Wrong study design
Silbernagel KG, Thomeé R, Eriksson BI, *et al.* Full symptomatic recovery does not ensure full recovery of muscle-tendon function in patients with Achilles tendinopathy. *Br J Sports Med* 2007;41:276-280.	Wrong study design
Desmeules F, Minville L, Riederer B, *et al.* Acromio-humeral distance variation measured by ultrasonography and its association with the outcome of rehabilitation for shoulder impingement syndrome. *Clin J Sport Med* 2004;14:197-205.	Wrong study design
Worsley P, Warner M, Mottram S, *et al.* Motor control retraining exercises for shoulder impingement: effects on function, muscle activation, and biomechanics in young adults. *J Shoulder Elbow Surg* 2013;22:e11-9.	Wrong study design
Røe C, Brox JI, Bøhmer AS, *et al.* Muscle activation after supervised exercises in patients with rotator tendinosis. *Arch Phys Med Rehabil* 2000;8:67-72.	Wrong study design
Valera-Garrido F, Minaya-Muñoz F, Medina-Mirapeix F. Ultrasound-guided percutaneous needle electrolysis in chronic lateral epicondylitis: short-term and long-term results. *Acupunct Med* 2014;32(6):446-454.	Wrong study design
Croisier JL, Forthomme B, Foidart-Dessalle M, *et al.* Treatment of recurrent tendinitis by isokinetic eccentric exercises. *Isokinet Exerc Sci* 2001;9:133-141.	Wrong study design

Kaux JF, Forthomme B, Namurois MH, *et al.* Description of a standardized rehabilitation program based on sub-maximal eccentric following a platelet-rich plasma infiltration for jumper's knee. *Muscles Ligaments Tendons J* 2014;4:85-89.	Wrong study design
Langberg H, Ellingsgaard H, Madsen T, *et al.* Eccentric rehabilitation exercise increases peritendinous type I collagen synthesis in humans with Achilles tendinosis. *Scand J Med Sci Sports* 2007 17:61-66.	Wrong study design
De Mey K, Danneels L, Cagnie B, *et al.* Scapular muscle rehabilitation exercises in overhead athletes with impingement symptoms: effect of a 6-week training program on muscle recruitment and functional outcome. *Am J Sports Med* 2012;40:1906-1915.	Wrong study design
Tumilty S, Baxter GD. Heavy load eccentric exercise for Achilles tendinopathy; too much of a good thing?. *Physiotherapy* 2015;101:e1546-1547.	Wrong study design
Abat F, Gelber PE, Polidori F, Monllau JC, Sanchez-Ibañez JM. Clinical results after ultrasound-guided intratissue percutaneous electrolysis (EPI®) and eccentric exercise in the treatment of patellar tendinopathy. *Knee Surg Sports Traumatol Arthrosc.* 2015;23:1046-52.	Wrong study design
Knobloch K. Eccentric training in Achilles tendinopathy: is it harmful to tendon microcirculation?. *Br. J. Sports Med.*2007;41:1-5.	Wrong study design
Maffulli N, Walley G, Sayana MK, Longo UG, Denaro V. Eccentric calf muscle training in athletic patients with Achilles tendinopathy. *Disabil. Rehabil.* 2008;30:1677-84.	Wrong study design
Sayana MK, Maffulli N. Eccentric calf muscle training in non-athletic patients with Achilles tendinopathy. *J Sci Med Sport*. 2007;10:52-8.	Wrong study design
Abat F, Diesel WJ, Gelber PE, Polidori F, Monllau JC, Sanchez-Ibañez JM. Effectiveness of the Intratissue Percutaneous Electrolysis (EPI®) technique and isoinertial eccentric exercise in the treatment of patellar tendinopathy at two years follow-up. *MLTJ.* 2014;4:188-193.	Wrong study design
Fahlström M, Jonsson P, Lorentzon R, Alfredson H. Chronic Achilles tendon pain treated with eccentric calf-muscle training. *Knee Surg Sports Traumatol Arthrosc.* 2003;11:327-33.	Wrong study design
Savoie A, Mercier C, Desmeules F, Frémont P, Roy JS. Effects of a movement training oriented rehabilitation program on symptoms, functional limitations and acromiohumeral distance in individuals with subacromial pain syndrome. *Man Ther.* 2015;20:703-8.	Wrong study design
Hakgüder A, Tastekin N, Birtane M, Uzunca K, Zateri C, Süt N. Comparison of the Short-Term Efficacy of Physical Therapy in Subacromial Impingement Syndrome Patients with Stage I and II Magnetic Resonance Imaging Findings. *Turk. J. Rheumatol.* 2011;26(2):127-134.	Wrong study design
Kachanathu SJ, Zedan AM, Hafez AR, Alodaibi FA, Alenazi AM, Nuhmani S. Effect of shoulder stability exercises on hand grip strength in patients with shoulder impingement syndrome. Somatosensory & motor research. 2019;36:97-101.	Wrong study design
Chen TW, Huei Su J, Lin TY, Lin CW, Chou PS. Effects of eccentric exercise and extracorporeal shock wave therapy on rehabilitation of patients with noncalcific rotator cuff tendinopathy. Clin Res Foot Ankle. 2017;5(222):2.	Not including exercise only treatment arm

Carey TS. Corticosteroid injection worsened recovery and recurrence of tennis elbow; physiotherapy had no effect. Annals of Internal Medicine. 2013 May;158(10):JC8.	Not including exercise only treatment arm
Tumilty S, Mani R, Baxter GD. Photobiomodulation and eccentric exercise for Achilles tendinopathy: a randomized controlled trial. Lasers in medical science. 2016 Jan;31(1):127-35.	Not including exercise only treatment arm
Faria AP, da Silva EB, Dantas EH. Os efeitos dos diferentes recursos fototerapêuticos sobre a dor em indivíduos portadores de síndrome do impacto do ombro. Fitness & performance journal. 2006(6):354-8.	Not including exercise only treatment arm
Smidt N, Van Der Windt DA, Assendelft WJ, Devillé WL, Korthals-de Bos IB, Bouter LM. Corticosteroid injections, physiotherapy, or a wait-and-see policy for lateral epicondylitis: a randomised controlled trial. The Lancet. 2002 Feb 23;359(9307):657-62.	Not including exercise only treatment arm
Pérez-Merino L, Casajuana MC, Bernal G, Faba J, Astilleros AE, González R, Giralt M, Romeu M, Nogués MR. Evaluation of the effectiveness of three physiotherapeutic treatments for subacromial impingement syndrome: a randomised clinical trial. Physiotherapy. 2016 Mar 1;102(1):57-63.	Not including exercise only treatment arm
Scott A, LaPrade RF, Harmon KG, Filardo G, Kon E, Della Villa S, Bahr R, Moksnes H, Torgalsen T, Lee J, Dragoo JL. Platelet-rich plasma for patellar tendinopathy: a randomized controlled trial of leukocyte-rich PRP or leukocyte-poor PRP versus saline. The American journal of sports medicine. 2019 Jun;47(7):1654-61.	Not including exercise only treatment arm
Mayer F, Hirschmüller A, Müller S, Schuberth M, Baur H. Effects of short-term treatment strategies over 4 weeks in Achilles tendinopathy. British journal of sports medicine. 2007 Jul 1;41(7):e6-.	Not including exercise only treatment arm
Lee S, Ko Y, Lee W. Changes in pain, dysfunction, and grip strength of patients with acute lateral epicondylitis caused by frequency of physical therapy: a randomized controlled trial. Journal of physical therapy science. 2014;26(7):1037-40.	Not including exercise only treatment arm
Olaussen M, Holmedal Ø, Mdala I, Brage S, Lindbæk M. Corticosteroid or placebo injection combined with deep transverse friction massage, Mills manipulation, stretching and eccentric exercise for acute lateral epicondylitis: a randomised, controlled trial. BMC músculoskeletal disorders. 2015 Dec;16(1):1-3.	Not including exercise only treatment arm
Land H, Gordon S, Watt K. Effect of manual physiotherapy in homogeneous individuals with subacromial shoulder impingement: a randomized controlled trial. Physiotherapy research international. 2019 Apr;24(2):e1768.	Not including exercise only treatment arm
Kulig K, Reischl SF, Pomrantz AB, Burnfield JM, Mais-Requejo S, Thordarson DB, Smith RW. Nonsurgical management of posterior tibial tendon dysfunction with orthoses and resistive exercise: a randomized controlled trial. Physical Therapy. 2009 Jan 1;89(1):26-37.	Not including exercise only treatment arm
Eraslan L, Yuce D, Erbilici A, Baltaci G. Does Kinesiotaping improve pain and functionality in patients with newly diagnosed lateral epicondylitis?. Knee Surgery, Sports Traumatology, Arthroscopy. 2018 Mar;26(3):938-45.	Not including exercise only treatment arm
García I, Lobo C, López E, Serván JL, Tenías JM. Comparative effectiveness of ultrasonophoresis and iontophoresis in impingement syndrome: a double-blind, randomized, placebo controlled trial. Clinical rehabilitation. 2016 Apr;30(4):347-58.	Not including exercise only treatment arm

Boesen AP, Hansen R, Boesen MI, Malliaras P, Langberg H. Effect of high-volume injection, platelet-rich plasma, and sham treatment in chronic midportion Achilles tendinopathy: a randomized double-blinded prospective study. The American journal of sports medicine. 2017 Jul;45(9):2034-43.	Not including exercise only treatment arm
Struijs PA, Kerkhoffs GM, Assendelft WJ, van Dijk CN. Conservative treatment of lateral epicondylitis: brace versus physical therapy or a combination of both—a randomized clinical trial. The American journal of sports medicine. 2004 Mar;32(2):462-9.	Not including exercise only treatment arm
Yuruk ZO, Kirdi N, editors. The effects of radial extracorporeal shock wave therapy on subjective pain and functionality in patients with lateral epicondylitis. Fizyoterapi Rehabilitasyon Conference: 15th Congress of Advances in Physiotherapy; 2014; Ankara Turkey: Turkish Physical Therapy Association.	Not including exercise only treatment arm
Nazligul T, Akpinar P, Aktas I, Hartevioglu C. The effect of interferential current therapy on patients with subacromial impingement syndrome: a randomized, double-blind, sham-controlled study. European Journal of Physical and Rehabilitation Medicine. 2017 Sep 11;54(3):351-7.	Not including exercise only treatment arm
Ellegaard K, Christensen R, Rosager S, Bartholdy C, Torp-Pedersen S, Bandholm T, Danneskiold-Samsøe B, Bliddal H, Henriksen M. Exercise therapy after ultrasound-guided corticosteroid injections in patients with subacromial pain syndrome: a randomized controlled trial. Arthritis research & therapy. 2016 Dec;18(1):1-9.	Not including exercise only treatment arm
Dogan SK, Saime AY, Evcik D. The effectiveness of low laser therapy in subacromial impingement syndrome: a randomized placebo controlled double-blind prospective study. Clinics. 2010 Jan 1;65(10):1019-22.	Not including exercise only treatment arm
Røe C, Ødegaard TT, Hilde F, Maehlum S, Halvorsen T. No effect of supplement of essential fatty acids on lateral epicondylitis. Tidsskrift for den Norske Laegeforening: Tidsskrift for Praktisk Medicin, ny Raekke. 2005 Oct 1;125(19):2615-8.	Not including exercise only treatment arm
Conroy DE, Hayes KW. The effect of joint mobilization as a component of comprehensive treatment for primary shoulder impingement syndrome. Journal of Orthopaedic & Sports Physical Therapy. 1998 Jul;28(1):3-14.	Not including exercise only treatment arm
Celik D, Atalar AC, Guclu A, Demirhan M. The contribution of subacromial injection to the conservative treatment of impingement syndrome. Acta Orthop Traumatol Turc. 2009 Aug 1;43(4):331-5.	Not including exercise only treatment arm
Chung B, Wiley JP. Effectiveness of extracorporeal shock wave therapy in the treatment of previously untreated lateral epicondylitis: a randomized controlled trial. The American journal of sports medicine. 2004 Oct;32(7):1660-7.	Not including exercise only treatment arm
Nilgun BE, Simsek I, Suat ER, Yakut Y, Uygur F. Home-based general versus center-based selective rehabilitation in patients with posterior tibial tendon dysfunction. Acta orthopaedica et traumatologica turcica. 2012 Jan 1;46(4):286-92.	Not including exercise only treatment arm
Houck J, Neville C, Tome J, Flemister A. Randomized controlled trial comparing orthosis augmented by either stretching or stretching and strengthening for stage II tibialis posterior tendon dysfunction. Foot & ankle international. 2015 Sep;36(9):1006-16.	Not including exercise only treatment arm
Celik D, Akyuz G, Yeldan I. Comparison of the effects of two different exercise programs on pain in subacromial impingement syndrome. Acta Orthop Traumatol Turc. 2009 Jan 1;43(6):504-9.	Not including exercise only treatment arm

Yeldan I, Cetin E, Razak Ozdincler A. The effectiveness of low-level laser therapy on shoulder function in subacromial impingement syndrome. Disability and rehabilitation. 2009 Jan 1;31(11):935-40.	Not including exercise only treatment arm
Ingwersen KG, Jensen SL, Sørensen L, Jørgensen HR, Christensen R, Søgaard K, Juul-Kristensen B. Three months of progressive high-load versus traditional low-load strength training among patients with rotator cuff tendinopathy: primary results from the double-blind randomized controlled RoCTEx trial. Orthopaedic journal of sports medicine. 2017 Aug 23;5(8):2325967117723292.	Not including exercise only treatment arm
Chary-Valckenaere I, Loeuille D, Jay N, Kohler F, Tamisier JN, Roques CF, Boulange M, Gay G. Spa therapy together with supervised self-mobilisation improves pain, function and quality of life in patients with chronic shoulder pain: a single-blind randomised controlled trial. International journal of biometeorology. 2018 Jun;62(6):1003-14.	Not including exercise only treatment arm
Cacchio A, Rompe JD, Furia JP, Susi P, Santilli V, De Paulis F. Shockwave therapy for the treatment of chronic proximal hamstring tendinopathy in professional athletes. The American journal of sports medicine. 2011 Jan;39(1):146-53.	Not including exercise only treatment arm
Kedia M, Williams M, Jain L, Barron M, Bird N, Blackwell B, Richardson DR, Ishikawa S, Murphy GA. The effects of conventional physical therapy and eccentric strengthening for insertional achilles tendinopathy. International journal of sports physical therapy. 2014 Aug;9(4):488.	Not including exercise only treatment arm
Warden SJ, Metcalf BR, Kiss ZS, Cook JL, Purdam CR, Bennell KL, Crossley KM. Low-intensity pulsed ultrasound for chronic patellar tendinopathy: a randomized, double-blind, placebo-controlled trial. Rheumatology. 2008 Apr 1;47(4):467-71.	Not including exercise only treatment arm
Celik D, Anaforoglu Kulunkoglu B. Photobiomodulation therapy versus extracorporeal shock wave therapy in the treatment of lateral epicondylitis. Photobiomodulation, photomedicine, and laser surgery. 2019 May 1;37(5):269-75.	Not including exercise only treatment arm
Clarke AW, Alyas F, Morris T, Robertson CJ, Bell J, Connell DA. Skin-derived tenocyte-like cells for the treatment of patellar tendinopathy. The American journal of sports medicine. 2011 Mar;39(3):614-23.	Not including exercise only treatment arm
Bell KJ, Fulcher ML, Rowlands DS, Kerse N. Impact of autologous blood injections in treatment of mid-portion Achilles tendinopathy: double blind randomised controlled trial. Bmj. 2013 Apr 18;346.	Not including exercise only treatment arm
Boesen AP, Langberg H, Hansen R, Malliaras P, Boesen MI. High volume injection with and without corticosteroid in chronic midportion achilles tendinopathy. Scandinavian Journal of Medicine & Science in Sports. 2019 Aug;29(8):1223-31.	Not including exercise only treatment arm
Yazmalar L, Sarıyıldız MA, Batmaz İ, Alpaycı M, Burkan YK, Özkan Y, Okçu M, Çevik R. Efficiency of therapeutic ultrasound on pain, disability, anxiety, depression, sleep and quality of life in patients with subacromial impingement syndrome: A randomized controlled study. Journal of Back and Musculoskeletal Rehabilitation. 2016 Jan 1;29(4):801-7.	Not including exercise only treatment arm
Bennell K, Wee E, Coburn S, Green S, Harris A, Staples M, Forbes A, Buchbinder R. Efficacy of standardised manual therapy and home exercise programme for chronic rotator cuff disease: randomised placebo controlled trial. Bmj. 2010 Jun 8;340.	Not including exercise only treatment arm

Söderberg J, Grooten WJ, Äng BO. Effects of eccentric training on hand strength in subjects with lateral epicondylalgia: a randomized-controlled trial. Scandinavian journal of medicine & science in sports. 2012 Dec;22(6):797-803.	Not including exercise only treatment arm
Kang FJ, Chiu YC, Wu SC, Wang TG, Yang JL, Lin JJ. Kinesiology taping with exercise does not provide additional improvement in round shoulder subjects with impingement syndrome: a single-blinded randomized controlled trial. Physical Therapy in Sport. 2019 Nov 1;40:99-106.	Not including exercise only treatment arm
De Vos RJ, Weir A, van Schie HT, Bierma-Zeinstra SM, Verhaar JA, Weinans H, Tol JL. Platelet-rich plasma injection for chronic Achilles tendinopathy: a randomized controlled trial. Jama. 2010 Jan 13;303(2):144-9.	Not including exercise only treatment arm
Santamato A, Panza F, Notarnicola A, Cassatella G, Fortunato F, De Sanctis JL, Valeno G, Kehoe PG, Seripa D, Logroscino G, Fiore P. Is extracorporeal shockwave therapy combined with isokinetic exercise more effective than extracorporeal shockwave therapy alone for subacromial impingement syndrome? A randomized clinical trial. Journal of Orthopaedic & Sports Physical Therapy. 2016 Sep;46(9):714-25.	Not including exercise only treatment arm
Holmgren T, Hallgren HB, Öberg B, Adolfsson L, Johansson K. Effect of specific exercise strategy on need for surgery in patients with subacromial impingement syndrome: randomised controlled study. Bmj. 2012 Feb 20;344.	Not including exercise only treatment arm
Menek B, Algun ZC, Tarakçı D. Effectiveness of mulligan mobilization on range of motion and function in individuals with subacromial impingement syndrome. InWCO-IOF-ESCEO World Congress on Osteoporosis, Osteoarthritis and Musculoskeletal Diseases 2018. Springer London Ltd.	Not including exercise only treatment arm
Ilhanli I, Guder N, Gul M. Platelet-rich plasma treatment with physical therapy in chronic partial supraspinatus tears. Iranian Red Crescent Medical Journal. 2015 Sep;17(9).	Not including exercise only treatment arm
Thijs KM, Zwerver J, Backx FJ, Steeneken V, Rayer S, Groenenboom P, Moen MH. Effectiveness of shockwave treatment combined with eccentric training for patellar tendinopathy: a double-blinded randomized study. Clinical journal of sport medicine. 2017 Mar 1;27(2):89-96.	Not including exercise only treatment arm
Aktas I, Akgun K, Cakmak B. Therapeutic effect of pulsed electromagnetic field in conservative treatment of subacromial impingement syndrome. Clinical rheumatology. 2007 Aug;26(8):1234-9.	Not including exercise only treatment arm
Gedrimė DJ, Gedrimas D, Karpavičienė A, Skurvydas A. Effect of Visual and Auditory Feedback Exercises on Shoulder Function in Rotator Cuff Tendonitis Patients. Baltic Journal of Sport and Health Sciences. 2018 Jun 25;2(109).	Not including exercise only treatment arm
Kachanathu SJ, Alenazi AM, Hafez AR, Algarni AD, Alsubiheen AM. Comparison of the effects of short-duration wrist joint splinting combined with physical therapy and physical therapy alone on the management of patients with lateral epicondylitis. European journal of physical and rehabilitation medicine. 2019 Mar 21;55(4):488-93.	Not including exercise only treatment arm
Belley AF, Mercier C, Bastien M, Léonard G, Gaudreault N, Roy JS. Anodal transcranial direct-current stimulation to enhance rehabilitation in individuals with rotator cuff tendinopathy: a triple-blind randomized controlled trial. journal of orthopaedic & sports physical therapy. 2018 Jul;48(7):541-51.	Not including exercise only treatment arm
Abat F, Sánchez-Sánchez JL, Martín-Nogueras AM, Calvo-Arenillas JI, Yajeya J, Méndez-Sánchez R, Monllau JC, Gelber PE. Randomized controlled trial comparing the effectiveness of the ultrasound-guided galvanic electrolysis	Not including exercise only treatment arm

technique (USGET) versus conventional electro-physiotherapeutic treatment on patellar tendinopathy. Journal of experimental orthopaedics. 2016 Dec;3(1):1-8.	
Stasinopoulos D, Stasinopoulos I, Pantelis M, Stasinopoulou K. Comparing the effects of exercise program and low-level laser therapy with exercise program and polarized polychromatic non-coherent light (bioptron light) on the treatment of lateral elbow tendinopathy. Photomedicine and laser surgery. 2009 Jun 1;27(3):513-20.	Not including exercise only treatment arm
Stergioulas A. Effects of low-level laser and plyometric exercises in the treatment of lateral epicondylitis. Photomedicine and laser surgery. 2007 Jun 1;25(3):205-13.	Not including exercise only treatment arm
Rosety-Rodríguez M, Ordóñez-Muñoz FJ, Huesa-Jiménez F, Rosety Rodriguez J, Gómez-Rodríguez F, Rosety-Plaza M. Actualización del trabajo excéntrico de cuádriceps en pacientes en edad laboral con tendinopatía rotuliana. Patología del aparato locomotor. 2006 Jun;4(2):105-7.	Not including exercise only treatment arm
Akyol Y, Ulus Y, Durmus D, Canturk F, Bilgici A, Kuru O, Bek Y. Effectiveness of microwave diathermy on pain, functional capacity, muscle strength, quality of life, and depression in patients with subacromial impingement syndrome: a randomized placebo-controlled clinical study. Rheumatology international. 2012 Oct;32(10):3007-16.	Not including exercise only treatment arm
Tumilty S, McDonough S, Hurley DA, Baxter GD. Clinical effectiveness of low-level laser therapy as an adjunct to eccentric exercise for the treatment of Achilles' tendinopathy: a randomized controlled trial. Archives of physical medicine and rehabilitation. 2012 May 1;93(5):733-9.	Not including exercise only treatment arm
Stergioulas A, Stergioula M, Aarskog R, Lopes-Martins RA, Bjordal JM. Effects of low-level laser therapy and eccentric exercises in the treatment of recreational athletes with chronic achilles tendinopathy. The American journal of sports medicine. 2008 May;36(5):881-7.	Not including exercise only treatment arm
Notarnicola A, Maccagnano G, Tafuri S, Forcignanò MI, Panella A, Moretti B. CHELT therapy in the treatment of chronic insertional Achilles tendinopathy. Lasers in Medical Science. 2014 May;29(3):1217-25.	Not including exercise only treatment arm
Lee WC, Ng GY, Zhang ZJ, Malliaras P, Masci L, Fu SN. Changes on tendon stiffness and clinical outcomes in athletes are associated with patellar tendinopathy after eccentric exercise. Clinical Journal of Sport Medicine. 2020 Jan 1;30(1):25-32.	Not including exercise only treatment arm
Bağcıer F, Yılmaz N. The Impact of Extracorporeal Shock Wave Therapy and Dry Needling Combination on the Pain, Grip Strength and Functionality in Patients Diagnosed with Lateral Epicondylitis. Turkish Journal of Osteoporosis/Turk Osteoporoz Dergisi. 2019 Aug 1;25(2).	Not including exercise only treatment arm
Croisier JL, Foidart-Dessalle M, Tinant F, Crielaard JM, Forthomme B. An isokinetic eccentric programme for the management of chronic lateral epicondylar tendinopathy. British journal of sports medicine. 2007 Apr 1;41(4):269-75.	Not including exercise only treatment arm
Tyler TF, Thomas GC, Nicholas SJ, McHugh MP. Addition of isolated wrist extensor eccentric exercise to standard treatment for chronic lateral epicondylosis: a prospective randomized trial. Journal of Shoulder and Elbow surgery. 2010 Sep 1;19(6):917-22.	Not including exercise only treatment arm
Gursel YK, Ulus Y, Bilgic A, Dincer G, van der Heijden GJ. Adding ultrasound in the management of soft tissue disorders of the shoulder: a randomized	Not including exercise only treatment arm

placebo-controlled trial. Physical therapy. 2004 Apr 1;84(4):336-43.	
de Miguel Valtierra L, Moreno JS, Fernández-de-Las-Peñas C, Cleland JA, Arias-Buría JL. Ultrasound-guided application of percutaneous electrolysis as an adjunct to exercise and manual therapy for subacromial pain syndrome: A randomized clinical trial. The Journal of Pain. 2018 Oct 1;19(10):1201-10.	Not including exercise only treatment arm
EKEN GEDİK D, DOST SÜRÜCÜ G, YILDIRIM A, KARABİBER M. LATERAL EPİKONDİLİT TEDAVİSİNDE OTOLOG KAN ENJEKSİYONUNUN ETKİNLİĞİ: RANDOMİZE KLİNİK ÇALIŞMA. Duzce Medical Journal. 2016 Jan 1;18(1).	Not including exercise only treatment arm
Rhon DI, Boyles RB, Cleland JA. One-year outcome of subacromial corticosteroid injection compared with manual physical therapy for the management of the unilateral shoulder impingement syndrome: a pragmatic randomized trial. Annals of internal medicine. 2014 Aug 5;161(3):161-9.	Not including exercise only treatment arm
van der Vlist AC, van Oosterom RF, van Veldhoven PL, Bierma-Zeinstra SM, Waarsing JH, Verhaar JA, de Vos RJ. Effectiveness of a high volume injection as treatment for chronic Achilles tendinopathy: randomised controlled trial. bmj. 2020 Sep 9;370.	Not including exercise only treatment arm
McGee C, Kersting E, Palmer-McLean K, Davies GJ. Standard rehabilitation vs standard plus closed kinetic chain rehabilitation for patients with shoulder impingement: A rehabilitation outcomes study. Physical Therapy. 1999;79.	Not including exercise only treatment arm
Cha JY, Kim JH, Hong J, Choi YT, Kim MH, Cho JH, Ko IG, Jee YS. A 12-week rehabilitation program improves body composition, pain sensation, and internal/external torques of baseball pitchers with shoulder impingement symptom. Journal of exercise rehabilitation. 2014 Feb;10(1):35.	Not including exercise only treatment arm
Rodríguez-Huguet M, Góngora-Rodríguez J, Rodríguez-Huguet P, Ibañez-Vera AJ, Rodríguez-Almagro D, Martín-Valero R, Díaz-Fernández Á, Lomas-Vega R. Effectiveness of percutaneous electrolysis in supraspinatus tendinopathy: A single-blinded randomized controlled trial. Journal of Clinical Medicine. 2020 Jun 12;9(6):1837.	Not including exercise only treatment arm
Rodríguez-Huguet M, Góngora-Rodríguez J, Lomas-Vega R, Martín-Valero R, Díaz-Fernández Á, Obrero-Gaitán E, Ibáñez-Vera AJ, Rodríguez-Almagro D. Percutaneous electrolysis in the treatment of lateral epicondylalgia: A single-blind randomized controlled trial. Journal of Clinical Medicine. 2020 Jul 1;9(7):2068.	Not including exercise only treatment arm
Ramon S, Russo S, Santoboni F, Lucenteforte G, Di Luise C, de Unzurrunzaga R, Vetrano M, Albano M, Baldini R, Cugat R, Stella G. Focused shockwave treatment for greater trochanteric pain syndrome: a multicenter, randomized, controlled clinical trial. JBJS. 2020 Aug 5;102(15):1305-11.	Not including exercise only treatment arm
Buyuksireci DE, Turk AC. Evaluation of the effectiveness of dexamethasone iontophoresis in patients with subacromial impingement syndrome. Journal of Orthopaedic Science. 2021 Sep 1;26(5):786-91.	Not including exercise only treatment arm
Beaudreuil J, Lasbleiz S, Yelnik A, Bardin T, Orcel P. Effect of dynamic humeral centering on painful active elevation of the arm in subacromial impingement syndrome: A randomized trial. Annals of Physical and Rehabilitation Medicine. 2012(55):e161.	Not including exercise only treatment arm
Pekgöz F, Taşkıran H, Mutlu EK, Atalay A, Çeliker R. Comparison of mobilization with supervised exercise for patients with subacromial impingement	Not including exercise only treatment arm

syndrome. Turkish journal of physical medicine and rehabilitation. 2020 Jun;66(2):184.	
McQueen KS, Powell RK, Keener T, Whalley R, Calfee RP. Role of strengthening during nonoperative treatment of lateral epicondyle tendinopathy. Journal of Hand Therapy. 2021 Oct 1;34(4):619-26.	Not including exercise only treatment arm
Koç C, Kurt EE, Koçak FA, Erdem HR, Konar NM. Does balneotherapy provide additive effects to physical therapy in patients with subacute supraspinatus tendinopathy? A randomized, controlled, single-blind study. International Journal of Biometeorology. 2021 Feb;65(2):301-10.	Not including exercise only treatment arm
Lee WC. The mechanical, physiological and therapeutic effects of eccentric exercise combined with extracorporeal shockwave therapy in athletes with patellar tendinopathy. 2017.	Not including exercise only treatment arm
Askling CM, Tengvar M, Tarassova O, Thorstensson A. Acute hamstring injuries in Swedish elite sprinters and jumpers: a prospective randomised controlled clinical trial comparing two rehabilitation protocols. British journal of sports medicine. 2014 Apr 1;48(7):532-9.	Not including required tendinopathies
Jeong TH, Oh JK, Lee HJ, Yang YJ, Nha KW, Suh JS. The effect of the combined stretching and strengthening exercise on the clinical symptoms in posterior tibial tendon dysfunction patient. Journal of Korean Foot and Ankle Society. 2008;12(1):47-54.	Not including required tendinopathies
Genç E, Duymaz T. Effectiveness of kinesio taping in bicipital tendinitis treatment: A randomized controlled trial. Annals of Clinical and Analytical Medicine. 2020.	Not including required tendinopathies
Ginn K, Cohen M. Exercise therapy for shoulder pain aimed at restoring neuromuscular control: a randomized comparative clinical trial. Journal of Rehabilitation Medicine. 2005 Mar 1;37(2):115-22.	Wrong outcomes
Østerås H, Arild Torstensen T, Arntzen G, S Østerås B. A comparison of work absence periods and the associated costs for two different modes of exercise therapies for patients with longstanding subacromial pain. Journal of Medical Economics. 2008 Jan 1;11(3):371-81.	Wrong outcomes
Østerås H, Torstensen TA, Haugerud L, Østerås BS. Dose–response effects of graded therapeutic exercises in patients with long-standing subacromial pain. Advances in physiotherapy. 2009 Jan 1;11(4):199-209.	Insufficient data
Ganderton C, Semciw A, Cook J, Moreira E, Pizzari T. Gluteal loading versus sham exercises to improve pain and dysfunction in postmenopausal women with greater trochanteric pain syndrome: a randomized controlled trial. Journal of Women's Health. 2018 Jun 1;27(6):815-29.	Insufficient data
Grävare Silbernagel K, Crossley KM. A proposed return-to-sport program for patients with midportion Achilles tendinopathy: rationale and implementation. journal of orthopaedic & sports physical therapy. 2015 Nov;45(11):876-86.	Insufficient data
Speed CA, Richards C, Nichols D, Burnet S, Wies JT, Humphreys H, Hazleman BL. Extracorporeal shock-wave therapy for tendonitis of the rotator cuff: a double-blind, randomised, controlled trial. The Journal of Bone and Joint Surgery. British volume. 2002 May;84(4):509-12.	Insufficient data
Tonks JH. Evaluation of short-term conservative treatment in patients with tennis elbow (lateral epicondylitis): A prospective randomised, assessor-blinded trial (Doctoral dissertation, University of Central Lancashire).	Insufficient data

Supplementary File 11: Risk of bias assessment for individual studies

Author, Year	Random sequence generation	Allocation concealment	Blinding of participants/personnel	Blinding of outcome assessment	Incomplete outcome bias	Selective reporting	Other bias
1. Aceituno-Gómez et al 2019	Low risk	Unclear	Low risk	Low risk	Low risk	Low risk	High risk
2. Akkaya et al 2016	Low risk	Unclear	High risk	High risk	Low risk	Unclear	Low risk
3. Alfredson et al 1998	High risk	Unclear	High risk	Unclear	Low risk	Unclear	High risk
4. Alfredson et al 1999	Not applicable (quasi)	Not applicable (quasi)	Not applicable (quasi)	Not applicable (quasi)	Low risk	Unclear	High risk
5. Arias-Buría et al 2015	Low risk	Low risk	High risk	Low risk	Low risk	Unclear	Low risk
6. Arias--Buría et al 2017	Low risk	Low risk	Unclear	Low risk	Unclear	Low risk	High risk
7. Bae et al 2011	Not applicable (quasi)	Not applicable (quasi)	Unclear	Unclear	Unclear	Unclear	High risk
8. Bahr et al 2006	Low risk	Low risk	High risk	Low risk	Low risk	Unclear	Low risk
9. Balius et al 2016	Low risk	Low risk	Unclear	Low risk	Low risk	Unclear	Low risk
10. Bang et al 2000	Low risk	Unclear	Unclear	Low risk	Low risk	Unclear	Low risk
11. Başkurt et al 2011	Low risk	Unclear	High risk	Unclear	Low risk	Unclear	Low risk
12. Beyer et al 2015	Low risk	Low risk	Low risk	Low risk	Low risk	Low risk	High risk
13. Blume et al 2015	Unclear	Low risk	Low risk	Low risk	Low risk	Unclear	Low risk
14. Boudreau et al 2019	Low risk	Low risk	Low risk	Low risk	Low risk	Low risk	Low risk
15. Breda et al 2020	Low risk	Low risk	High risk	Low risk	Low risk	High risk	High risk
16. Brox et al 1999	High risk	High risk	High risk	High risk	No Data	No Data	No Data
17. Calis et al 2011	Low risk	Low risk	Unclear	Unclear	Low risk	Unclear	Unclear
18. Chaconas et al 2017	Low risk	Unclear	Unclear	Low risk	High risk	Unclear	High risk
19. Cheng et al 2007	High risk	High risk	Unclear	Unclear	Unclear	Unclear	High risk
20. Cho et al 2017	High risk	High risk	Unclear	Unclear	Low risk	Low risk	Unclear
21. De Jonge et al 2008	Unclear	Low risk	Low risk	Low risk	Low risk	Low risk	Low risk
22. De Oliveira 2021	Low risk	Low risk	Low risk	Low risk	Low risk	High risk	High risk
23. De Vos et al 2007	Low risk	Unclear	Low risk	Low risk	Unclear	Low risk	High risk

Author, Year	Random sequence generation	Allocation concealment	Blinding of participants/personnel	Blinding of outcome assessment	Incomplete outcome bias	Selective reporting	Other bias
24. Dejaco et al 2017	Low risk	Low risk	Low risk	Low risk	Low risk	High risk	Low risk
25. Devereaux et al 2016	Low risk	High risk	High risk	High risk	High risk	Unclear	Low risk
26. Dimitrios et al 2012	Not applicable (quasi)	Not applicable (quasi)	Low risk	Low risk	Low risk	Unclear	High risk
27. Dimitrios et al 2013	Not applicable (quasi)	Not applicable (quasi)	Low risk	Low risk	Low risk	Unclear	High risk
28. Dupuis et al 2018	Low risk	Low risk	High risk	Low risk	Low risk	Low risk	High risk
29. Engebretsen et al 2009	Low risk	Low risk	High risk	Low risk	Low risk	Unclear	Low risk
30. Engebretsen et al 2011	Low risk	Low risk	High risk	Low risk	Low risk	Unclear	Low risk
31. Gatz et al 2020	Low risk	Low risk	Low risk	Low risk	Unclear	Unclear	High risk
32. Giray et al 2019	Low risk	Low risk	High risk	Low risk	Low risk	Low risk	High risk
33. Granviken et al 2015	Low risk	Low risk	Low risk	Low risk	Low risk	Low risk	Low risk
34. Hallgren et al 2014	High risk	Low risk	High risk	Low risk	Unclear	Low risk	Low risk
35. Hallgren et al 2017	Unclear	Unclear	Low risk	Low risk	Low risk	Low risk	Low risk
36. Heron et al 2017	Low risk	Low risk	Low risk	Low risk	High risk	High risk	Low risk
37. Hotta et al 2020	Low risk	Low risk	High risk	Low risk	Low risk	Low risk	High risk
38. Johansson et al 2005	Low risk	Unclear	Unclear	Low risk	Low risk	Unclear	High risk
39. Jonsson et al 2005	Unclear	Unclear	Low risk	Unclear	High risk	Unclear	High risk
40. Jonsson 2009	Unclear	Unclear	Unclear	Unclear	Unclear	Unclear	Unclear
41. Juul-Kristensen et al 2019	Low risk	Low risk	Low risk	Low risk	Low risk	Unclear	Low risk
42. Ketola et al 2009	Low risk	Low risk	Unclear	Low risk	Low risk	Unclear	High risk
43. Ketola et al 2013	Low risk	Low risk	High risk	Low risk	Low risk	Unclear	Low risk
44. Kim et al 2017	Low risk	Unclear	Unclear	Low risk	Unclear	Low risk	Low risk
45. Kim et al 2020	Low risk	Low risk	High risk	High risk	Low risk	Unclear	High risk
46. Knobloch et al 2007	Low risk	Low risk	Unclear	Unclear	High risk	Unclear	High risk

**Author, Year**	**Random sequence generation**	**Allocation concealment**	**Blinding of participants/personnel**	**Blinding of outcome assessment**	**Incomplete outcome bias**	**Selective reporting**	**Other bias**
47. Knobloch et al 2007	Unclear	Unclear	High risk	Low risk	Unclear	Unclear	High risk
48. Knobloch et al 2008	Unclear	Low risk	Unclear	Unclear	Unclear	Unclear	High risk
49. Kongsgaard et al 2009	Low risk	Low risk	High risk	Low risk	Low risk	Unclear	Low risk
50. Kromer et al 2014	Low risk	Low risk	High risk	High risk	Low risk	Low risk	Low risk
51. Kromer et al 2013	Low risk	Low risk	Low risk	Low risk	Low risk	Low risk	Low risk
52. Littlewood et al 2016	Low risk	Low risk	High risk	High risk	Unclear	Unclear	High risk
53. Ludewig et al 2003	Low risk	Unclear	High risk	Unclear	Low risk	Unclear	Low risk
54. Luginbuhl et al 2008	Unclear	Unclear	Unclear	Unclear	Unclear	Unclear	High risk
55. Maenhout et al 2013	Unclear	High risk	High risk	High risk	Low risk	Unclear	Low risk
56. Mafi et al 2001	Low risk	Unclear	Unclear	Unclear	Unclear	Unclear	High risk
57. Manias et al 2006	High risk	High risk	High risk	High risk	Low risk	Unclear	Unclear
58. Martinez-Silvestrini et al 2005	Unclear	Unclear	Unclear	Unclear	Low risk	Unclear	High risk
59. Marzetti et al 2014	Low risk	Low risk	Low risk	Low risk	Low risk	Low risk	Low risk
60. McCormack et al 2016	Low risk	Low risk	Unclear	Unclear	Low risk	Low risk	Low risk
61. Melegati et al 2000	Unclear	Unclear	Unclear	Unclear	Unclear	Unclear	High risk
62. Mulligan et al 2016	Low risk	Low risk	Low risk	Low risk	Low risk	Unclear	High risk
63. Nishizuka et al 2017	Low risk	Low risk	High risk	Unclear	Low risk	Unclear	High risk
64. Nørregaard et al 2007	Low risk	Low risk	Unclear	Unclear	Unclear	Unclear	High risk
65. Nowotny et al 2018	Low risk	Unclear	Low risk	Low risk	High risk	Unclear	High risk
66. Østerås et al 2010	Low risk	Low risk	High risk	High risk	Low risk	Unclear	High risk
67. Paavola et al 2018	Low risk	Low risk	Low risk	Low risk	Low risk	Low risk	Low risk
68. Park et al 2010	Low risk	Unclear	Unclear	Unclear	Unclear	Unclear	High risk
69. Pearson et al 2012	Unclear	Unclear	High risk	Unclear	Low risk	Unclear	High risk

Author, Year	Random sequence generation	Allocation concealment	Blinding of participants/personnel	Blinding of outcome assessment	Incomplete outcome bias	Selective reporting	Other bias
70. Pearson et al 2018	Low risk	Low risk	High risk	Low risk	Low risk	Low risk	High risk
71. Pekyavas et al 2016	Low risk	Low risk	High risk	Low risk	Unclear	Unclear	Low risk
72. Petersen et al 2007	Low risk	Unclear	Unclear	Unclear	Unclear	Unclear	High risk
73. Peterson et al 2011	Low risk	Low risk	Unclear	High risk	Low risk	Low risk	Low risk
74. Peterson et al 2014	Low risk	Unclear	Low risk	High risk	Low risk	Low risk	Low risk
75. Polimeni et al 2003	Unclear	Unclear	High risk	Low risk	Unclear	Unclear	Unclear
76. Praet et al 2019	Low risk	Low risk	Low risk	Low risk	Low risk	Unclear	Low risk
77. Rabusin et al 2020	Low risk	Low risk	High risk	High risk	Low risk	Low risk	High risk
78. Reyhan et al 2020	Low risk	Low risk	Unclear	Unclear	Low risk	Unclear	High risk
79. Rio et al 2017	Low risk	Low risk	Low risk	Low risk	Low risk	Low risk	High risk
80. Romero-Morales et al 2020	Unclear	Unclear	Unclear	Unclear	Low risk	Low risk	High risk
81. Rompe et al 2007	Low risk	Low risk	Unclear	Low risk	Low risk	Unclear	Low risk
82. Rompe et al 2008	Low risk	Low risk	Unclear	Low risk	Low risk	Unclear	Unclear
83. Rompe et al 2009	Low risk	Low risk	High risk	Low risk	Low risk	Unclear	Low risk
84. Roos et al 2004	Low risk	Unclear	Unclear	Low risk	Low risk	Unclear	Low risk
85. Şenbursa et al 2011	Low risk	Unclear	Unclear	Unclear	Low risk	Unclear	Low risk
86. Seven et al 2017	Low risk	Low risk	High risk	Low risk	High risk	Unclear	Low risk
87. Sevier et al 2015	Low risk	Unclear	High risk	High risk	High risk	Unclear	High risk
88. Silbernagel et al 2007	Low risk	Low risk	High risk	High risk	Low risk	Unclear	Low risk
89. Silbernagel et al 2001	Unclear	Unclear	Unclear	Unclear	Unclear	Unclear	High risk
90. Şimşek et al 2013	Unclear	Unclear	Unclear	Low risk	Unclear	Unclear	Unclear
91. Stasinopoulos et al 2006	Not applicable (quasi)	Not applicable (quasi)	Unclear	Low risk	Low risk	Unclear	High risk
92. Stasinopoulos et al 2010	Not applicable (quasi)	Not applicable (quasi)	Low risk	Low risk	Low risk	Unclear	High risk
93. Stasinopoulos et al 2017	Low risk	Unclear	Low risk	Low risk	Low risk	Unclear	High risk
94. Stefansson et al	Low risk	Unclear	High risk	Low risk	High risk	Unclear	Low risk

Author, Year	Random sequence generation	Allocation concealment	Blinding of participants/personnel	Blinding of outcome assessment	Incomplete outcome bias	Selective reporting	Other bias
2019							
95. Steunebrink et al 2013	Low risk	Low risk	Low risk	Low risk	Low risk	Unclear	Low risk
96. Stevens et al 2014	Unclear	Unclear	High risk	High risk	Unclear	Unclear	High risk
97. Svernlov et al 2001	Not applicable (quasi)	Not applicable (quasi)	Unclear	Unclear	Unclear	Unclear	High risk
98. Tahran et al 2020	Low risk	Unclear	Low risk	Low risk	Low risk	Unclear	Low risk
99. Tonks et al 2007	Low risk	Low risk	Low risk	High risk	High risk	Low risk	Low risk
100. Turgut et al 2017	Low risk	Unclear	Unclear	Unclear	High risk	Unclear	Low risk
101. Vallés-Carrascosa et al 2018	Low risk	Low risk	Low risk	High risk	Low risk	Low risk	High risk
102. vanArk et al 2016	Low risk	Low risk	Low risk	Unclear	Unclear	Low risk	Low risk
103. Vinuesa-Montoya et al 2017	Low risk	Low risk	High risk	Low risk	Low risk	Low risk	Low risk
104. Visnes et al 2005	Low risk	Low risk	High risk	Low risk	Unclear	Unclear	Unclear
105. Vuvan et al 2020	Low risk	Low risk	High risk	High risk	Low risk	Low risk	Low risk
106. Walther et al 2004	Unclear	Unclear	Unclear	Unclear	Low risk	Unclear	Unclear
107. Wegener et al 2016	Low risk	Low risk	High risk	Low risk	Low risk	Unclear	High risk
108. Wen et al 2011	Unclear	Unclear	Low risk	Unclear	High risk	Unclear	High risk
109. Werner et al 2002	Low risk	Unclear	Unclear	Unclear	Unclear	Unclear	High risk
110. Wiedmann et al 2017	Low risk	Low risk	Unclear	Unclear	Unclear	Unclear	High risk
111. Yelland et al 2011	Low risk	Low risk	High risk	Low risk	Low risk	Unclear	Low risk
112. Yerlikaya et al 2018	Low risk	Unclear	Low risk	Low risk	Unclear	Unclear	High risk
113. Young et al 2005	Unclear	Unclear	High risk	Low risk	High risk	Unclear	High risk
114. Yu et al 2013	Low risk	Low risk	Low risk	Unclear	Low risk	Unclear	Unclear

Supplementary file 12: Forest plot of effect sizes illustrated across studies

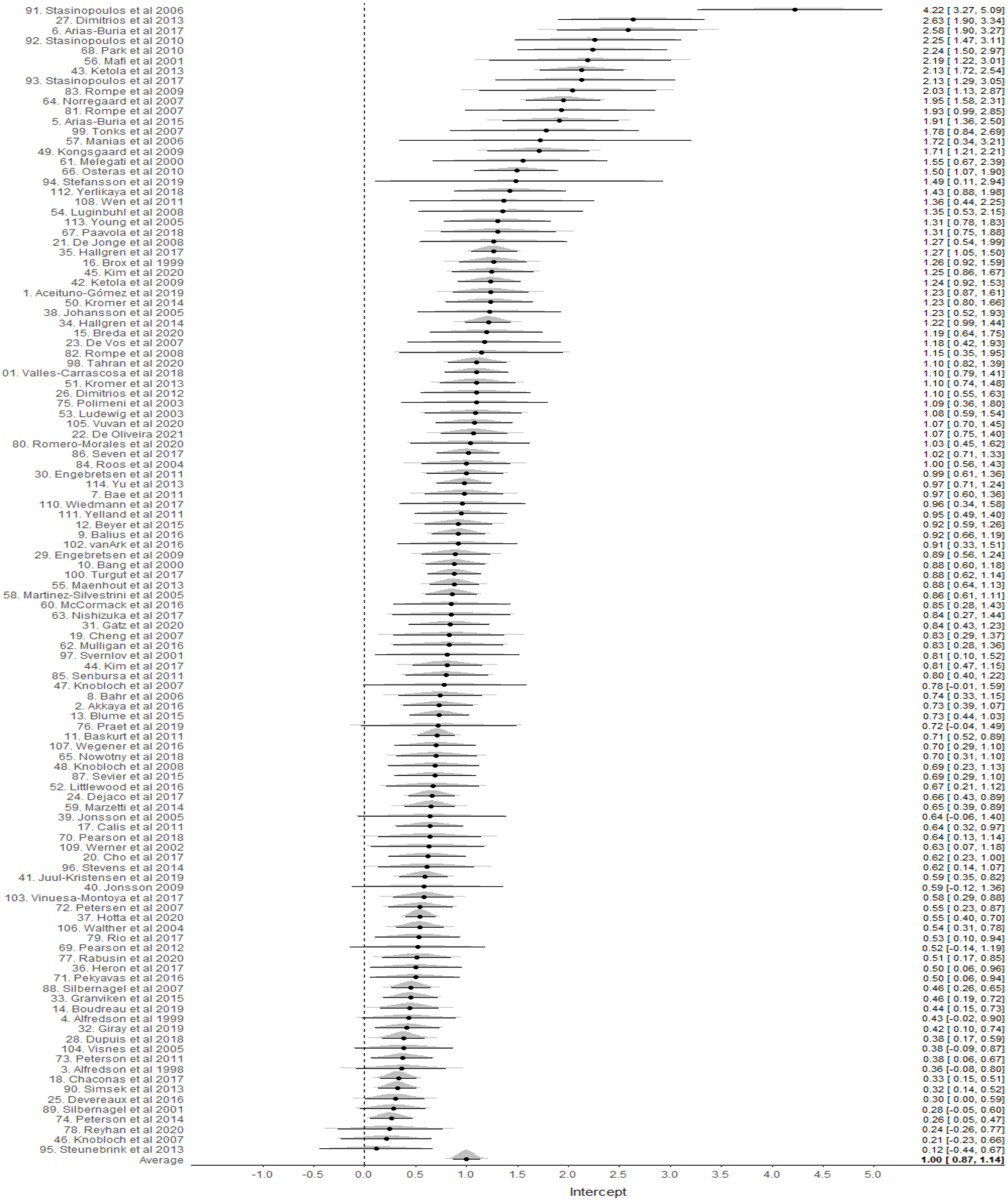

Distributions represent "shrunken estimates" based on all relevant effect sizes, the random effects model fitted, and borrowing of information across studies to reduce uncertainty. Black circles and connected intervals represent the median value and 95% credible intervals for the shrunken estimates.

Supplementary file 13: Sensitivity analyses

Sensitivity analysis checking the influence of study type was conducted by comparing the distribution of effect sizes and the small, medium, and large threshold obtained when analysing all data (114 studies) and data from randomised control studies only (110 studies). The plot below illustrates the empirical distributions using a density plot of the directly calculated effect sizes and the small medium and large thresholds:
All data - Small: (0.25-quantile$_{0.5}$ = 0.34 [95%CrI: 0.31 to 0.37]); Medium: (0.5-quantile$_{0.5}$ = 0.73 [95%CrI: 0.70 to 0.77]); and Large: (0.75-quantile$_{0.5}$ = 1.21 [95%CrI: 1.17 to 1.27]).
Randomised control trials only - Small: (0.25-quantile$_{0.5}$ = 0.33 [95%CrI: 0.29 to 0.36]); Medium: (0.5-quantile$_{0.5}$ = 0.71 [95%CrI: 0.67 to 0.75]); and Large: (0.75-quantile$_{0.5}$ = 1.17 [95%CrI: 1.12 to 1.24]).

Effect size distributions across the whole data set and randomised controlled trials only with identification of small, medium, and large thresholds.

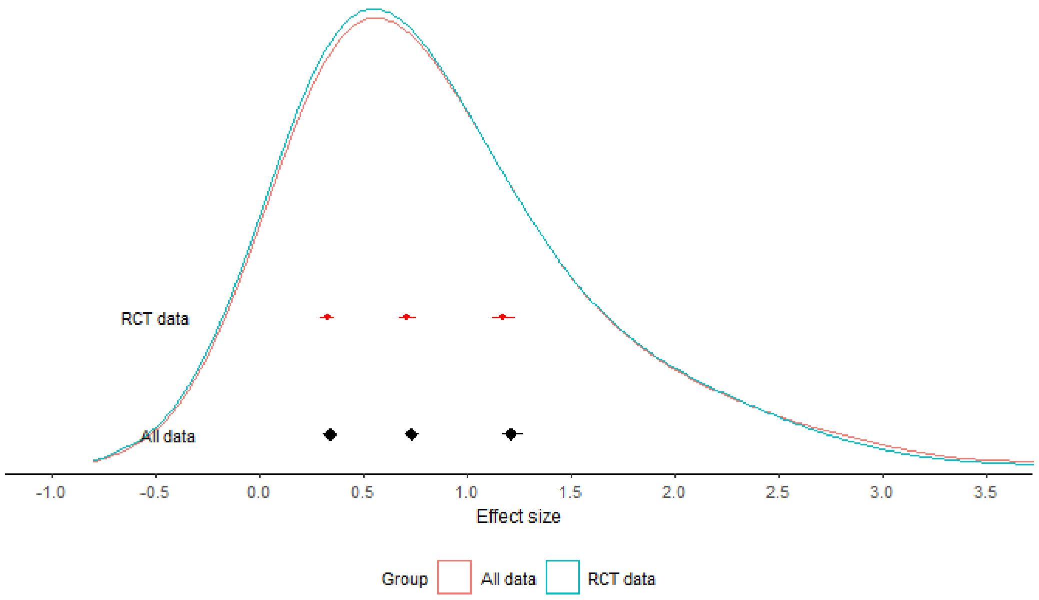

Curve represents density plot of empirical effect size distribution. Diamonds with intervals represent small, medium, and large thresholds with credible intervals (black: all data; red: RCT only).

Sensitivity analysis checking the influence of study quality was conducted by comparing the distribution of effect sizes and the small, medium, and large threshold obtained when analysing data from studies identified as low risk of bias (60 studies) and from studies identified as high risk of bias only (54 studies). The plot below illustrates the empirical distributions using a density plot of the directly calculated effect sizes and the small medium and large thresholds:
Low risk of bias - Small: (0.25-quantile$_{0.5}$ = 0.37 [95%CrI: 0.33 to 0.41]); Medium: (0.5-quantile$_{0.5}$ = 0.76 [95%CrI: 0.72 to 0.80]); and Large: (0.75-quantile$_{0.5}$ = 1.22 [95%CrI: 1.16 to 1.28]).
High risk of bias - Small: (0.25-quantile$_{0.5}$ = 0.30 [95%CrI: 0.24 to 0.34]); Medium: (0.5-quantile$_{0.5}$ = 0.68 [95%CrI: 0.61 to 0.73]); and Large: (0.75-quantile$_{0.5}$ = 1.16 [95%CrI: 1.09 to 1.25]).

Effect size distributions across studies identified as low or high risk of bias with identification of small, medium, and large thresholds.

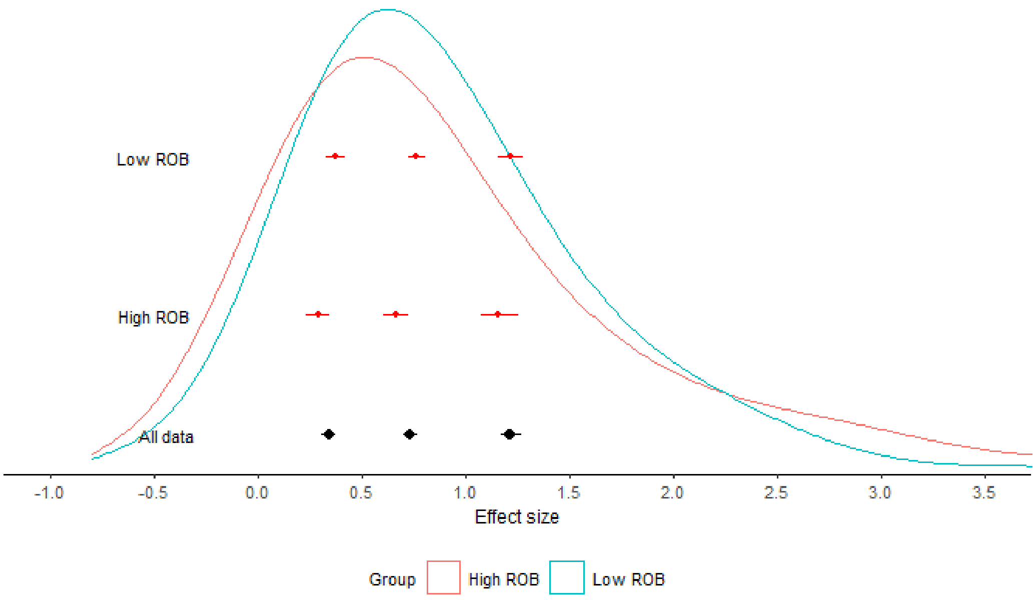

Curve represents density plot of empirical effect size distribution. Diamonds with intervals represent small, medium, and large thresholds with credible intervals (black: all data; red: different study quality).
